# Synthesis and Antitumor Mechanism of Emodin-Derived Transition Metal Complexes

**DOI:** 10.3390/molecules31152682

**Published:** 2026-07-31

**Authors:** Yumin Pan, Ying Rui, Biqun Zou, Xiaoteng Jing, Ruijie He, Jianyi Liang, Fangyao Li

**Affiliations:** 1 School of Chemistry and Life Sciences, Guilin Normal University, Guilin 541199, China; panyumin@mail.glnc.edu.cn (Y.P.); ruiying@mail.glnc.edu.cn (Y.R.); jingxiaoteng@mail.glnc.edu.cn (X.J.); 18897730565@163.com (J.L.); 2 Guangxi Key Laboratory of Functional Phytochemicals and Continuous Utilization of Resources, Guangxi Institute of Botany, Guangxi Zhuang Autonomous Region and Chinese Academy of Sciences, Guilin 541006, China; 3 College of Pharmacy, Guilin Medical University, Guilin 541100, China

**Keywords:** emodin derivatives, azacyclic ligands, transition metal complexes, antitumor activity

## Abstract

In this study, emodin was used as the starting material to synthesize two novel ligands, **L1** and **L2**, containing bipyridine or phenanthroline moieties. Seven metal complexes (**1–7**) were obtained through coordination with Co, Rh, Ru, and Pt ions. Their structures were confirmed via NMR, HRMS, UV–Vis, IR, HPLC and SC-XRD analyses. The MTT assay showed that **L1** and complex **2** selectively inhibited HepG-2 cells, with IC_50_ values of 8.77 μM and 6.10 μM, respectively. Both compounds exhibited stronger activity than cisplatin (9.05 μM) and low toxicity toward normal 293T cells. Mechanistic studies demonstrated that they induced G2/M phase arrest by regulating Cyclin B1 and P21 expression, which was accompanied by ROS and Ca^2+^ accumulation, mitochondrial membrane potential disruption, and activation of the Caspase-9/3 cascade. Complex **2** showed superior antitumor activity to **L1**, and this finding was further supported by molecular docking analysis. In sum, Rh(III) complex **2** was identified as a selective and mechanistically defined anti-hepatocellular carcinoma lead compound, providing a potential strategy for the development of natural product-based metal anticancer agents.

## 1. Introduction

Cancer has long been regarded as one of the leading threats to human health and life [1,2,3,4,5]. Against the backdrop of a mounting global cancer burden [6,7,8], substantial advances have been made in oncological therapeutics, and modalities such as chemotherapy, targeted combination regimens, and immunotherapy have been progressively established and broadly implemented. Despite these efforts, cancer incidence remains persistently high. Chemotherapy is routinely adopted in clinical settings, yet its application is constrained by several drawbacks. Pronounced toxicity and a tendency to provoke drug resistance are frequently encountered with conventional agents, resulting in markedly compromised patient compliance. The exploration of novel antitumor candidates featuring high efficacy, superior selectivity, low toxicity, and distinct mechanisms of action has, thus, emerged as a pressing priority.

In the field of drug discovery, lead compounds endowed with biological activities have been routinely uncovered from natural product libraries. Emodin (1,3,8-trihydroxy-6-methylanthraquinone), a bioactive anthraquinone, is isolated from traditional Chinese medicinal herbs, including Polygonum multiflorum and Rheum palmatum [9,10,11]. Emodin’s antitumor, anti-inflammatory, and immunomodulatory properties have been extensively documented in pharmacological investigations, wherein tumor progression was found to be suppressed through diverse mechanisms [12]. In these studies, emodin elicited cell cycle arrest in malignant cells, and the mitochondria-mediated intrinsic apoptotic pathway was concomitantly triggered. Tumor angiogenesis was likewise impaired, with the multidrug resistance in cancer cells being effectively reversed [13,14,15,16,17,18]. Furthermore, previous studies had demonstrated that metal complexes derived from emodin exhibited enhanced anticancer activities due to their improved interactions with DNA. In particular, copper-based emodin complexes were reported to achieve improved therapeutic effects through stronger DNA-binding abilities [19], and manganese-based emodin complexes had been shown to induce morphological alterations, cell cycle arrest, and apoptosis in cancer cells [20]. These findings indicate that emodin possesses considerable antitumor potential, providing a valuable structural framework for the development of novel anticancer agents. However, the clinical application of emodin has been significantly limited by several intrinsic disadvantages. Its poor water solubility and low bioavailability in vivo restrict its pharmacological efficacy. Moreover, emodin exhibits certain cytotoxic effects toward normal cells [21,22,23]. These inherent limitations have greatly compromised its therapeutic value and hindered further drug development.

Since cisplatin was introduced into clinical practice, the development of metal-based antitumor complexes received considerable attention [24]. Platinum-based agents, including carboplatin and oxaliplatin, were widely applied in the treatment of various malignant tumors and produced favorable clinical outcomes [25,26]. Despite these advances, their clinical application was greatly restricted by severe adverse effects and the frequent emergence of drug resistance. Such limitations prompted continuous efforts to develop non-platinum metal complexes with improved therapeutic potential. In this context, transition metal complexes containing rhodium (Rh), ruthenium (Ru), cobalt (Co), iron (Fe), and other metal centers were extensively investigated. Their growing interest was attributed to their diverse coordination geometries, characteristic redox properties, and distinct antitumor mechanisms [27,28]. Qin et al. [29,30] designed and synthesized a series of ruthenium(II) complexes derived from jatrorrhizine and berberine. These complexes were demonstrated to target telomerase, induce apoptosis in human bladder cancer T-24 cells, and achieve a higher tumor inhibition rate than cisplatin in vivo [31,32]. Based on the findings, metal coordination of emodin was proposed as a rational strategy for the design of efficacious metal-based antitumor agents with reduced toxicity.

5-Amino-2,2′-bipyridine, 5-amino-1,10-phenanthroline, and their derivatives are regarded as important nitrogen-containing heterocyclic chelators, and these compounds have attracted considerable interest in medicinal chemistry due to their pronounced metal-coordinating capacity and broad pharmacological profiles. Amino-substituted bipyridine and phenanthroline derivatives, along with their metal complexes, had previously been shown to exhibit antibacterial, antioxidant, and antitumor activities [33]. In this study, emodin derivatives were tethered to bipyridine or phenanthroline moieties via substitution reactions, aiming to integrate the beneficial antitumor properties of both pharmacophores. The suitable coordination sites for subsequent complexation with transition metal ions were thus furnished.

On this basis, a series of emodin-derived compounds bearing 5-amino-2,2′-bipyridine or 5-amino-1,10-phenanthroline scaffolds were designed and prepared, and these derivatives were subsequently exploited as polydentate ligands for coordination with transition metal ions. A panel of structurally novel emodin–bipyridine and emodin–phenanthroline transition metal complexes was thereby assembled. Structural characterization of the resulting complexes was performed, with their in vitro antitumor activities and underlying mechanisms being further interrogated in a systematic manner.

## 2. Results

### 2.1. Synthesis and Characterization

**Scheme 1:**
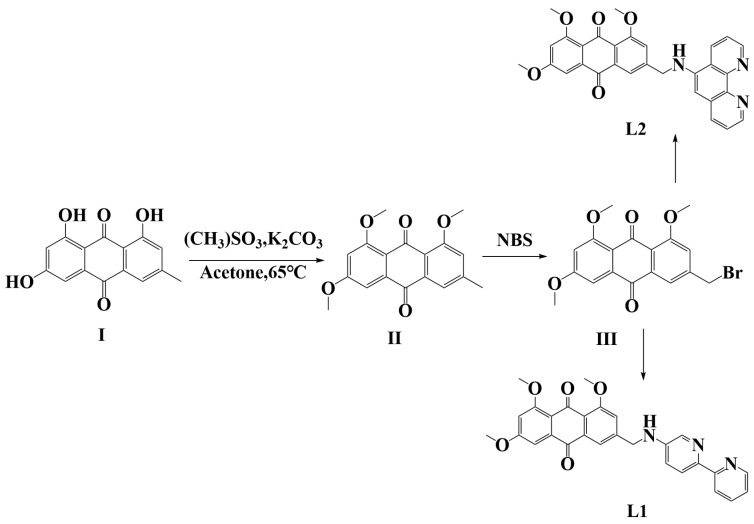
Synthetic routes of ligands **L1** and **L2.**

Emodin was used as the starting material. Methylation of the parent skeleton was first conducted, after which a bromine atom was incorporated into the framework through a substitution reaction. Two amines, namely, 5-amino-2,2′-bipyridine and 5-amino-1,10-phenanthroline, were subsequently introduced via nucleophilic substitution, affording two target ligands. These resulting compounds were characterized as 3-(([2,2′-bipyridin]-5-ylamino)methyl)-1,6,8-trimethoxyanthracene-9,10-dione (**L1**) and 3-(((1,10-phenanthrolin-5-yl)amino)methyl)-1,6,8-trimethoxyanthracene-9,10-dione (**L2**) (Scheme 1).

Ligands **L1** and **L2** were subsequently subjected to coordination reactions with four transition metal salts containing Pt, Ru, Rh, or Co. Each ligand was independently reacted with the corresponding metal precursor, whereby seven metal complexes were obtained, namely, [Co(**L1**)(NO_3_)_2_(H_2_O)_2_] (**1**), [Rh(**L1**)Cl_3_CH_3_OH] (**2**), [Ru(**L1**)Cl_3_CH_3_OH] (**3**), [Pt(**L1**)(DMSO)_2_]Cl_2_(**4**), [Rh(**L2**)(DMSO)Cl_2_(H_2_O)] (**5**), [Ru(**L2**)Cl_2_(DMSO)_2_] (**6**), and [Pt(**L2**)Cl_2_)](**7**) (Scheme 2). The structures of all target compounds were unambiguously confirmed via ^1^H NMR, ^13^C NMR, and HRMS analyses (Figures S1–S27). To investigate the stability of the two ligands and seven transition metal complexes, UV–Vis absorption spectra were recorded within the same wavelength range after the compounds had been maintained at room temperature for 0, 12, 24, and 48 h. No obvious red shifts or blue shifts in the absorption peaks were observed, and no additional absorption bands appeared during the monitoring period. These results suggested that all nine compounds remained stable at room temperature for at least 48 h (Figures S28–S45). The purity of all complexes used in the biological experiments was >95%, as verified using HPLC (Figures S46–S54).

### 2.2. Crystal Structure of Complex ***2***

**Figure 1:**
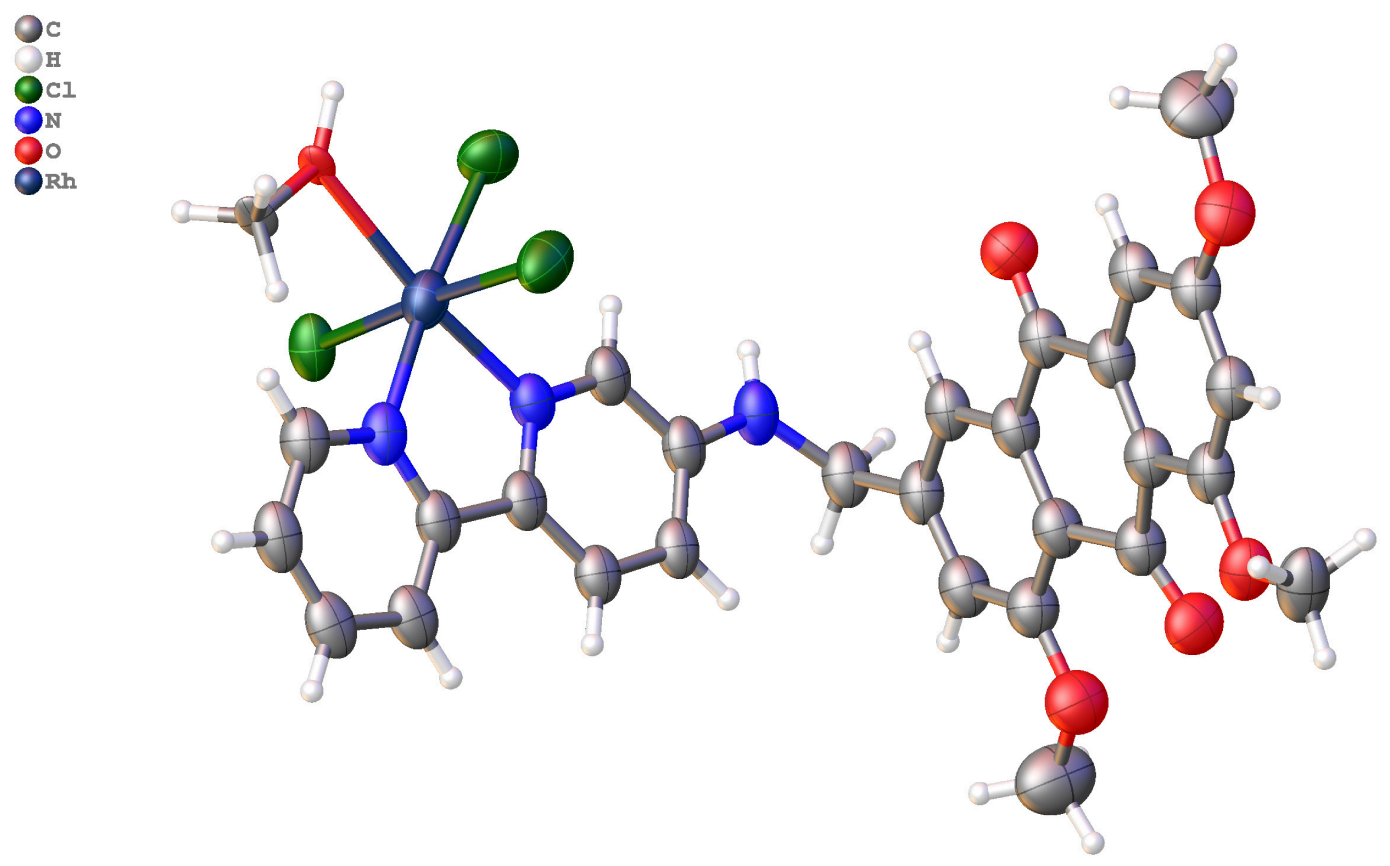
Crystal structure of complex **2** (CCDC:2256789).

The single-crystal structure of complex **2** was elucidated using X-ray diffraction analysis (Figure 1). A six-coordinate environment was adopted by the central Rh(III) ion, through which a distorted octahedral geometry was established. Coordination was furnished collaboratively by a tridentate ligand and chloride ions, with two nitrogen atoms (N2 and N3) and one oxygen atom (O6 or O8) of the ligand being bound to the Rh(III) center. Several characteristic bond parameters were revealed from the crystallographic data. The Rh–N bond lengths were determined to be 2.032(6) Å [Rh1–N2] and 2.040(7) Å [Rh1–N3], while the Rh–O distances were measured as 2.315(7) Å [Rh1–O6] and 2.158(13) Å [Rh1–O8]. The remaining coordination sites were occupied by chlorides, with the corresponding Rh–Cl bond lengths being Rh1–Cl1 2.353(2) Å, Rh1–Cl2 2.353(2) Å, Rh1–Cl3 2.383(4) Å, and Rh1–Cl4 2.310(8) Å. A Cl2–Rh1–Cl1 angle of 176.30(8)° was recorded, indicating a slight deviation from ideal collinearity; therefore, a minor distortion of the octahedral geometry was manifested in complex **2**. Detailed crystallographic parameters along with selected bond lengths and angles are presented in Tables S1–S4. The bond angle of Cl2–Rh1–Cl1 deviates slightly from the ideal 180°, indicating a minor distortion in the coordination environment.

### 2.3. In Vitro Cytotoxicity Study

The antiproliferative activities of the two ligands (**L1** and **L2**) and the seven metal complexes (**1–7**) were preliminarily assessed using an MTT assay at 20 μM. Human hepatocellular carcinoma (HepG-2), lung cancer (A549), colon cancer (SW-480), breast cancer (MCF-7), oral cancer (Cal-27), and bladder cancer (T24) cell lines were used in the evaluation, while cytotoxicity toward the normal human embryonic kidney cell line 293T was examined in parallel under identical conditions (Table S5).

Based on the preliminary screening, ligand **L1**, together with complexes **2** and **7**, was found to exert appreciable inhibitory effects on several tumor cell lines; these three compounds were further evaluated. Their IC_50_ values against HepG-2, SW-480, MCF-7, Cal-27, and 293T cells were subsequently determined, with cisplatin being included as the positive control (Table 1). Pronounced antiproliferative activities toward HepG-2 cells were displayed by **L1** and complex **2**, with IC_50_ values of 8.77 ± 0.08 μM and 6.10 ± 0.06 μM, respectively, both of which were lower than that of cisplatin (9.05 ± 0.40 μM) under the same conditions. In the normal 293T cell line, IC_50_ values of 51.69 ± 0.01 μM and 40.20 ± 1.16 μM were recorded for **L1** and complex **2**, respectively, whereas a substantially lower value of 14.52 ± 1.21 μM was obtained for cisplatin. The selectivity index toward HepG-2 cells, calculated as the ratio of IC_50_(293T) to IC_50_(HepG-2), was determined to be 5.89 for **L1** and 6.59 for complex **2**, indicating that both compounds exhibit favorable tumor selectivity. Accordingly, **L1** and complex **2** were chosen as representative candidates for the subsequent mechanistic studies.

### 2.4. Cell Cycle Arrest

**Figure 2:**
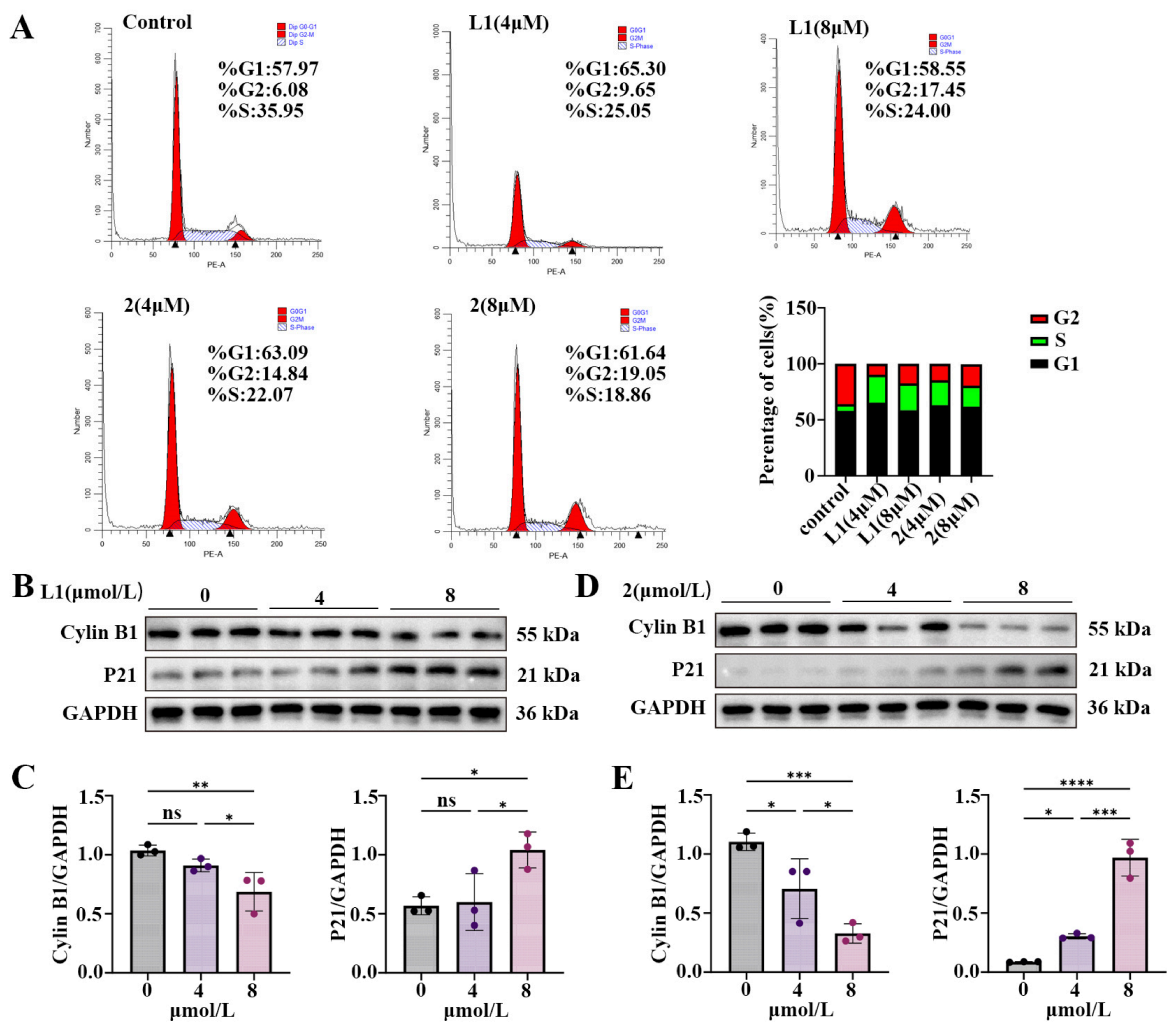
Effects of **L1** and **2** on cell cycle progression and related protein expression in HepG-2 cells. (**A**): HepG-2 cells were treated with **L1** or **2** for 48 h, and the cell cycle distribution was determined by flow cytometry. (**B**,**C**): Cells were exposed to graded concentrations of **L1** for 48 h, after which Cyclin B1 and P21 expression was assessed by Western blot and densitometrically quantified using Image J 1.8.0. (**D**,**E**): Cells were exposed to graded concentrations of **2** for 48 h, with Cyclin B1 and P21 expression being analyzed in the same manner. Data are presented as mean ± SD of three independent experiments (n = 3, ns *p* > 0.05; * *p* < 0.05; ** *p* < 0.01; *** *p* < 0.001; **** *p* < 0.0001).

Cell cycle progression has long been recognized as a pivotal process governing cell growth and proliferation, and its perturbation has been closely linked to growth inhibition and even cell death [34,35,36]. Flow cytometric analysis was performed to elucidate the impact of the tested compounds on this process. HepG-2 cells were exposed to graded concentrations of **L1** and complex **2** for 48 h, after which the cell cycle distribution was assessed. Pronounced G2-phase arrest was elicited by complex **2** in a concentration-dependent manner, and a comparable but less marked effect was likewise observed for **L1**. At identical concentrations, the arresti—ng effect of complex **2** was substantially superior to that of its parent ligand (Figure 2).

The G2-to-M phase transition is known to be predominantly governed by Cyclin B1, a key member of the cyclin family, while the cyclin-dependent kinase inhibitor P21 has been implicated in cellular response to DNA damage [37]. The expression levels of these two proteins were accordingly probed via Western blot analysis. A marked downregulation of Cyclin B1 and a concomitant upregulation of P21 were elicited by **L1** and complex **2** in a concentration-dependent manner. The modulatory effect of complex **2** was found to be more pronounced than that of **L1**, which was in accordance with the cell cycle distribution data. These results collectively indicate that the cell-cycle-arresting capacity of **L1** is potentiated upon metal coordination, leading to the enhanced capacity of complex **2** (Figure 2).

### 2.5. Apoptosis Detection

**Figure 3:**
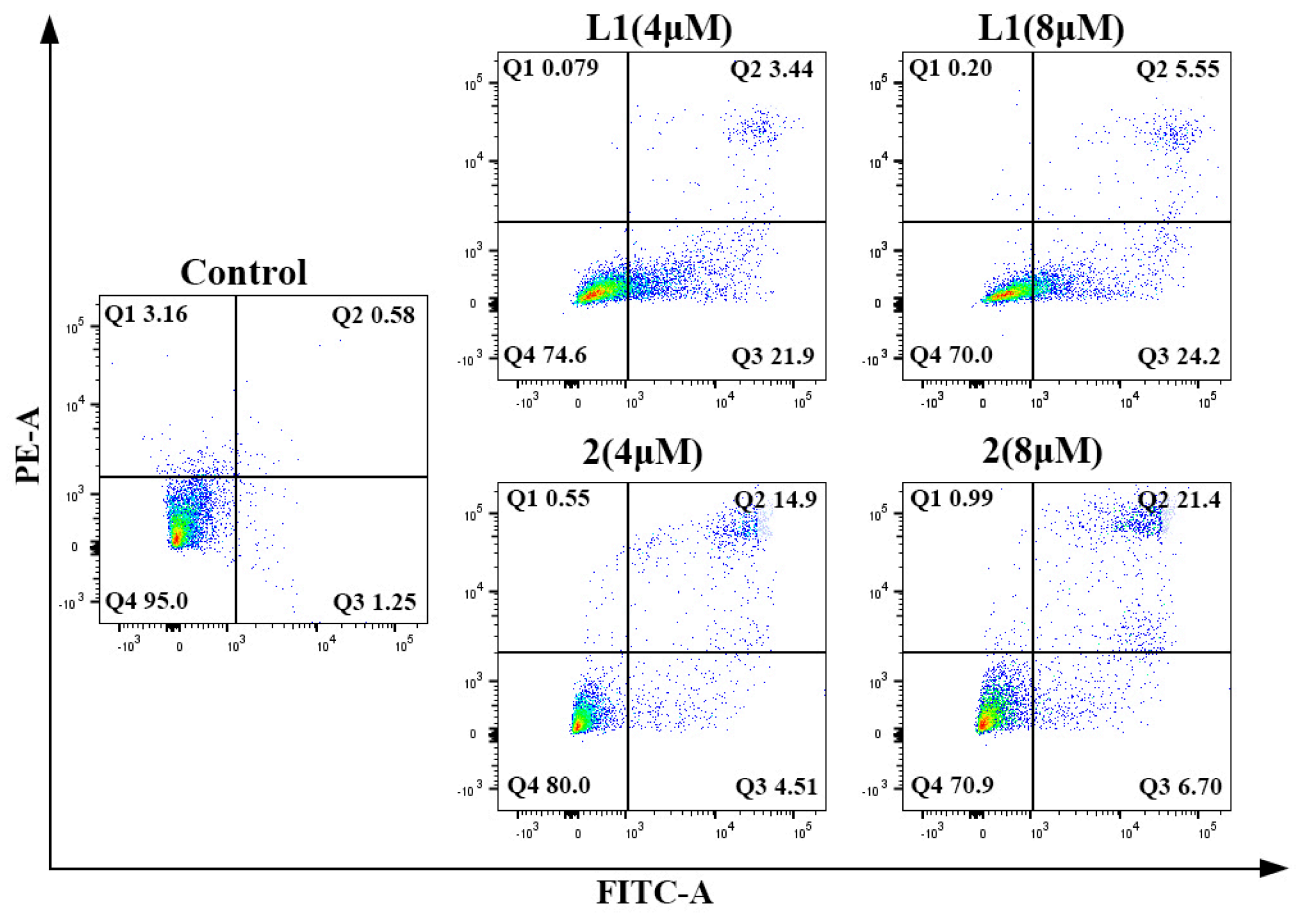
Effects of **L1** and **2** on apoptosis in HepG-2 cells. HepG-2 cells were exposed to **L1** or **2** for 48 h, and apoptosis was determined by flow cytometry.

Apoptosis is a form of programmed cell death featuring an active, highly ordered, and tightly regulated process governed by intracellular apoptosis-associated genes and is essential for the maintenance of physiological homeostasis. To assess the proapoptotic potential of **L1** and complex **2** in HepG-2 cells, Annexin V-FITC/PI double staining combined with flow cytometry was conducted, and the apoptotic ratios under different concentrations were quantified. Regarding the total apoptotic rate, a more potent induction was conferred by **L1** than by complex **2**; however, distinct preferences were observed with respect to the apoptotic stage modulated. Early-stage apoptosis was predominantly promoted by **L1**, whereas late-stage apoptosis was more markedly enhanced by complex **2** under identical conditions. Both compounds were thus shown to elicit concentration-dependent apoptosis in HepG-2 cells, and notable divergence was unveiled in their regulation of apoptotic progression despite their comparable proapoptotic capacities (Figure 3).

### 2.6. Mitochondrial Membrane Damage

**Figure 4:**
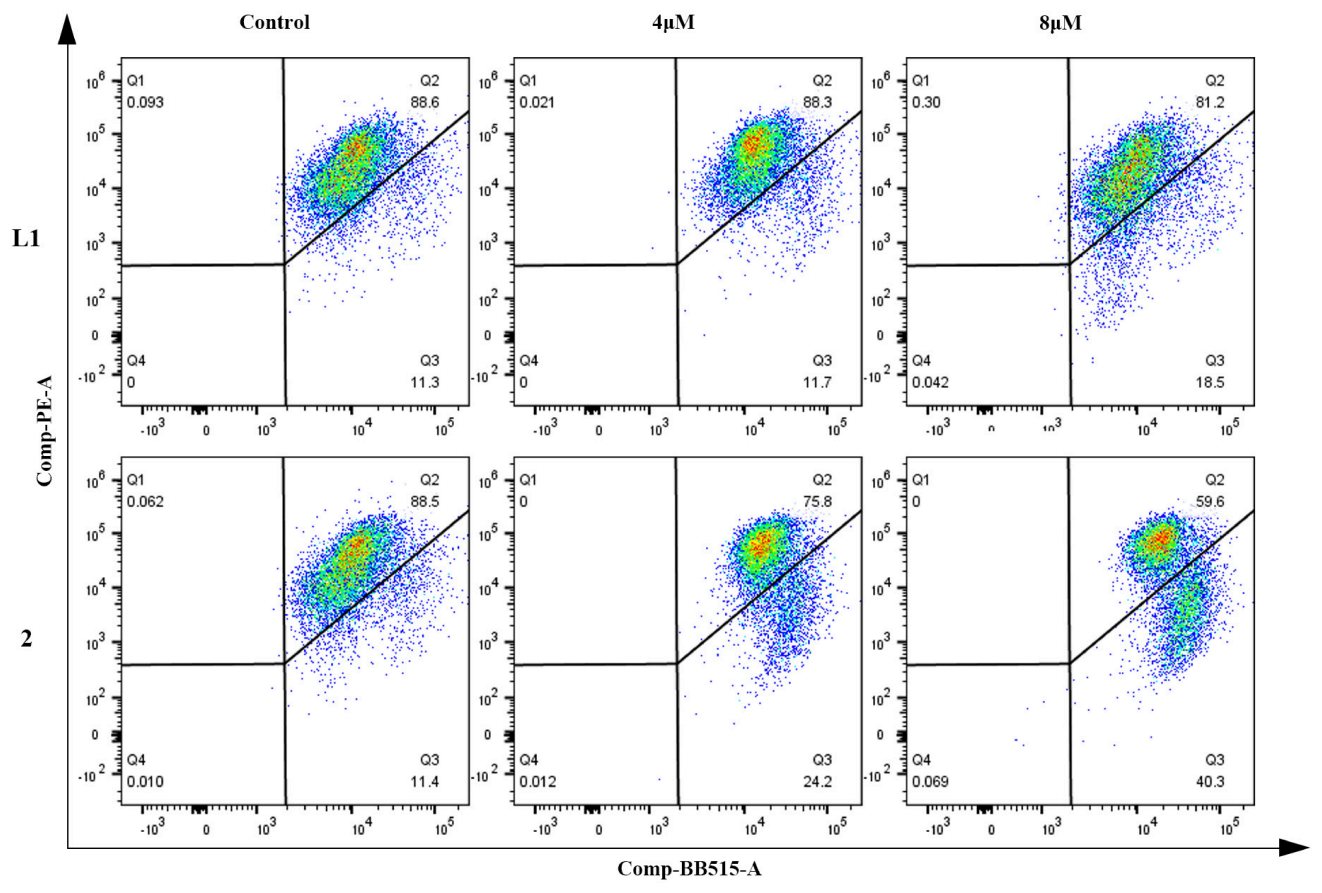
Effects of **L1** and **2** on the mitochondrial membrane potential of HepG-2 cells. HepG-2 cells were exposed to **L1** or **2** for 48 h, and the mitochondrial membrane potential was determined by flow cytometry.

A decline in mitochondrial membrane potential has been widely accepted as a hallmark event during the onset of apoptosis [38,39], which facilitates the release of proapoptotic factors from the mitochondria into the cytoplasm and promotes apoptotic progression. In the present study, JC-1 was used as a fluorescent probe to monitor mitochondrial membrane potential, and flow cytometric analysis was subsequently performed to elucidate the effects of **L1** and complex **2** on mitochondrial function in HepG-2 cells. After a 48 h treatment with JC-1, alterations in membrane potential were quantitatively determined. A significant reduction in mitochondrial membrane potential was elicited by both compounds, indicating mitochondria-mediated apoptosis. A more pronounced decrease was, however, induced by complex **2** relative to **L1** under identical conditions, demonstrating that the proapoptotic activity of **L1** was augmented upon metal coordination (Figure 4).

### 2.7. Measurement of Reactive Oxygen Species and Ca^2+^

**Figure 5:**
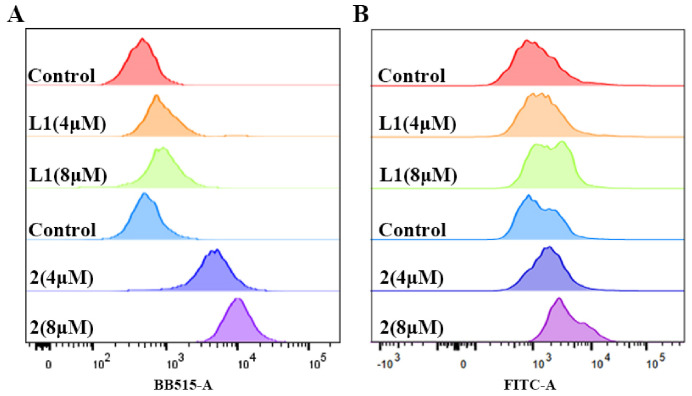
Effects of **L1** and **2** on intracellular ROS and Ca^2+^ release in HepG-2 cells. (**A**): HepG-2 cells were exposed to **L1** for 48 h, and ROS levels were determined by flow cytometry. (**B**): HepG-2 cells were exposed to **2** for 48 h, and Ca^2+^ release was assessed by flow cytometry.

Elevation of cytoplasmic Ca^2+^ has been frequently observed during apoptosis [40], and intracellular Ca^2+^ levels are closely linked to the generation and accumulation of reactive oxygen species (ROS) [41]. Excessive ROS is known to compromise the integrity of Ca^2+^-storage organelles, including mitochondrial and plasma membranes, thereby perturbing intracellular Ca^2+^ homeostasis. During this process, Ca^2+^ redistribution within cells, together with extracellular Ca^2+^ influx, is triggered, and sustained elevation of intracellular Ca^2+^ is subsequently elicited, ultimately culminating in apoptotic cell death [42]. To probe these effects, intracellular ROS generation and Ca^2+^ release in HepG-2 cells were monitored via flow cytometry after 48 h exposure to **L1** or complex **2**. Pronounced ROS accumulation was triggered by both compounds, with substantially higher ROS levels recorded for complex **2** than for **L1** under identical conditions (Figure 5). Intracellular Ca^2+^ release was likewise stimulated, and the fluorescence intensity was found to increase in a concentration-dependent manner relative to the control. A consistently stronger fluorescence signal was registered for complex **2** throughout the tested range, further substantiating that the **L1-**mediated proapoptotic activity was enhanced upon metal coordination (Figure 5).

### 2.8. Activation of the Caspase Signaling Pathway

**Figure 6:**
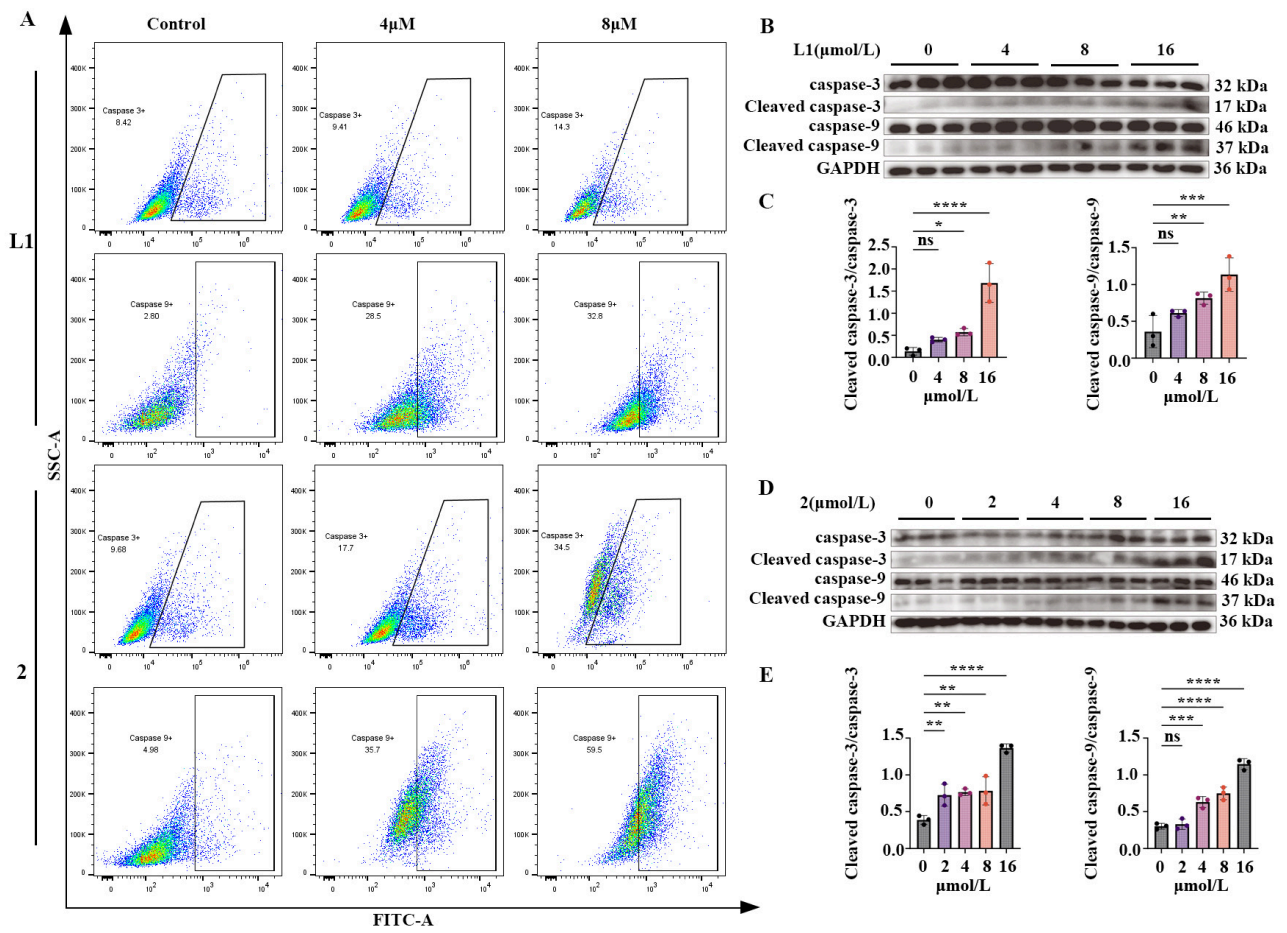
Effects of ligand **L1** and complex **2** on caspase-3/9 activation in HepG-2 cells. (**A**): HepG-2 cells were treated with **L1** or **2** for 48 h, and the activation of caspase-3/9 was determined by flow cytometry. (**B**,**C**): Cells were exposed to graded concentrations of **L1** for 48 h, after which the expression of caspase-3/9 and cleaved caspase-3/9 was assessed by Western blot and densitometrically quantified using Image J 1.8.0. (**D**,**E**): Cells were exposed to graded concentrations of **2** for 48 h, with the corresponding protein expression being analyzed in the same manner. The results are presented as the mean ± SD of three independent experiments (*n* = 3; ns *p* > 0.05, * *p* < 0.05, ** *p* < 0.01, and *** *p* < 0.001; **** *p* < 0.0001).

The caspase family constitutes key regulators during the execution phase of apoptosis [43,44,45], and caspase-9 is an initiator caspase in the apoptotic signaling cascade. Upon apoptotic stimulation, cytochrome c is released from the mitochondria into the cytoplasm, where it is associated with Apaf-1 and caspase-9 to assemble the apoptosome, further activating caspase-9. Downstream effector caspases such as caspase-3 are subsequently triggered, and apoptotic progression is propagated through the caspase cascade. To clarify the impact of **L1** and complex **2** on caspase activation, intracellular caspase-3 and caspase-9 activities in HepG-2 cells were determined via flow cytometry after a 48 h treatment. A concentration-dependent elevation of both caspases’ activities was elicited by the two compounds, with a markedly stronger activation conferred by complex **2** relative to the control and **L1** (Figure 6).

Western blot analysis was employed to further corroborate the modulatory effects of **L1** and complex **2** on the activated forms of apoptosis-related proteins, with particular emphasis being placed on cleaved caspase-3 and cleaved caspase-9, which serve as the activated forms of the executor and initiator caspases, respectively. A dose-dependent upregulation of cleaved caspase-3 and cleaved caspase-9 was elicited by both compounds in HepG-2 cells. Under identical concentration gradients, a substantially more potent activation was conferred by complex **2** than by **L1**, aligning with the flow cytometric data. These findings collectively indicate that activation of the mitochondrial apoptotic pathway is reinforced upon metal coordination of **L1**, which further activates the caspase cascade and thereby augments the proapoptotic potency of complex **2** (Figure 6).

### 2.9. Molecular Docking Verification of the Caspase Signaling Pathway

**Figure 7:**
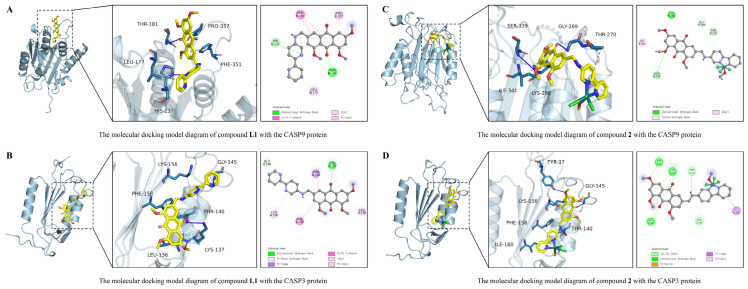
Docking models of **L1** and **2** with CASP3 and CASP9. (**A**): Docking pose of **L1** with CASP9. (**B**): Docking pose of **L1** with CASP3. (**C**): Docking pose of **2** with CASP9. (**D**): Docking pose of **2** with CASP3. Small molecules are shown as yellow sticks and proteins as blue cartoons. Hydrogen bonds are depicted as solid blue lines, π–π stacking as green dashed lines, π–cation interactions as orange dashed lines, and hydrophobic contacts as gray dashed lines.

Molecular docking has been routinely employed as a theoretical simulation approach for predicting receptor binding modes and affinities, wherein the structural features of the receptor and the interactions with the drug molecules are concurrently considered [46,47,48]. To elucidate the binding behavior of **L1** and complex **2** toward caspase-3 and caspase-9, molecular docking was carried out using Vina 1.2.3, and the interaction patterns with these key apoptotic targets were systematically investigated at the molecular level. Analysis of the docking scores revealed that the complex **2** and L1 derivatives exhibited more favorable binding energies toward CASP9 than toward CASP3, indicating a higher overall affinity of these ligands for the CASP9 target. Within each protein system, complex **2** consistently outperformed its **L1** counterpart. Specifically, in the CASP9 system, complex 2 achieved a docking score of −7.618 kcal/mol, which was 0.278 kcal/mol lower (more favorable) than that of the **L1** complex (−7.340 kcal/mol). Similarly, in the CASP3 system, complex **2** (with a docking score of −7.334 kcal/mol) showed an improvement of 0.402 kcal/mol over the **L1** complex (with a docking score of −6.932 kcal/mol). Collectively, these data demonstrate that the complex **2** derivative possesses superior binding affinity to both protein targets relative to the **L1** analog across (Table 2). Three-dimensional binding pattern analyses were subsequently performed based on the docking output. The molecular docking analysis of the CASP9 complexes revealed that hydrogen bonds serve as the predominant forces stabilizing the ligand–protein interactions. In the 1NW9 CASP9_L1 complex, two hydrogen bonds are formed involving THR-181 and HIS-237, which collectively constrain the ligand conformation within the binding pocket. In the 7RN9 CASP3_2 complex, a more extensive hydrogen-bonding network was observed, among which the LYS-280-mediated hydrogen bond contributes most significantly to the overall binding affinity. For the CASP3 system, the binding mode of 1NW9 CASP9_L1 is primarily governed by hydrophobic forces, with extensive contacts established between the ligand and five residues (LYS-137, PHE-158, THR-140, LYS-156, and LEU-136). In contrast, the binding stability of the 7RN9 CASP3_2 complex arises from the synergistic contributions of multiple hydrogen bonds and a π–cation interaction, highlighting a distinct energetic driving force compared to its 1NW9 CASP9_L1 counterpart (Figure 7).

## 3. Discussion

Emodin has attracted considerable attention as a natural anthraquinone because of its broad-spectrum antitumor activity [9,10,11,12]. However, its clinical application has been limited by poor aqueous solubility, low bioavailability, and undesirable toxicity toward normal cells [21,22,23]. Structural modification is therefore regarded as an effective approach to improving its pharmacological properties. In the present study, two ligands containing bipyridine or phenanthroline moieties were designed from the emodin scaffold, followed by coordination with Ru(II), Pt(II), Rh(III), and Co(II) ions to generate seven transition metal complexes. The successful synthesis of these compounds was confirmed via spectroscopic characterization, and SC-XRD showed that complex **2** adopted a six-coordinate distorted octahedral geometry around the Rh(III) center with **L1** acting as a multidentate chelating ligand. 

Compared with the previously reported Rh(III) complexes [49,50,51], complex 2 retained the characteristic distorted octahedral coordination geometry around the Rh(III) center. The Rh–N bond lengths remained within the range reported for Rh(III) complexes bearing bipyridine- or phenanthroline-derived ligands, indicating that substitution of the anthraquinone scaffold did not substantially perturb the primary Rh–N coordination sphere. In contrast, the two Rh–O bond lengths showed a more pronounced difference, suggesting that the oxygen donor atoms contributed unequally to coordination. This behavior differed from that of the reported [50,51] Rh(III) complexes dominated by nitrogen-donor ligands and was likely associated with the distinct electronic environments of the carbonyl oxygen atoms in the anthraquinone framework. In addition, the Cl2–Rh1–Cl1 bond angle was slightly smaller than the ideal value of 180°, which was consistent with the minor octahedral distortion observed in related Rh(III) complexes [50,51]. Such deviations have generally been attributed to the restricted bite angles of the chelating ligands and steric constraints within the coordination sphere. Therefore, the introduction of the anthraquinone ligand preserved the structural stability of the Rh(III) center while subtly modulating its local coordination environment.

The antiproliferative evaluation revealed clear differences between the synthesized compounds. **L1** and complex **2** exhibited the strongest inhibitory effects against HepG-2 cells and were therefore the objects of further investigation. Their IC_50_ values were lower than that of cisplatin, while their cytotoxicity toward normal 293T cells remained markedly reduced. More importantly, both compounds displayed substantially higher selectivity indices than cisplatin, indicating that the introduction of the Rh(III) center enhanced not only antiproliferative activity but also tumor selectivity. These findings suggest that rational coordination design can effectively optimize the biological properties of the parent ligand.

The enhanced activity of complex **2** can likely be attributed to the contribution of metal coordination to the parent ligand’s physicochemical and biological properties. Coordination with Rh(III) may have increased molecular stability and optimized the interaction between the ligand and intracellular targets, thereby improving biological responses. Similar activity enhancement has also been reported for other transition metal complexes derived from natural products, in which metal coordination was found to promote cellular uptake and strengthen target recognition [19,20]. The superior performance of complex **2,** therefore, appears to arise from the combined contribution of the emodin scaffold, the nitrogen-containing chelating ligand, and the Rh(III) coordination center rather than from a single structural component.

These observations demonstrated that coordination modification represents an effective strategy for improving the antitumor potential of emodin derivatives [24,25,26,27,28]. The structural characteristics of complex **2** were found to be closely associated with its favorable biological performance, providing a reasonable foundation for further mechanistic investigation.

The mechanistic investigation demonstrated that the antiproliferative activity of **L1** and complex **2** was closely associated with cell cycle regulation and mitochondria-mediated apoptosis. Both compounds induced G2/M phase arrest, where a more pronounced effect was induced by complex **2**. The concurrent downregulation of Cyclin B1 and upregulation of P21 indicated that inhibition of the Cyclin B1/CDK1 regulatory axis contributed to cell cycle arrest. Since progression through the G2/M checkpoint is essential for mitotic entry, disruption of this process suppresses cellular proliferation and increases susceptibility to apoptosis [34,35,36,37]. The stronger regulatory effect observed for complex **2** suggests that Rh(III) coordination enhances the ability of the parent ligand to interfere with cell cycle progression.

Differences in apoptotic behavior were also observed after metal coordination. Although both compounds induced apoptosis in a concentration-dependent manner, **L1** mainly promoted early apoptosis, whereas complex **2** preferentially increased late apoptotic cells. This finding indicates that Rh(III) coordination not only enhances cytotoxicity but also alters the apoptotic response. The observed mitochondrial membrane depolarization, accompanied by intracellular ROS accumulation and Ca^2+^ release, further demonstrates that mitochondrial dysfunction plays a central role in the apoptotic process. As excessive ROS production is widely recognized as a trigger of mitochondrial damage [52,53,54,55,56], the resulting loss of membrane potential and disruption of Ca^2+^ homeostasis are believed to facilitate irreversible apoptotic signaling [40,41,42]. The greater changes induced by complex **2** further supports the contribution of Rh(III) coordination to mitochondrial impairment.

The activation of the intrinsic apoptotic pathway was further verified via caspase activity assays and Western blot analysis. The increased expression of cleaved Caspase-9 and cleaved Caspase-3, together with elevated enzyme activity, demonstrated that the mitochondrial caspase cascade was activated to trigger apoptosis. Complex **2** consistently induced stronger activation than **L1**, indicating that metal coordination promotes signal transduction within this pathway. Molecular docking analysis further supported the experimental findings. Compared with **L1**, complex **2** exhibited lower binding energies toward CASP3 and CASP9 and established more favorable hydrogen-bonding and electrostatic interactions within their active sites. These structural characteristics provide a reasonable explanation for its enhanced biological activity and stronger proapoptotic effect.

Taken together, the findings suggest that **L1** and complex **2** inhibit HepG-2 cell proliferation through coordinated regulation of cell cycle arrest and mitochondria-dependent apoptosis. Rh(III) coordination consistently enhances the biological activity of the parent ligand, leading to improved antiproliferative efficacy and apoptotic induction. These results highlight the value of transition metal coordination in optimizing the pharmacological properties of emodin derivatives and provide useful evidence for the rational development of natural-product-based metal anticancer agents.

## 4. Materials and Methods

All commercially available chemicals and reagents were used directly as received, except where noted otherwise. Trypsin (Beijing Solarbio Technology Co., Ltd., Beijing, China), DMEM medium (Hyclone, Logan, UT, USA), fetal bovine serum (Gibco, Grand Island, NJ, USA), and the MTT and PI staining kits (Sigma, St. Louis, MO, USA) constituted the principal cell culture and viability detection reagents. The following items were procured from Beyotime (Shanghai, China): Fluo-3 AM (5 mM), JC-1 staining solution, the Annexin V-FITC apoptosis assay kit, DCFH-DA for reactive oxygen species detection, and the Caspase-3/9 activity assay kits. Analytical-grade reagents from Xilong Chemical Co., Ltd. (Shantou, China) were used for all other experimental needs.

A Bruker 400 MHz spectrometer was employed for NMR measurements. Crystallographic data were collected on an Agilent SuperNova diffractometer (Santa Clara, CA, USA) fitted with an EOS detector. Mass spectrometric analysis at ultra-high resolution was carried out using a Thermo Fisher Scientific instrument (Waltham, MA, USA). Cell manipulations were performed in a Hanbeak HB-402V ultra-clean workbench (Bucheon-si, Republic of Korea), and cellular images were captured with an Olympus IX51 inverted microscope (Tokyo, Japan). A Tecan microplate reader was utilized for absorbance-based assays. Flow cytometry experiments were run on an Attune NxT Acoustic Focusing Cytometer (Thermo Scientific, Waltham, MA, USA). Centrifugation steps were conducted with both a refrigerated high-speed centrifuge and a standard benchtop model, both manufactured by Sigma (St. Louis, MO, USA).

All flow cytometry data, including cell cycle, apoptosis, JC-1, Ca^2+^, ROS, and Caspase-3/9 assays, were analyzed using FlowJo V10. Western blot band intensities were quantified with ImageJ 1.8.0, and MTT assay data were processed using IBM SPSS Statistics 32.

### 4.1. Ligand Synthesis

A total of 2.00 g of emodin and 6.138 g of potassium carbonate were used as the starting materials and dissolved in acetone; then, 4.2 mL of dimethyl sulfate was added dropwise at 65 °C and was allowed to react for 18 h. After extraction and column chromatography purification (dichloromethane/methanol = 200:1), intermediate II was obtained (2.16 g, with a yield of 88.3%). A total of 1.30 g of intermediate II was dissolved in CCl_4_, and 0.742 g of NBS and a catalytic amount of BPO were added dropwise at 82 °C, followed by reflux for 20 h. Separation via column chromatography (180:1) afforded intermediate III (1.06 g, with a yield of 65.3%). 

Subsequently, 0.20 g of intermediate III, 0.088 g of either 5-amino-2,2′-bipyridine or 5-amino-1,10-phenanthroline, and 0.07 g of KI in dichloromethane were mixed, followed by reflux at 70 °C for 12 h. After the reaction was complete, the reaction mixture was filtered, and the filtrate was evaporated to dryness under reduced pressure to obtain a saturated reaction mother liquor. The mother liquor was placed in an Erlenmeyer flask containing 50 mL of petroleum ether and allowed to stand overnight to precipitate. It was then filtered to obtain a yellow solid. This was followed by purification via column chromatography to obtain the ligand, which was then dried under high vacuum for 24 h.


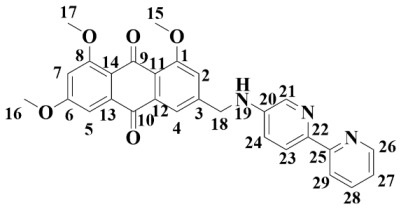


**Data for 3-(([2,2′-bipyridin]-5-ylamino)methyl)-1,6,8-trimethoxyanthracene-9,10-dione (L1):** The yellow color product of **L1** was suitable for structural characterization. The yield was 45.3%. ^1^H NMR (400 MHz, DMSO-*d*_6_) δ 8.52 (s, 1H, H-29), 8.25–8.12 (m, 2H, H-23, 26), 8.10 (s, 2H, H-21, 28), 7.79 (s, 1H, H-4), 7.53 (d, J = 9.0 Hz, 2H, H-2, 27), 7.25 (s, 2H, H-5, 24), 7.06 (s, 2H, H-7, 19), 4.51 (s, 2H, H-18), 4.01–3.87 (m, 9H, H-15, 16, 17). ^13^C NMR (101 MHz, DMSO-*d*_6_) δ 184.34 (C-10), 180.00 (C-9), 160.58 (C-6), 160.19 (C-8), 158.42 (C-1), 149.28 (C-25), 149.12 (C-26), 147.25 (C-3), 145.30 (C-22), 137.23 (C-20), 136.20 (C-28), 135.18 (C-21), 133.70 (C-13), 122.72 (C-12), 122.36 (C-24), 121.44 (C-23), 119.33 (C-29), 119.26 (C-27), 118.98 (C-11), 117.28 (C-4), 117.09 (C-14), 116.70 (C-2), 102.62 (C-5), 101.16 (C-7), 57.70 (C-15), 57.18 (C-16), 56.77 (C-17), 46.17 (C-18). HRMS (*m*/*z*): calcd for C_28_H_25_N_3_O_5_ [M + H]^+^: 482.1710; found: 482.1711. IR (KBr, cm^−1^): *V*_max_ 3725, 3428, 2923, 1602, 1417, 1323, 1246, 1120, 740.


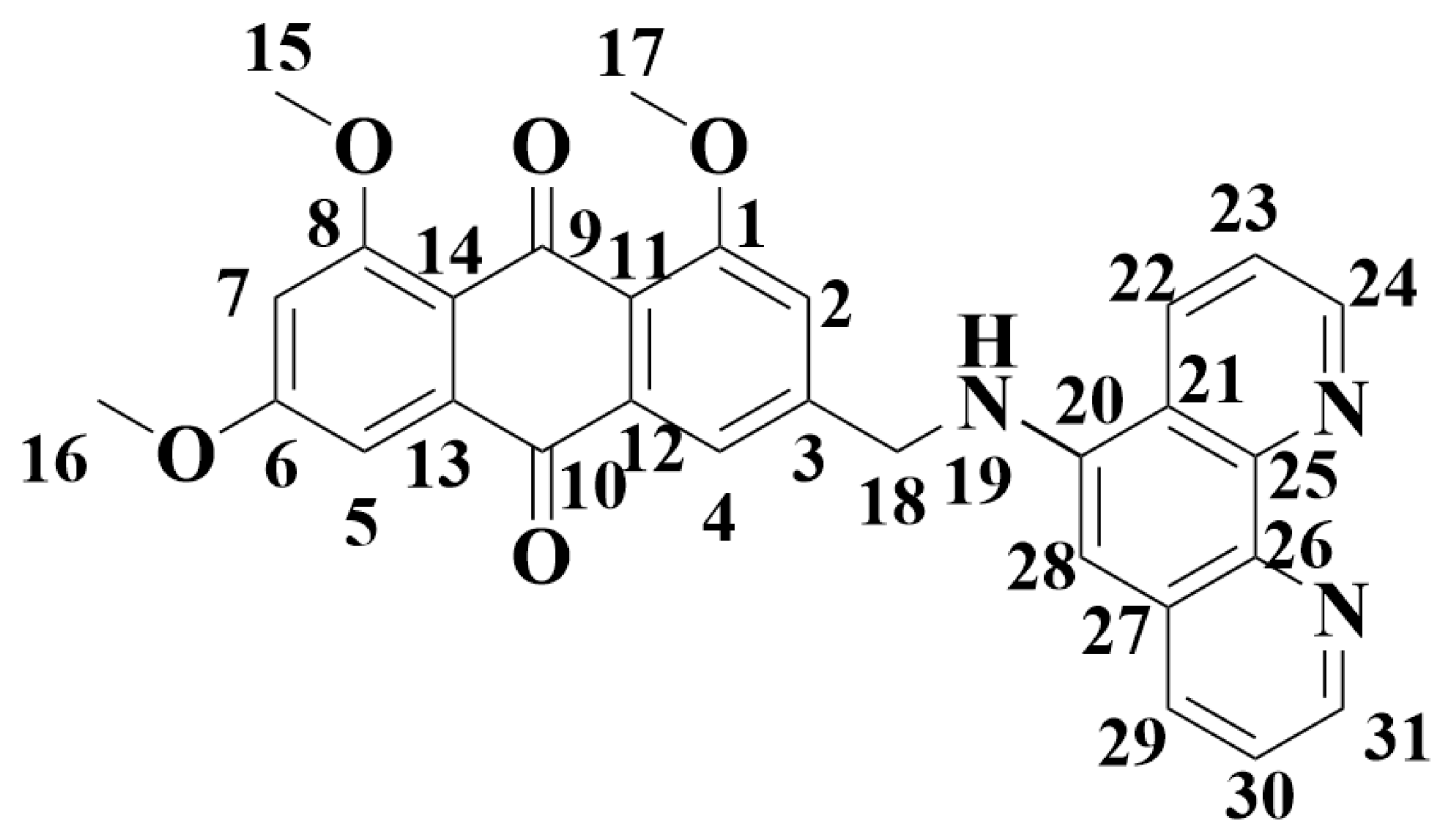


**Data for 3-(((1,10-phenanthrolin-5-yl)amino)methyl)-1,6,8-trimethoxyanthracen e-9,10-dione (L2):** The yellow color product of **L2** was suitable for structural characterization. The yield was 48.6%. ^1^H NMR (400 MHz, DMSO-*d*_6_) δ 8.52 (d, J = 3.7 Hz, 1H, H-31), 8.15 (d, J = 7.8 Hz, 1H, H-24), 8.10 (d, J = 8.3 Hz, 2H, H-29, 22), 7.78 (t, J = 7.5 Hz, 1H, H-30), 7.54 (d, J = 12.2 Hz, 2H, H-23, 4), 7.26–7.22 (m, 1H, H-19), 7.12–7.07 (m, 2H, H-2, 5), 7.06 (s, 2H, H-7, 28), 4.51 (s, 2H, H-18), 4.00 (s, 3H, H-15), 3.95 (s, 3H, H-17), 3.89 (s, 3H, H-16). ^13^C NMR (101 MHz, DMSO-*d*_6_) δ 183.81 (C-10), 179.47 (C-9), 160.10 (C-6), 159.71 (C-8), 157.95 (C-1), 148.78 (C-25), 146.77 (C-31), 146.67 (C-24), 144.77 (C-20), 144.65 (C-3), 136.72 (C-29), 136.70 (C-13), 135.71 (C-12), 134.70 (C-22), 133.21 (C-27), 122.14 (C-26), 121.89 (C-28), 120.93 (C-20), 120.90 (C-30), 118.73 (C-4), 118.55 (C-23), 116.64 (C-11), 116.24 (C-14), 116.20 (C-2), 102.18 (C-5), 100.69 (C-7), 57.19 (C-15), 56.69 (C-16), 56.28 (C-17), 45.71 (C-18). HRMS (*m*/*z*): calcd for C_30_H_24_N_3_O_5_ [M-H]^-^: 506.1710; IR (KBr, cm^−1^): *V*_max_ 3457, 2927, 1656, 1596, 1456, 1307, 1236, 742, 574.

### 4.2. Synthesis of Transition Metal Complexes

Each mixture of ligand **L1 or L2** (0.50 mmol) and a metal precursor (Co(NO_3_)_2_·6H_2_O, RhCl_3_·3H_2_O, Ru(DMSO)_4_Cl_2_, or Pt(DMSO)_2_Cl_2_, 0.50 mmol) was refluxed in 20 mL of methanol at 70 °C for 8 h and was then filtered to obtain the corresponding complex; complexes **1**–**7** were obtained in yields ranging from 30.3% to 47.6%. Different combinations of ligands and metals produced products with various forms, including red powders, red-brown crystals, or yellow powders.


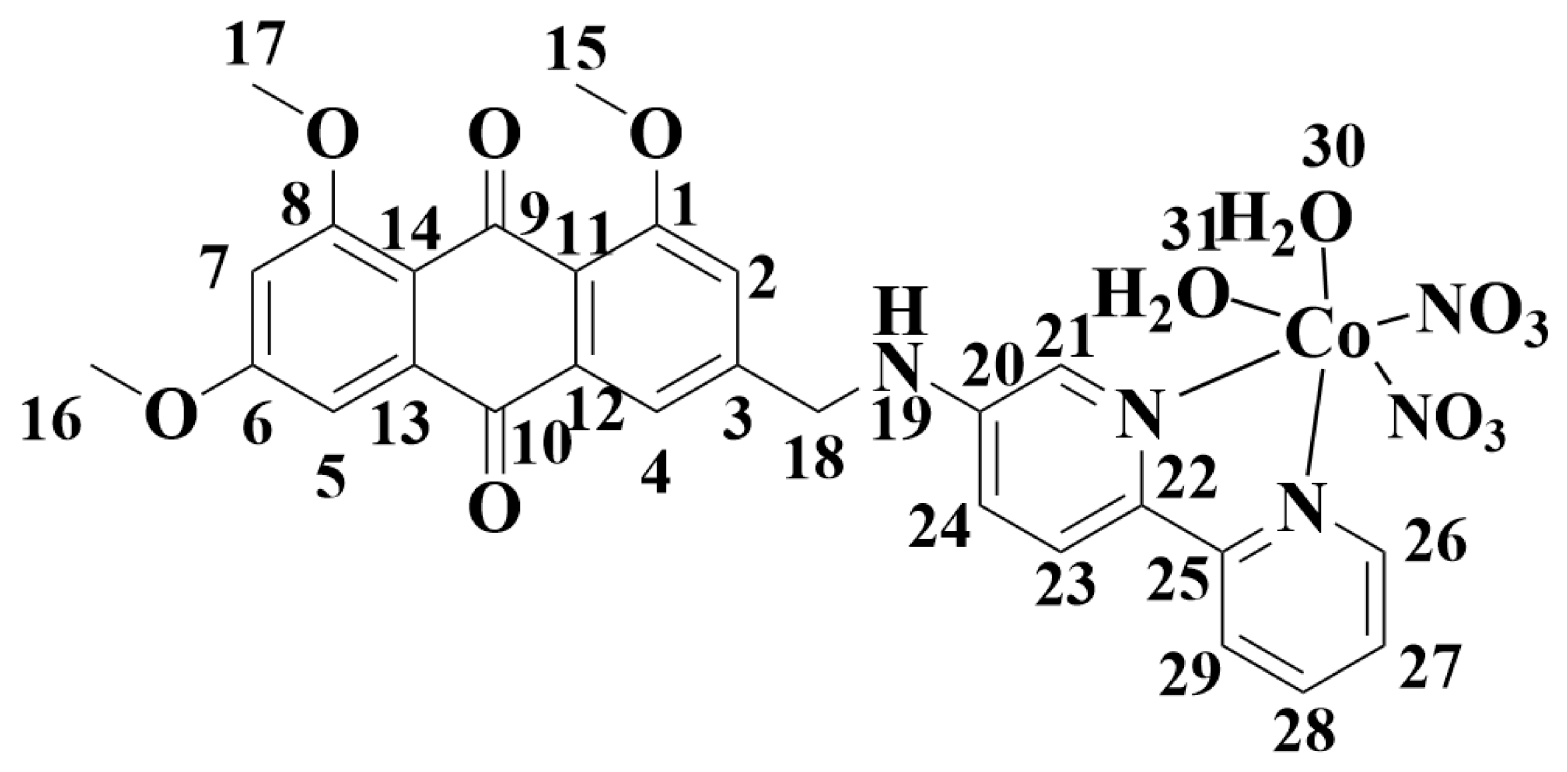


**Data for [Co(L1)(NO_3_)_2_(H_2_O)_2_] (1):** Red powder, the yield was 30.3%. ^1^H NMR (400 MHz, DMSO-*d*_6_) δ 8.15 (dd, J = 38.0, 13.7 Hz, 1H, H-23), 7.96 (s, 1H, H-29), 7.73–7.70 (m, 1H, H-26), 7.69–7.65 (m, 1H, H-21), 7.42–7.35 (m, 3H, H-28, 5, 27), 7.27 (d, J = 2.5 Hz, 1H, H-24), 7.24 (d, J = 2.5 Hz, 1H, H-7), 7.01 (dd, J = 55.3, 21.5 Hz, 2H, H-4, 2), 5.32 (t, J = 4.8 Hz, 1H, H-19), 4.22 (t, J = 6.5 Hz, 2H, H-18), 4.02–3.85 (m, 9H, H-15, 16, 17), 3.57 (d, J = 4.7 Hz, 4H, H-30, 31). ^13^C NMR (126 MHz, DMSO-*d*_6_) δ 183.18(C-10), 179.78(C-9), 163.36(C-6), 161.05(C-8), 159.11(C-1), 148.52(C-25), 148.25(C-26), 145.18(C-3), 143.34(C-22), 142.23(C-20), 136.16(C-28), 135.46(C-21), 134.05(C-13), 132.23(C-12), 125.61(C-24), 124.82(C-23), 123.87(C-29), 122.31(C-27), 117.97(C-11), 117.39(C-4), 116.59(C-14), 104.93(C-2), 102.30(C-5), 98.42(C-7), 56.42(C-15), 56.33(C-16), 55.86(C-17), 46.54(C-18). Anal. Calcd for C_30_H_27_CoN_5_O_13_: C, 49.32; H, 4.55; N, 9.29%; found: C, 49.36; H, 4.47; N, 9.32%. HRMS (*m*/*z*): calcd for [Co(L1)(NO_3_)_2_(H_2_O)_2_-H_2_O]: 682.0832; found: 682.0837. IR (KBr, cm^−1^): *V*_max_ 3407, 1596, 1384, 1384, 1285, 1100, 1022, 955, 852, 700, 600.


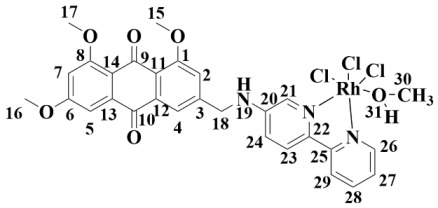


**Data for [Rh(L1)Cl_3_CH_3_OH] (2)**: Red-brown crystals, the yield was 39.6%. ^1^H NMR (400 MHz, DMSO-*d*_6_) δ 9.55 (dd, J = 21.9, 5.3 Hz, 1H, H-23), 9.22 (d, J = 2.5 Hz, 1H, H-29), 8.34 (d, J = 9.1 Hz, 1H, H-26), 8.30–8.24 (m, 1H, H-21), 8.14–8.02 (m, 1H, H-28), 7.71–7.65 (m, 1H, H-27), 7.63–7.44 (m, 2H, H-7, 24), 7.40–7.27 (m, 1H, H-4), 7.10 (dd, J = 4.7, 2.5 Hz, 1H, H-2), 6.92 (d, J = 2.3 Hz, 1H, H-5), 4.55 (dd, J = 27.9, 6.0 Hz, 2H, H-18), 3.87 (dd, J = 11.4, 8.4 Hz, 9H, H-15, 16, 17), 3.54 (s, 3H, H-30), 3.53 (s, 1H, H-31). 13C NMR (101 MHz, DMSO-*d*_6_) δ 183.85 (C-10), 180.48 (C-9), 164.05 (C-6), 161.70 (C-8), 159.74 (C-1), 158.43 (C-25), 157.77 (C-26), 156.62 (C-3), 151.93 (C-22), 147.37 (C-20), 145.53 (C-28), 142.20 (C-21), 140.51 (C-13), 136.20 (C-12), 134.82 (C-24), 125.54 (C-23), 124.98 (C-29), 122.99 (C-27), 122.22 (C-11), 118.70 (C-4), 118.11 (C-14), 117.38 (C-2), 105.65 (C-5), 102.95 (C-7), 57.08 (C-15), 56.99 (C-16), 56.54 (C-17), 46.29 (C-18), 43.61 (C-30). Anal. Calcd for C_29_H_27_Cl_3_N_3_O_6_Rh: C, 48.19; H, 3.77; N, 5.81%; found: C, 48.16; H, 3.83; N, 5.80%. HRMS (*m*/*z*): calcd for [Rh(L1)Cl_3_CH_3_OH-Cl+H_2_O-2H]^-^: 702.0281; found: 702.0287. IR (KBr, cm^−1^): *V*_max_ 3729, 3440, 2917, 1658, 1598, 1463, 1319, 1247, 1122, 1016, 950, 805, 602.


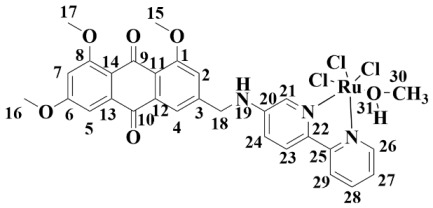


**Data for [Ru(L1)Cl_3_CH_3_OH] (3)**: Yellow powder, the yield was 40.2%. ^1^H NMR (400 MHz, DMSO-*d*_6_) δ 9.51 (d, J = 5.1 Hz, 1H, H-23), 9.40 (d, J = 5.8 Hz, 1H, H-29), 9.18 (d, J = 2.6 Hz, 1H, H-26), 8.96 (s, 1H, H-21), 8.27–8.19 (m, 1H, H-28), 8.02 (s, 1H, H-5), 7.90 (s, 1H, H-27), 7.68 (dd, J = 3.7, 1.0 Hz, 1H, H-24), 7.57 (s, 1H, H-7), 7.50 (d, J = 6.4 Hz, 1H, H-4), 7.13 (d, J = 2.4 Hz, 1H, H-2), 6.96 (t, J = 1.9 Hz, 1H, H-19), 5.30 (s, 1H, H-31), 4.49 (d, J = 6.3 Hz, 2H, H-18), 3.90 (dd, J = 5.3, 1.3 Hz, 9H, H-15, 16, 17), 3.87 (s, 3H, H-30).^13^C NMR (101 MHz, DMSO-*d*_6_) δ 183.20 (C-10), 179.86 (C-9), 163.42 (C-6), 161.08 (C-8), 159.10 (C-1), 154.94 (C-25), 145.73 (C-26), 145.37 (C-3), 143.73 (C-22), 136.95 (C-20), 135.53 (C-28), 134.08 (C-21), 123.89 (C-13), 123.19 (C-12), 122.25 (C-24), 122.21 (C-23), 120.73 (C-29), 118.08 (C-27), 117.47 (C-11), 117.40 (C-4),116.85 (C-14), 116.48 (C-2), 105.02 (C-5), 102.34 (C-7), 56.40 (C-15), 55.94 (C-16), 45.75 (C-17), 44.12 (C-18), 42.67 (C-30). Anal. Calcd for C_29_H_27_Cl_3_N_3_O_6_Ru: C, 48.31; H, 3.77; N, 5.81%; found: C, 48.02; H, 4.15; N, 5.96%. HRMS (*m*/*z*): calcd for [Ru(L1)Cl_3_CH_3_OH-Cl+H_2_O-H]^-^: 702.0348; found: 702.0355. IR (KBr, cm^−1^): *V*_max_ 3729, 3440, 2927, 1656, 1380, 1253, 1157.


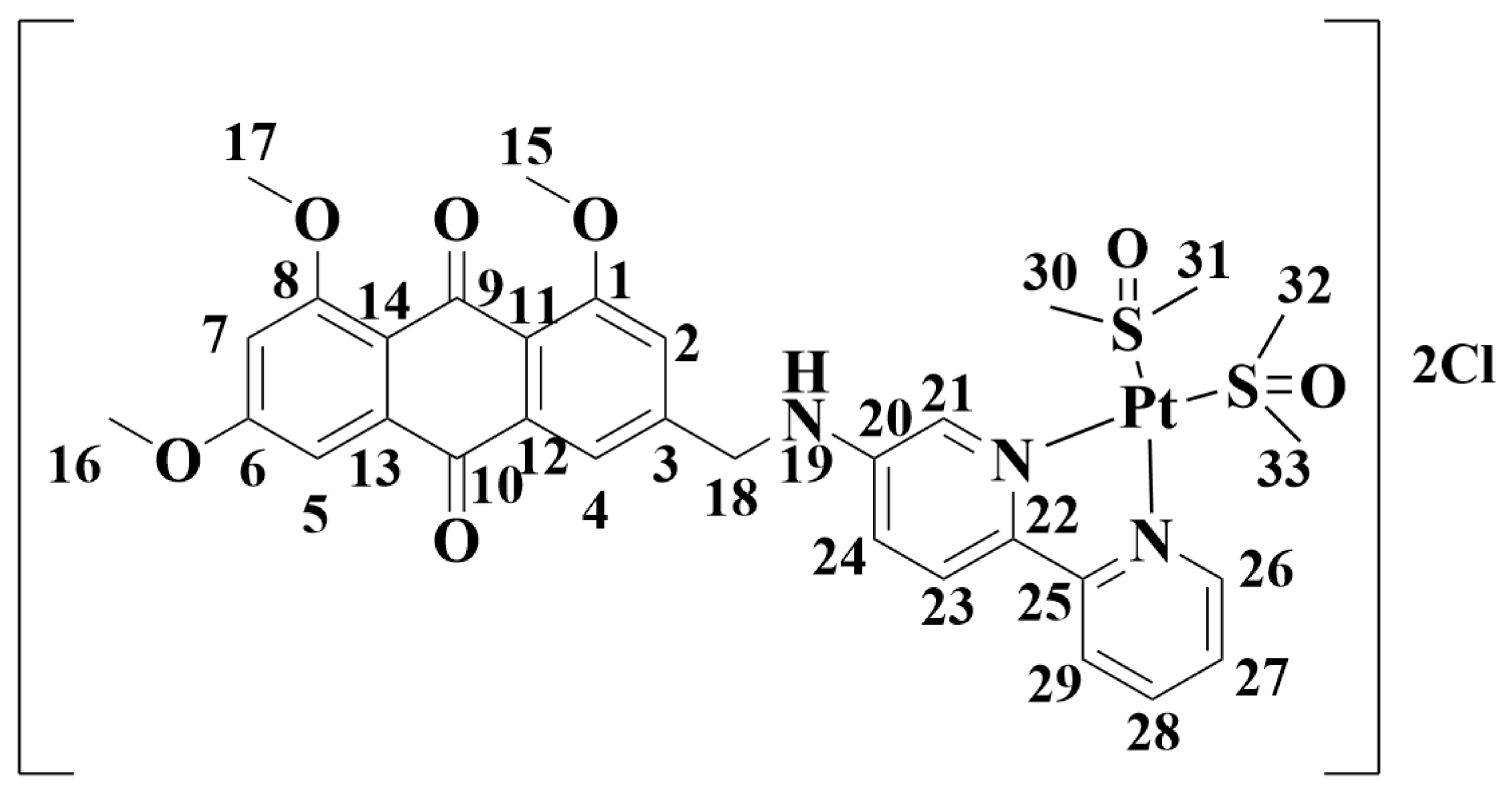


**Data for [Pt(L1)(DMSO)_2_] Cl_2_(4)**: Yellow powder, the yield was 42.7%. ^1^H NMR (400 MHz, DMSO-*d*_6_) δ 9.28 (d, J = 5.8 Hz, 1H, H-23), 8.95 (d, J = 2.3 Hz, 1H, H-29), 8.20–8.07 (m, 4H, H-5, 21, 26, 28), 7.67 (d, J = 1.1 Hz, 1H, H-27), 7.57–7.53 (m, 2H, H-7, 24), 7.40 (dd, J = 9.0, 2.5 Hz, 1H, H-4), 7.12 (d, J = 2.4 Hz, 1H, H-2), 6.94 (d, J = 2.4 Hz, 1H, H-19), 4.57 (d, J = 6.0 Hz, 2H, H-18), 3.96–3.86 (m, 9H, H-15, 16, 17), 2.54 (s, 12H, H-30, 31, 32, 33). ^13^C NMR (101 MHz, DMSO-*d*_6_) δ 183.16 (C-10), 179.80 (C-9), 163.41 (C-6), 161.07 (C-8), 159.08 (C-1), 157.80 (C-25), 147.42 (C-26), 147.13 (C-3), 144.83 (C-22), 142.98 (C-20), 139.89 (C-28), 135.50 (C-21), 134.14 (C-13), 125.07 (C-12), 124.51 (C-24), 122.33 (C-23), 122.30 (C-29), 121.31 (C-27), 118.10 (C-11), 117.45 (C-4), 117.60 (C-14), 116.60 (C-2), 105.01 (C-5), 102.30 (C-7), 56.38 (C-15), 56.38 (C-16), 55.90 (C-17), 45.57 (C-18). Anal. Calcd for C_32_H_35_N_3_O_7_PtS_2_: C, 46.15; H, 4.24; N, 5.05%; found: C, 46.12; H, 4.15; N, 5.12%. HRMS (*m*/*z*): calcd for [Pt(L1)(DMSO)_2_]: 832.1562; found: 832.1561. IR (KBr, cm^−1^): *V*_max_ 3720, 3442, 2913, 1660, 1596, 1317, 1245, 1118, 1010, 572.


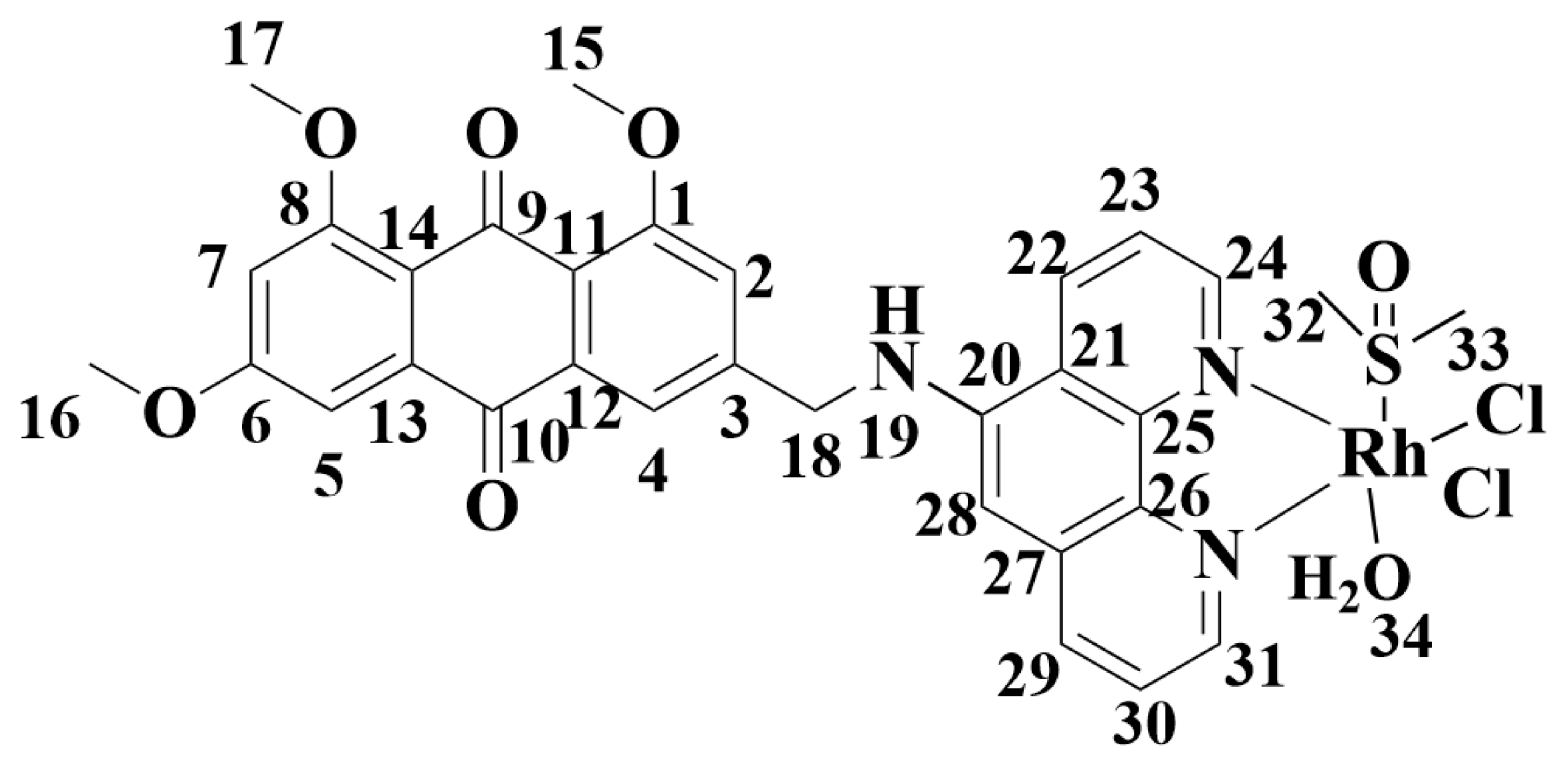


**Data for [Rh(L2)Cl_2_(DMSO)H_2_O]** (**5**): Brownish-red powder, the yield was 43.6%. ^1^H NMR (400 MHz, DMSO-*d*_6_) δ 10.08 (d, J = 5.4 Hz, 1H, H-31), 9.48 (d, J = 5.0 Hz, 1H, H-24), 9.27 (d, J = 8.5 Hz, 1H, H-29), 8.49 (d, J = 8.2 Hz, 1H, H-22), 8.26 (dd, J = 8.3, 5.6 Hz, 1H, H-30), 8.15 (d, J = 5.7 Hz, 1H, H-23), 7.97 (dd, J = 8.2, 5.3 Hz, 1H, H-5), 7.78 (s, 1H, H-28), 7.72 (s, 1H, H-7), 7.11 (d, J = 2.1 Hz, 1H, H-4), 6.99 (s, 1H, H-7), 6.95 (d, J = 1.8 Hz, 1H, H-19), 4.79 (d, J = 5.1 Hz, 2H, H-18), 3.95–3.86 (m, 9H, H-15, 16, 17), 3.65 (s, 6H, H-32, 33), 2.54 (s, 1H, H-34). ^13^C NMR (126 MHz, DMSO-*d*_6_) δ 184.22 (C-10), 180.83 (C-9), 164.35 (C-6), 162.04 (C-8), 160.10 (C-1), 146.34 (C-25), 146.30 (C-31), 144.30 (C-24), 141.63 (C-20), 137.14 (C-3), 136.54 (C-29), 135.13 (C-13), 135.02 (C-12), 133.63 (C-22), 127.11 (C-27), 127.00 (C-26), 123.33 (C-28), 119.16 (C-20), 118.45 (C-30), 118.42 (C-4), 117.55 (C-23), 105.94 (C-11), 105.82 (C-14), 103.34 (C-2), 103.32 (C-5), 99.56 (C-7), 57.44 (C-15), 57.42 (C-16), 57.32 (C-17), 56.83 (C-18), 47.55 (C-32), 41.39 (C-33). Anal. Calcd for C_32_H_34_Cl_2_N_3_O_7_RhS: C, 50.14, H, 4.34; N, 5.32%; found: C, 50.21; H, 4.25; N, 5.31%. HRMS (*m*/*z*): calcd for [Rh(L2)Cl_2_(DMSO)H_2_O-H_2_O-DMSO]:834.0348; found: 834.0343. IR (KBr, cm^−1^): *V*_max_ 3415, 1654, 1594, 1455, 1321, 1243, 1004, 831, 715, 610.


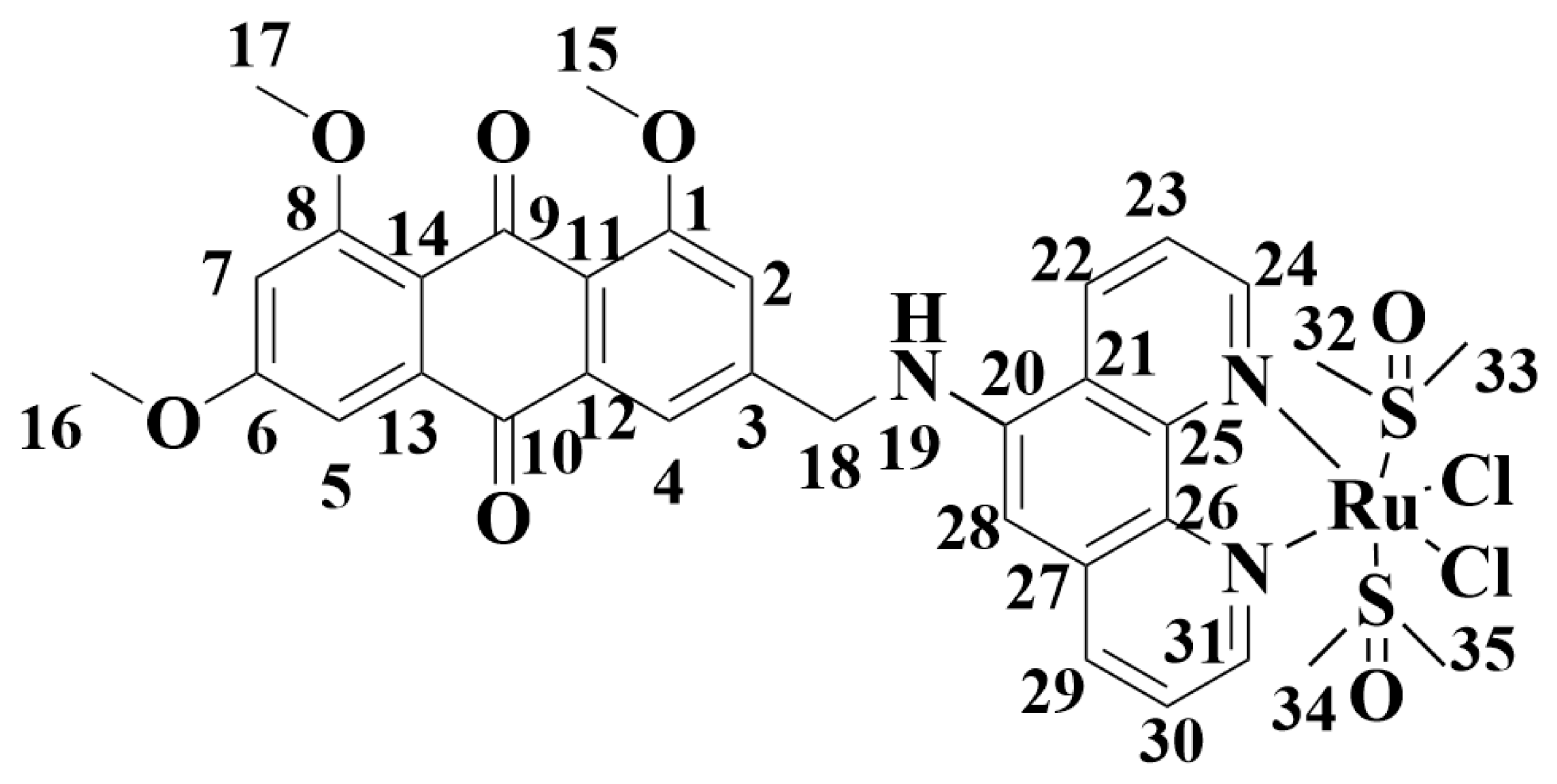


**Data for [Ru(L2)Cl_2_(DMSO)_2_]** (**6**): Yellow powder, the yield was 40.9%. ^1^H NMR (400 MHz, DMSO-*d*_6_) δ 9.81 (d, J = 4.9 Hz, 1H, H-31), 9.46 (d, J = 4.8 Hz, 1H, H-24), 9.19 (d, J = 8.4 Hz, 1H, H-29), 8.17 (d, J = 8.0 Hz, 1H, H-22), 8.12 (dd, J = 8.4, 5.3 Hz, 1H, H-30), 8.07 (t, J = 5.8 Hz, 1H, H-23), 7.77 (s, 1H, H-5), 7.71 (s, 1H, H-28), 7.62 (dd, J = 8.2, 5.3 Hz, 1H, H-7), 7.06 (d, J = 2.3 Hz, 1H, H-4), 6.90 (d, J = 2.3 Hz, 1H, H-2), 6.82 (s, 1H, H-19), 4.71 (d, J = 5.5 Hz, 2H, H-18), 3.94–3.80 (m, 9H, H-15, 16, 17), 3.30 (s, 12H, H-32, 33, 34, 35). ^13^C NMR (101 MHz, DMSO-*d*_6_) δ 183.91 (C-10), 180.54 (C-9), 164.00 (C-6), 161.67 (C-8), 159.72 (C-1), 148.28 (C-25), 146.47 (C-31), 146.35 (C-24), 143.86 (C-20), 136.22 (C-3), 134.73 (C-29), 132.63 (C-13),132.42 (C-12), 124.45 (C-22), 123.78 (C-27), 123.66 (C-26), 122.93 (C-28), 118.80 (C-20), 118.09 (C-30), 118.02 (C-4), 117.23 (C-23), 105.57 (C-11), 105.55 (C-14), 102.97 (C-2), 102.86 (C-5), 99.17 (C-7), 57.09 (C-15), 56.99 (C-16), 56.52 (C-17), 46.69 (C-18), 46.00 (C-32), 45.94 (C-33), 44.88 (C-34), 43.74 (C-35). Anal. Calcd for C_34_H_35_Cl_2_N_3_O_7_RuS_2_: C, 48.98; H, 4.23; N, 5.04%; found: C, 48.75; H, 4.50; N, 5.15%. HRMS (*m*/*z*): calcd for [Ru(L2)Cl_2_(DMSO)_2_+H]^+^: 834.0410; found: 834.0412. IR (KBr, cm^−1^): *V*_max_ 3725, 3442, 2923, 2262, 1600, 1428, 1317, 1247, 1101, 1010, 813.


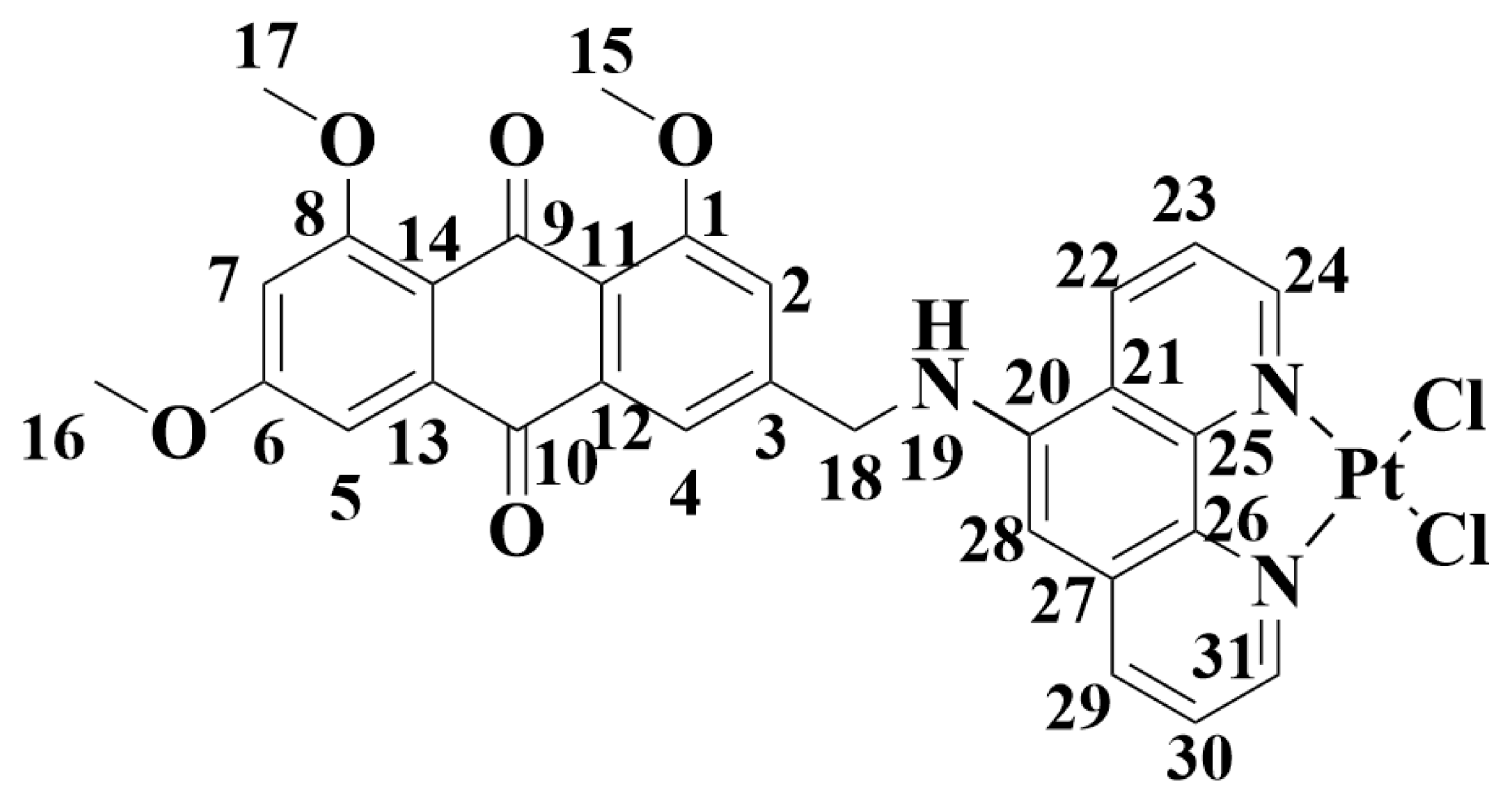


**Data for [Pt(L2)Cl_2_] (7)**: Yellow powder, the yield was 47.6%. ^1^H NMR (400 MHz, DMSO-*d*_6_) δ 9.60 (d, J = 5.2 Hz, 1H, H-31), 9.24 (d, J = 8.6 Hz, 1H, H-24), 9.09(d, J = 5.0 Hz, 1H, H-29), 8.36 (d, J = 8.3 Hz, 1H, H-22), 8.12–8.03 (m, 2H, H-23, 30), 7.71 (d, J = 13.7 Hz, 2H, H-2, 4), 7.62 (s, 1H, H-5), 7.00 (s, 1H, H-7), 6.87 (d, J = 8.4 Hz, 1H, H-19), 6.75 (s, 1H, H-28), 4.69 (d, J = 4.8 Hz, 2H, H-18), 3.94–3.78 (m, 9H, H-15, 16, 17). ^13^C NMR (126 MHz, DMSO-*d*_6_) δ 183.99 (C-10), 180.58 (C-9), 164.16 (C-6), 161.86 (C-8), 159.92 (C-1), 149.51 (C-25), 149.05 (C-31), 145.99 (C-24), 144.51 (C-20), 144.15 (C-3), 142.23 (C-29), 136.97 (C-13), 136.27 (C-12), 134.93 (C-22), 134.86 (C-27), 133.04 (C-26), 126.42 (C-28), 125.62 (C-20), 124.67 (C-30), 123.11 (C-4), 118.78 (C-23), 118.20 (C-11), 117.40 (C-14), 105.73 (C-2), 103.10 (C-5), 99.22 (C-7), 57.23 (C-15), 57.14 (C-16), 56.66 (C-17), 47.34 (C-18). Anal. Calcd for C_30_H_23_Cl_2_N_3_O_5_Pt: C, 46.70; H, 3.01; N, 5.45%; found: C, 46.66; H, 3.13; N, 5.36%. HRMS (*m*/*z*): calcd for [Pt(L2)Cl_2_-2Cl+2H_2_O+Na-H]^-^: 758.1311; found: 758.1313. IR (KBr, cm^−1^): *V*_max_ 3729, 3397, 1650, 1596, 1527, 1423, 1328.

### 4.3. Cell Culture

Seven human cancer cell lines, namely, 293T (CRL-3216), CAL-27 (CRL-2095), MCF-7 (HTB-22), HepG-2 (HB-8065), A549 (CCL-185), SW-480 (CCL-228), and T-24 (HTB-4), were obtained from the American Type Culture Collection (ATCC, Manassas, VA, USA). Each cell line was maintained in the culture medium specifically recommended by ATCC. All media were supplemented with 10% fetal bovine serum (FBS) and 1% penicillin–streptomycin solution. Cells were cultured at 37 °C in a humidified incubator supplied with 5% CO_2_, with the exception of SW-480 cells, which were grown under a 100% air atmosphere in the absence of CO_2_.

### 4.4. Cell Viability Assay

Cal-27, MCF-7, HepG-2, A549, SW-480, and T-24 cells in the logarithmic growth phase were collected for the assay. After trypsinization and centrifugation, the cells were resuspended in complete medium and seeded into 96-well plates at 5 × 10^3^–8 × 10^3^ cells per well, and the cell cultures were maintained at 37 °C under 5% CO_2_. Drug treatment was initiated once 40–60% confluence was reached. Preliminary screening was conducted at 20 μM, and concentration gradients of 0, 1, 5, 10, 15, and 20 μM were applied with five replicate wells each for IC_50_ determination. After 48 h exposure, the cells were rinsed twice with PBS and incubated with 200 μL MTT solution for 4–6 h. The medium was then aspirated, 150 μL of DMSO was added, and the OD values were recorded on a microplate reader following 10 min of shaking. All experiments were performed in triplicate, and cell inhibition rates and IC_50_ values were subsequently calculated.

### 4.5. Cell Cycle Detection by Flow Cytometry

Cells in the logarithmic growth phase were harvested, trypsinized, and resuspended in complete medium after centrifugation. The cells were seeded into 6-well plates at 2.5 × 10^5^ cells per well and maintained at 37 °C under 5% CO_2_. Once 50–60% confluence was reached, **L1** and **2** were applied at final concentrations of 0, 4, and 8 μM. After treatment, the cells were collected by trypsinization, rinsed twice with PBS, and fixed overnight at −20 °C in cold 70% ethanol. The fixed cells were pelleted at 2000 rpm for 10 min, washed with ice-cold PBS, and then incubated with 100 μL of RNase A at 37 °C for 30 min. PI staining solution (400 μL) was subsequently added, and the samples were kept in the dark at 4 °C for 30 min, after which red fluorescence was detected at 488 nm by flow cytometry.

### 4.6. Detection of Apoptosis by Flow Cytometry

Cell seeding was carried out as described in Section 4.5. After treatment, the cells were harvested by trypsinization and rinsed twice with PBS. The resulting pellet was resuspended in 500 μL of Binding Buffer, after which Annexin V-FITC (5 μL) and propidium iodide (5 μL) were added and thoroughly mixed. The samples were kept in the dark at room temperature for 5–15 min, and apoptotic cells were subsequently analyzed by flow cytometry.

### 4.7. Detection of Mitochondrial Membrane Potential by Flow Cytometry

Cell seeding was carried out as described in Section 4.5. After treatment, the cells were harvested by trypsinization and rinsed twice with PBS. JC-1 working solution (500 μL) was added, followed by gentle mixing, and incubation was conducted at 37 °C under 5% CO_2_ for 30 min. The cells were subsequently pelleted by centrifugation, with the supernatant being discarded, and the pellet was hed twice with JC-1 staining buffer. The cells were then resuspended and stained with 500 μL of the same buffer, after which flow cytometric analysis was completed within 30 min.

### 4.8. Detection of Intracellular ROS by Flow Cytometry

Cell seeding and drug treatment were carried out as described in Section 4.5. After treatment, the cells were harvested by trypsinization and rinsed twice with PBS. DCFH-DA probe solution (300 μL) was added, followed by gentle mixing, and incubation was conducted at 37 °C in the dark for 30 min. The cells were subsequently pelleted by centrifugation, with the supernatant being discarded, and the pellet was rinsed three times with PBS (5 min each time). The pellet was finally resuspended in 500 μL of culture medium and analyzed by flow cytometry.

### 4.9. Flow Cytometry Detection of Intracellular Ca^2+^Levels

Cell seeding and drug treatment were carried out as described in Section 4.5. After treatment, the cells were harvested by trypsinization and rinsed twice with PBS. Fluo-4 AM staining solution (5 μM, 500 μL) was added, and incubation was conducted at 37 °C in the dark for 30 min. The staining solution was subsequently removed, and the cells were washed twice with pre-cooled staining buffer (1000 rpm, 5 min) to eliminate the residual probe. The pellet was then resuspended in 500 μL of calcium-free HBSS buffer and incubated at 37 °C in the dark for 20 min to ensure the complete hydrolysis of the AM groups. Intracellular Ca^2+^ levels were finally determined via flow cytometry.

### 4.10. Flow Cytometry Detection of Caspase-3 Activity

Cell seeding and drug treatment were carried out as described in Section 4.5. After treatment, the cells were harvested by trypsinization and rinsed twice with PBS. The pellet was resuspended in 200 μL of PBS, followed by the addition of 1 μL of Caspase-3/7 substrate (Green) and gentle mixing. Incubation was then conducted at 37 °C in the dark for 30 min. PBS (1 mL) was subsequently added, and centrifugation was performed at 1000 rpm for 5 min. The supernatant was discarded, the pellet was resuspended in 500 μL of PBS, and caspase-3 activity was finally determined via flow cytometry.

### 4.11. Flow Cytometry Detection of Caspase-9 Activity

Cell seeding and drug treatment were carried out as described in Section 4.5. After treatment, the cells were harvested by trypsinization and rinsed twice with PBS. The pellet was resuspended in 400 μL of PBS, followed by the addition of 0.4 μL of Caspase-9 substrate (Green) to afford a final concentration of 1 μM. The suspension was gently mixed, and incubation was conducted at 37 °C in the dark for 30 min. PBS (1 mL) was subsequently added, and centrifugation was performed at 1000 rpm for 5 min. The supernatant was discarded, the pellet was resuspended in 500 μL of PBS, and caspase-9 activity was finally determined via flow cytometry.

### 4.12. Molecular Docking and Visualization Processing

Molecular docking was conducted using AutoDock Vina 1.2.3, and the docking outputs were subsequently visualized and analyzed with PyMol 2.5.5 and Ligplot v2.2.9.

### 4.13. Graphical Abstract Design

The graphical abstract was designed with the assistance of the AI tool Doubao Version 14.2.0 (ByteDance, Beijing, China).

## 5. Conclusions

In this study, emodin was used as the parent scaffold to successfully prepare ligands **L1** and **L2**, along with seven transition metal complexes. The single-crystal structure of Rh(III) complex **2** was confirmed to adopt a six-coordinate distorted octahedral geometry. In vitro evaluation showed that **L1** and complex **2** exhibited significant inhibitory effects against HepG-2 cells, with better safety profiles than cisplatin. Mechanistic studies revealed that both compounds induced G2/M phase arrest through Cyclin B1 downregulation and P21 upregulation. Meanwhile, ROS and Ca^2+^ accumulation was promoted, mitochondrial membrane potential was disrupted, and the Caspase-9/3 cascade was activated, thereby initiating the intrinsic apoptotic pathway. Rh(III) coordination significantly enhanced the activity of **L1**. Complex **2** exhibited superior performance to **L1** in all evaluated aspects, and molecular docking analysis further confirmed its stronger binding affinity toward Caspase-3 and Caspase-9. Collectively, the results show that complex **2** has the characteristics of an efficient, low-toxicity, and mechanistically defined anticancer lead compound. This study provides experimental evidence for further optimization of natural product-based metal drugs.

## Figures and Tables

**Scheme 2:**
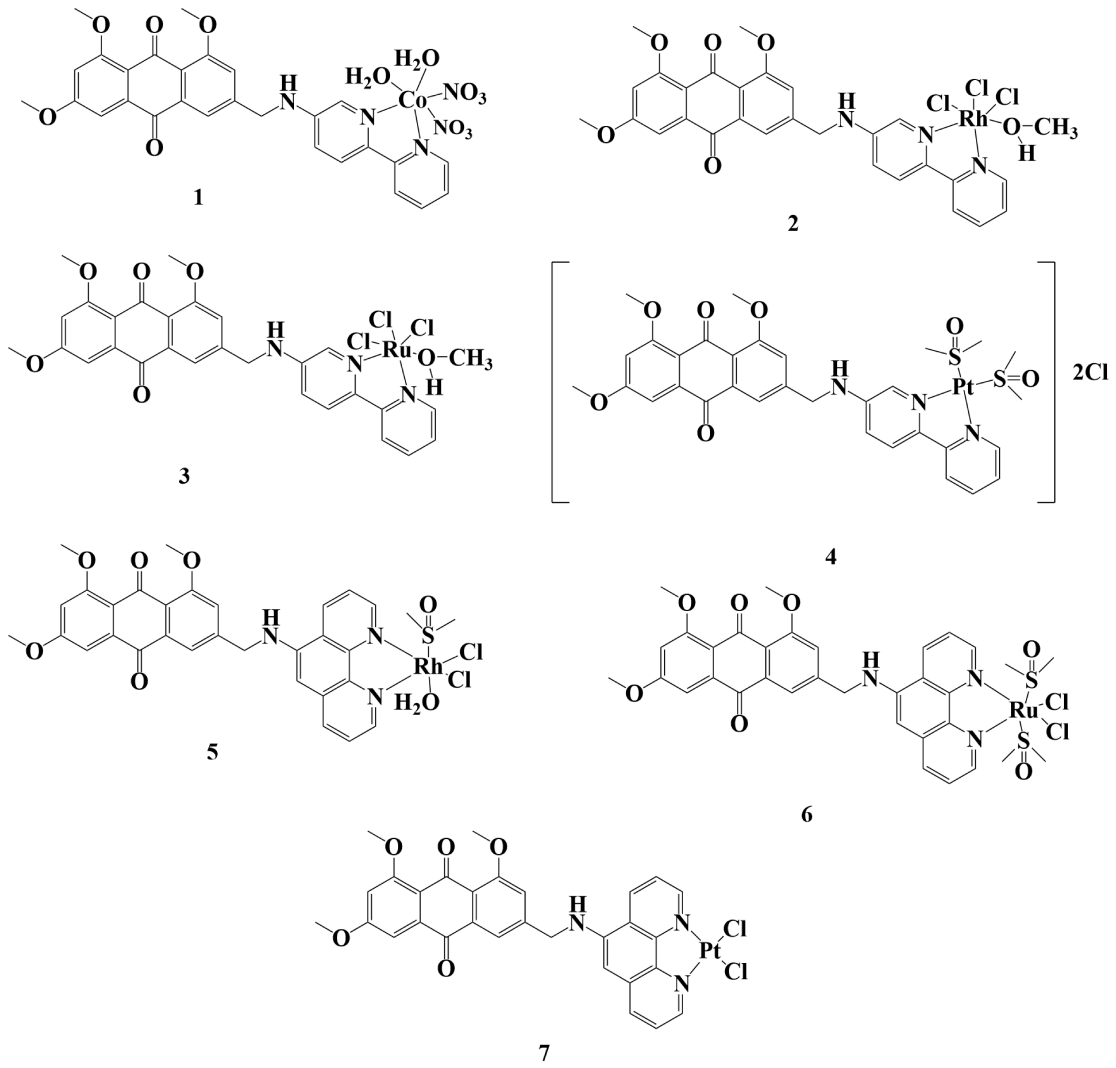
Chemical structures of complexes **1**–**7.**

**Table 1:** IC_50_ (µM) values of **L1**, **2**, **7** and cisplatin towards the selected five cell lines after an incubation for 48 h.

Compound	IC_50_ μM					SF
	HepG—2	SW—480	MCF—7	Cal—27	293T	
**L1**	8.77 ± 0.08	17.68 ± 2.89	13.98 ± 4.44	21.67 ± 1.69	51.69 ± 0.01	5.89
**2**	6.10 ± 0.06	36.11 ± 2.86	19.27 ± 0.65	11.43 ± 4.42	40.20 ± 1.16	6.59
**7**	21.14 ± 0.73	28.92 ± 1.22	19.03 ± 1.54	>50	15.72 ± 0.06	0.74
Cisplatin	9.05 ± 0.40	8.03 ±1.35	12.06 ± 2.63	19.06 ± 1.06	14.52 ± 1.21	1.60

Note: SF (Selectivity factor) = IC_50_(293T)/IC_50_(HepG—2). The IC_50_ value is the average of three independent experiments ± standard deviation.

**Table 2:** Binding Energies of Ligands **L1** and Complexes **2** to Target Protein Molecules.

Target Protein	Compound	Docking_Score (kcal/mol)
1NW9-CASP9	**2**	−7.618
1NW9-CASP9	**L1**	−7.340
7RN9-CASP3	**2**	−7.334
7RN9-CASP3	**L1**	−6.932

Note: A negative binding affinity value indicates the possibility of binding, and the smaller the value, the greater the binding potential.

## Data Availability

Data are contained within the article and Supplementary Materials.

## Supplementary Materials

The following supporting information can be downloaded at: https://www.mdpi.com/article/10.3390/molecules31152682/s1: Table S1. Crystallographic parameters of complex **2**; Table S2–S4. Selected bond lengths [Å] and bond angles [°] of complex **2**; Table S5. Inhibition rates (%) of the two ligands and seven transition metal complexes in different tumor cell lines; Figures S1–S3. HRMS, ^1^H NMR, and ^13^C NMR spectra of **L1**; Figures S4–S6. HRMS, ^1^H NMR, and ^13^C NMR spectra of **L2**; Figures S7–S9. HRMS, ^1^H NMR, and ^13^C NMR spectra of complex **1**; Figures S10–S12. HRMS, ^1^H NMR, and ^13^C NMR spectra of complex **2**; Figures S13–S15. HRMS, ^1^H NMR, and ^13^C NMR spectra of complex **3**; Figures S16–S18. HRMS, ^1^H NMR, and ^13^C NMR spectra of complex **4**; Figures S19–S21. HRMS, ^1^H NMR, and ^13^C NMR spectra of complex **5**; Figures S22–S24. HRMS, ^1^H NMR, and ^13^C NMR spectra of complex **6**; Figures S25–S27. HRMS, ^1^H NMR, and ^13^C NMR spectra of complex **7**; Figures S28–S47: UV–Vis absorption spectra of **L1, L2,** and complexes **1–7** and those at different time intervals. Figures S48–S56: The HPLC chromatograms of **L1, L2,** and complexes **1–7**. Figures S57–S66: IR spectra of **L1, L2,** and complexes **1–7**.
