# Supplementary material for: Synthesis and Antitumor Mechanism of Emodin-Derived Transition Metal Complexes
Source: Molecules. 2026 Jul 31;31(15):2682. doi: 10.3390/molecules31152682 (PMC13468083; doi:10.3390/molecules31152682)
Supplement: Supplementary file 1 [file molecules-31-02682-s001.zip › molecules-4404493-supplementary.pdf]

Synthesis and Antitumor Mechanism of Emodin-derived Transition Metal Complexes

Yumin Pan [1,†], Ying Rui [1,†], Biqun Zou [1,*] , Xiaoteng Jing[1], Ruijie He[2,*], Jianyi Liang[1,3] and Fangyao Li[3,*]

1 School of Chemistry and Life Sciences, Guilin Normal University, Guilin 541199, China; panyumin@mail.glnc.edu.cn (Y.P.); ruiying@mail.glnc.edu.cn (Y.R.); jingxiaoteng@mail.glnc.edu.cn (X.J.); 18897730565@163.com (J.L.)

2 Guangxi Key Laboratory of Functional Phytochemicals and Continuous Utilization of Resources,
Guangxi Institute of Botany, Guangxi Zhuang Autonomous Region and Chinese Academy of Sciences,
Guilin 541006, China

3 College of Pharmacy, Guilin Medical University, Guilin 541100, China

* Correspondence: zoubiqun@mail.glnc.edu.cn (B.Z.); hrj937@gxib.cn (R.H.); lifangyao@glmu.edu.cn (F.L.)

† These authors contributed equally to this work.

Table S1 Crystallographic parameters of complex **2**

Empirical formula	$C_{29}H_{27}Cl_3N_3O_6Rh$
Formula weight	722.79
Temperature/K	100.00(10)
Crystal system	orthorhombic
Space group	Pbca
a/Å	30.2560(3)
b/Å	24.6270(3)
c/Å	9.90130(10)
α/°	90
β/°	90
γ/°	90
Volume/Å^3	7377.60(14)
Z	8
ρ_{calc}g/cm^3	1.301
μ/mm^{-1}	6.071

F(000)	2928.0
Crystal size/mm^3	0.13 × 0.1 × 0.09
Radiation	Cu Kα (λ = 1.54184)
2Θ range for data collection/°	10.062 to 133.18
Index ranges	-36 ≤ h ≤ 26, -28 ≤ k ≤ 29, -11 ≤ l ≤ 7
Reflections collected	27404
Independent reflections	6318 [R_{int} = 0.0478, R_{sigma} = 0.0303]
Data/restraints/parameters	6318/1463/421
Goodness-of-fit on F^2	1.010
Final R indexes [I>=2σ (I)]	R_1 = 0.0997, wR_2 = 0.2431
Final R indexes [all data]	R_1 = 0.1071, wR_2 = 0.2493
Largest diff. peak/hole / e Å^{-3}	1.79/-1.34

Table S2 Selected Bond Lengths [Å] of Complex **2**

**Atom**	**Atom**	**Length/Å**	**Atom**	**Atom**	**Length/Å**
Rh1	Cl2	2.353(2)	C5	C28	1.514(17)
Rh1	Cl1	2.353(2)	C21	C23	1.464(11)
Rh1	Cl3	2.383(4)	C21	C00L	1.394(11)
Rh1	N2	2.032(6)	C20	C00L	1.370(10)
Rh1	N3	2.040(7)	C20	C19	1.394(12)
Rh1	O6	2.315(7)	C7	C6	1.397(11)
Rh1	Cl4	2.310(8)	C7	C2	1.388(11)
Rh1	O8	2.158(13)	C14	C13	1.390(11)
O5	C12	1.224(10)	C14	C15	1.334(12)
O2	C4	1.339(10)	C14	C18	1.530(10)
O2	C8	1.414(10)	C23	C24	1.365(12)
N2	C21	1.344(10)	C19	C22	1.401(11)
N2	C22	1.340(10)	C6	C12	1.497(11)
N3	C23	1.357(10)	C2	C3	1.394(12)
N3	C27	1.371(10)	C4	C3	1.375(12)
O1	C2	1.342(10)	C15	C16	1.407(11)
O1	C1	1.489(13)	C24	C25	1.395(11)
N1	C19	1.342(10)	C27	C26	1.390(13)

N1	C18	1.439(10)	C25	C26	1.360(13)
O4	C16	1.347(11)	C10	C16	1.398(12)
O4	C17	1.445(13)	C10	C9	1.511(14)
C11	C13	1.391(11)	C10	C28	1.499(16)
C11	C12	1.491(11)	C9	O3	1.210(14)
C11	C10	1.413(12)	O7	C28	1.196(17)
C5	C6	1.381(12)	O6	C29	1.362(7)
C5	C4	1.434(11)	O8	C1A	1.354(9)

Table S3 Selected Bond Angles [°] of Complex **2**

Atom	Atom	Atom	Angle/°	Atom	Atom	Atom	Angle/°
Cl00	Rh1	Cl	176.49(14)	C00G	C00K	N009	113.6(12)
Cl00	Rh1	Cl2	89.57(18)	C00G	C00K	C00V	127.2(13)
Cl	Rh1	Cl2	91.44(18)	C00V	C00K	N009	119.2(14)
N008	Rh1	Cl00	87.9(3)	C00H	C00L	C00G	120.7(12)
N008	Rh1	Cl	88.7(3)	N00B	C00M	C00H	124.6(13)
N008	Rh1	Cl2	93.1(4)	N00B	C00M	C00S	120.7(13)
N008	Rh1	O1	174.4(3)	C00S	C00M	C00H	114.7(13)
N009	Rh1	Cl00	89.6(4)	C00J	C00N	C00E	121.4(13)
N009	Rh1	Cl	89.0(4)	C00F	C00O	C00P	119.8(12)
N009	Rh1	Cl2	174.1(4)	C00I	C00O	C00F	121.9(13)
N009	Rh1	N008	81.0(5)	C00I	C00O	C00P	118.2(12)
N009	Rh1	O1	104.5(4)	O006	C00P	C00E	121.3(13)
O1	Rh1	Cl00	92.66(18)	O006	C00P	C00O	119.6(13)
O1	Rh1	Cl	90.81(19)	C00E	C00P	C00O	119.0(12)
O1	Rh1	Cl2	81.3(2)	O00A	C00Q	C00T	112.8(14)
C00R	O007	C013	115.3(12)	C00I	C00Q	O00A	124.7(14)
C00G	N008	Rh1	111.4(9)	C00I	C00Q	C00T	122.5(15)
C00S	N008	Rh1	125.6(10)	O007	C00R	C00F	117.2(13)
C00S	N008	C00G	122.7(12)	O007	C00R	C00T	121.6(13)
C00K	N009	Rh1	116.0(11)	C00T	C00R	C00F	121.2(13)
C00K	N009	C00W	118.4(13)	N008	C00S	C00M	124.7(14)
C00W	N009	Rh1	125.5(11)	C00R	C00T	C00Q	117.8(15)
C00Q	O00A	C014	115.0(14)	C011	C00U	C00J	123.1(16)
C00M	N00B	C00Z	121.6(12)	C00X	C00V	C00K	123.3(15)
C011	O00C	C018	116(2)	C010	C00W	N009	118.6(17)
C00N	C00E	C00P	119.8(12)	C00V	C00X	C010	117.2(16)
C00N	C00E	C00Y	119.4(13)	C00E	C00Y	C011	118.9(14)
C00Y	C00E	C00P	120.8(13)	C00E	C00Y	C012	122.7(15)

Atom	Atom	Atom	Angle/°	Atom	Atom	Atom	Angle/°
C00O	C00F	C012	120.4(15)	C011	C00Y	C012	118.2(14)
C00R	C00F	C00O	117.5(13)	N00B	C00Z	C00J	112.9(13)
C00R	C00F	C012	122.1(14)	C00W	C010	C00X	123.1(19)
C00K	C00G	N008	117.6(12)	O00C	C011	C00U	122.3(16)
C00K	C00G	C00L	127.6(13)	O00C	C011	C00Y	119.6(16)
C00L	C00G	N008	114.8(13)	C00U	C011	C00Y	118.1(14)
C00L	C00H	C00M	122.3(13)	C00Y	C012	C00F	116.9(16)
C00Q	C00I	C00O	118.8(13)	O6	C012	C00F	117.6(19)
C00N	C00J	C00U	118.8(14)	O6	C012	C00Y	122(2)
C00N	C00J	C00Z	122.6(13)	C6	O1	Rh1	110.7(10)
C00U	C00J	C00Z	118.4(14)				

Table S4 Selected Torsion Angles [°] of Complex **2**

A	B	C	D	Angle/°	A	B	C	D	Angle/°
Rh1	N008	C00G	C00K	3.4(17)	C00O	C00F	C00R	O007	179.1(13)
Rh1	N008	C00G	C00L	-176.1(10)	C00O	C00F	C00R	C00T	-2(2)
Rh1	N008	C00S	C00M	176.2(11)	C00O	C00F	C012	C00Y	-6(3)
Rh1	N009	C00K	C00G	6.7(18)	C00O	C00F	C012	O6	154(2)
Rh1	N009	C00K	C00V	-173.6(11)	C00O	C00I	C00Q	O00A	179.4(14)
Rh1	N009	C00W	C010	174.7(14)	C00O	C00I	C00Q	C00T	-3(2)
O007	C00R	C00T	C00Q	177.2(14)	C00P	C00E	C00N	C00J	179.9(14)
N008	C00G	C00K	N009	-7(2)	C00P	C00E	C00Y	C011	-176.9(14)
N008	C00G	C00K	C00V	173.8(14)	C00P	C00E	C00Y	C012	-1(2)
N008	C00G	C00L	C00H	2(2)	C00Q	C00I	C00O	C00F	-1(2)
N009	C00K	C00V	C00X	-5(2)	C00Q	C00I	C00O	C00P	-179.7(13)
N009	C00W	C010	C00X	2(3)	C00R	C00F	C00O	C00I	3(2)
O00A	C00Q	C00T	C00R	-178.2(14)	C00R	C00F	C00O	C00P	-177.5(12)
N00B	C00M	C00S	N008	176.8(14)	C00R	C00F	C012	C00Y	174.2(15)
C00E	C00Y	C011	O00C	175.3(16)	C00R	C00F	C012	O6	-26(3)
C00E	C00Y	C011	C00U	-6(2)	C00S	N008	C00G	C00K	177.8(14)
C00E	C00Y	C012	C00F	6(3)	C00S	N008	C00G	C00L	-2(2)
C00E	C00Y	C012	O6	-154(2)	C00U	C00J	C00N	C00E	0(2)
C00F	C00O	C00P	O006	-177.9(14)	C00U	C00J	C00Z	N00B	135.8(15)
C00F	C00O	C00P	C00E	1(2)	C00V	C00X	C010	C00W	-3(3)
C00F	C00R	C00T	C00Q	-1(2)	C00W	N009	C00K	C00G	-176.7(14)
C00G	N008	C00S	C00M	3(2)	C00W	N009	C00K	C00V	3(2)

A	B	C	D	Angle/°	A	B	C	D	Angle/°
C00G	C00K	C00V	C00X	174.8(17)	C00Y	C00E	C00N	C00J	0(2)
C00H	C00M	C00S	N008	-4(2)	C00Y	C00E	C00P	O006	177.1(15)
C00I	C00O	C00P	O006	1(2)	C00Y	C00E	C00P	C00O	-2(2)
C00I	C00O	C00P	C00E	-179.6(13)	C00Z	N00B	C00M	C00H	-9(2)
C00I	C00Q	C00T	C00R	4(3)	C00Z	N00B	C00M	C00S	170.7(14)
C00J	C00U	C011	O00C	-174.5(17)	C00Z	C00J	C00N	C00E	175.5(15)
C00J	C00U	C011	C00Y	7(3)	C00Z	C00J	C00U	C011	-179.6(15)
C00K	N009	C00W	C010	-1(2)	C011	C00Y	C012	C00F	-179.0(15)
C00K	C00G	C00L	C00H	-177.3(15)	C011	C00Y	C012	O6	22(3)
C00K	C00V	C00X	C010	5(3)	C012	C00F	C00O	C00I	-176.1(15)
C00L	C00G	C00K	N009	172.9(14)	C012	C00F	C00O	C00P	3(2)
C00L	C00G	C00K	C00V	-7(3)	C012	C00F	C00R	O007	-1(2)
C00L	C00H	C00M	N00B	-176.4(14)	C012	C00F	C00R	C00T	177.2(16)
C00L	C00H	C00M	C00S	4(2)	C012	C00Y	C011	O00C	0(3)
C00M	N00B	C00Z	C00J	-69.8(17)	C012	C00Y	C011	C00U	177.9(16)
C00M	C00H	C00L	C00G	-4(2)	C013	O007	C00R	C00F	-177.9(13)
C00N	C00E	C00P	O006	-3(2)	C013	O007	C00R	C00T	4(2)
C00N	C00E	C00P	C00O	178.1(13)	C014	O00A	C00Q	C00I	-11(2)
C00N	C00E	C00Y	C011	3(2)	C014	O00A	C00Q	C00T	171.8(15)
C00N	C00E	C00Y	C012	178.3(16)	C018	O00C	C011	C00U	-4(3)
C00N	C00J	C00U	C011	-4(3)	C018	O00C	C011	C00Y	175(2)
C00N	C00J	C00Z	N00B	-39(2)					

Table S5 Inhibition Rates (%) of Two Ligands and Seven Complexes on Different Tumor Cell Lines

**Compound**	SW - 480	MCF - 7	Cal - 27	T - 24	HepG - 2	A549	293T
**L1**	69.36 ± 0.72	52.81 ± 2.02	55.05 ± 1.99	53.98 ± 3.52	65.42 ± 0.63	51.40 ± 2.12	16.91 ± 0.59
**L2**	42.85 ± 0.78	24.85 ± 0.56	41.43 ± 1.68	51.58 ± 2.40	38.13 ± 1.03	49.12 ± 2.88	30.19 ± 1.67
**1**	50.89 ± 1.32	27.04 ± 3.09	37.92 ± 1.34	20.79 ± 1.57	10.37 ± 1.65	26.88 ± 1.25	56.23 ± 1.21
**2**	51.41 ± 1.56	48.17 ± 0.28	58.81 ± 1.04	32.59 ± 1.28	78.03 ± 0.80	25.41 ± 2.82	26.76 ± 2.14
**3**	37.99 ± 0.03	14.68 ± 2.30	47.45 ± 0.73	15.00 ± 3.70	21.47 ± 1.34	28.00 ± 0.41	40.13 ± 0.89
**4**	44.22 ± 2.08	12.10 ± 2.20	29.82 ± 1.56	17.09 ± 1.29	27.38 ± 0.97	37.17 ± 1.89	35.02 ± 0.56
**5**	30.16 ± 0.84	16.97 ± 2.45	43.79 ± 1.34	37.95 ± 0.56	35.75 ± 2.01	31.23 ± 1.79	65.15 ± 0.36
6	22.53 ± 0.11	22.03 ± 1.28	11.93 ± 1.23	19.23 ± 0.32	14.24 ± 1.86	33.72 ± 0.55	53.19 ± 1.87
**7**	34.06 ± 1.86	45.51 ± 1.19	20.04 ± 0.65	10.40 ± 0.84	50.15 ± 1.74	21.47 ± 2.93	62.83 ± 0.27

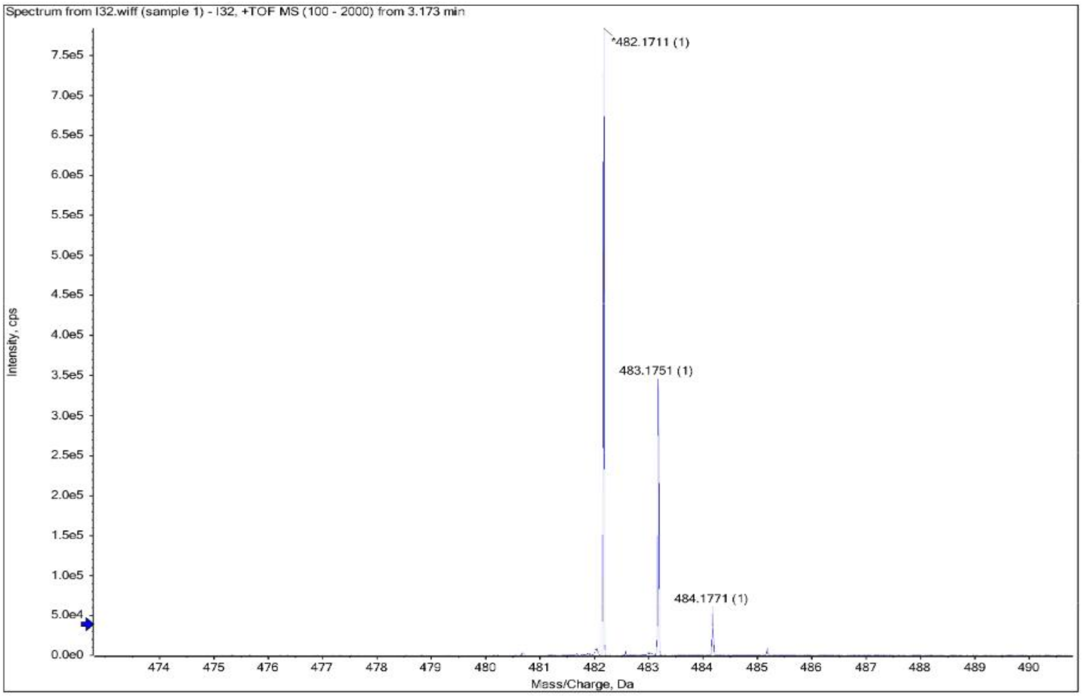

Figure S1 HRMS spectrum of **L1**

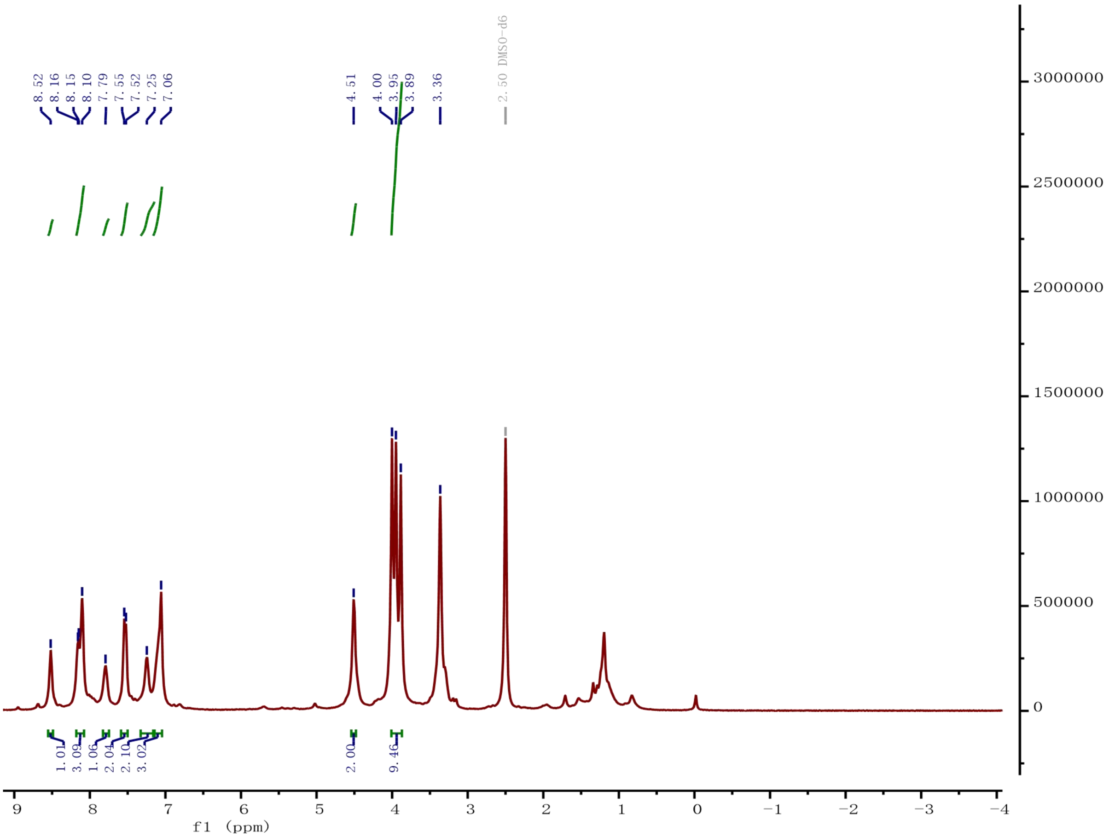

Figure S2 ^{1}H NMR spectrum of **L1**

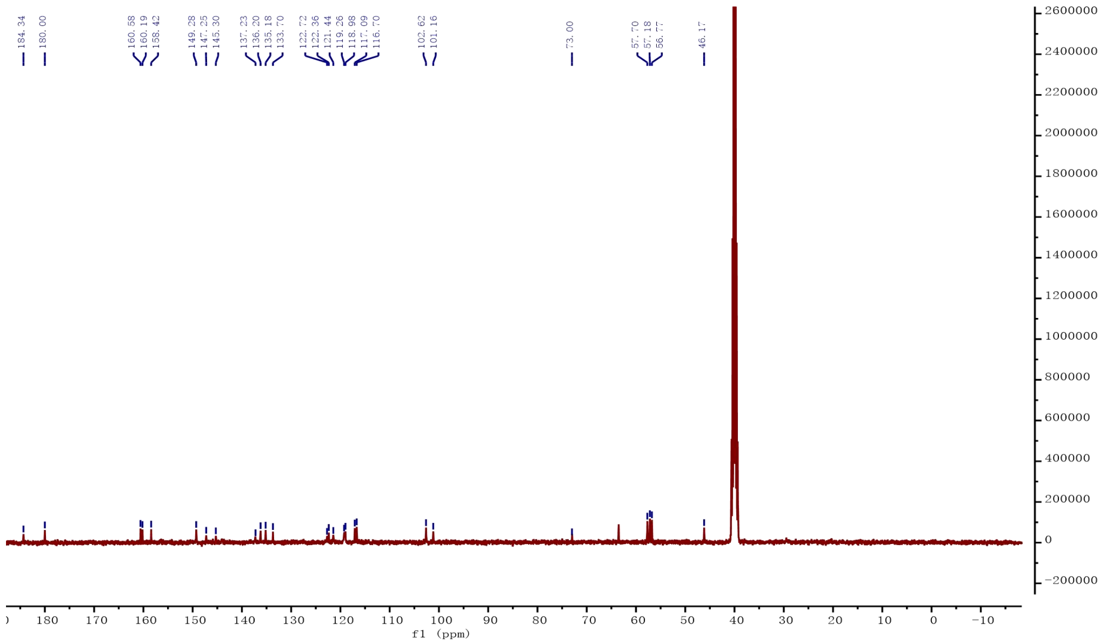

Figure S3 ^{13}C NMR spectrum of **L1**

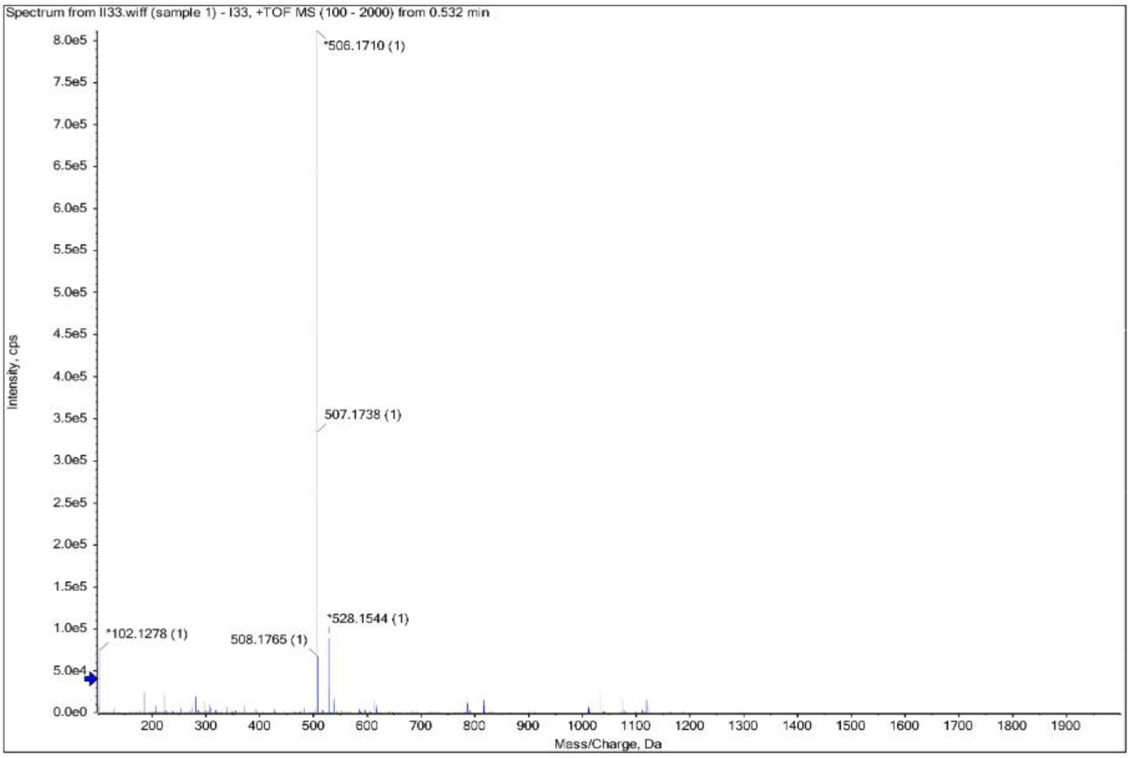

Figure S4 HRMS spectrum of **L2**

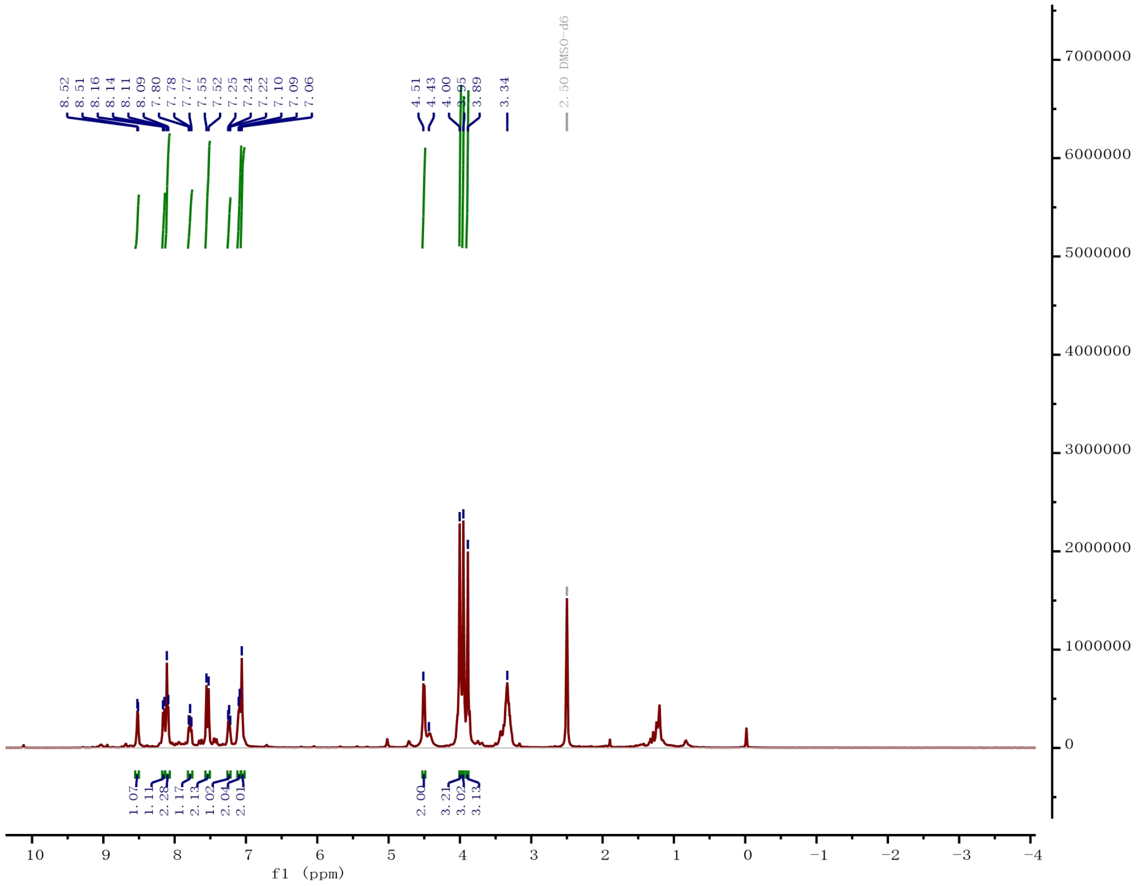

Figure S5 ^{1}H NMR spectrum of **L2**

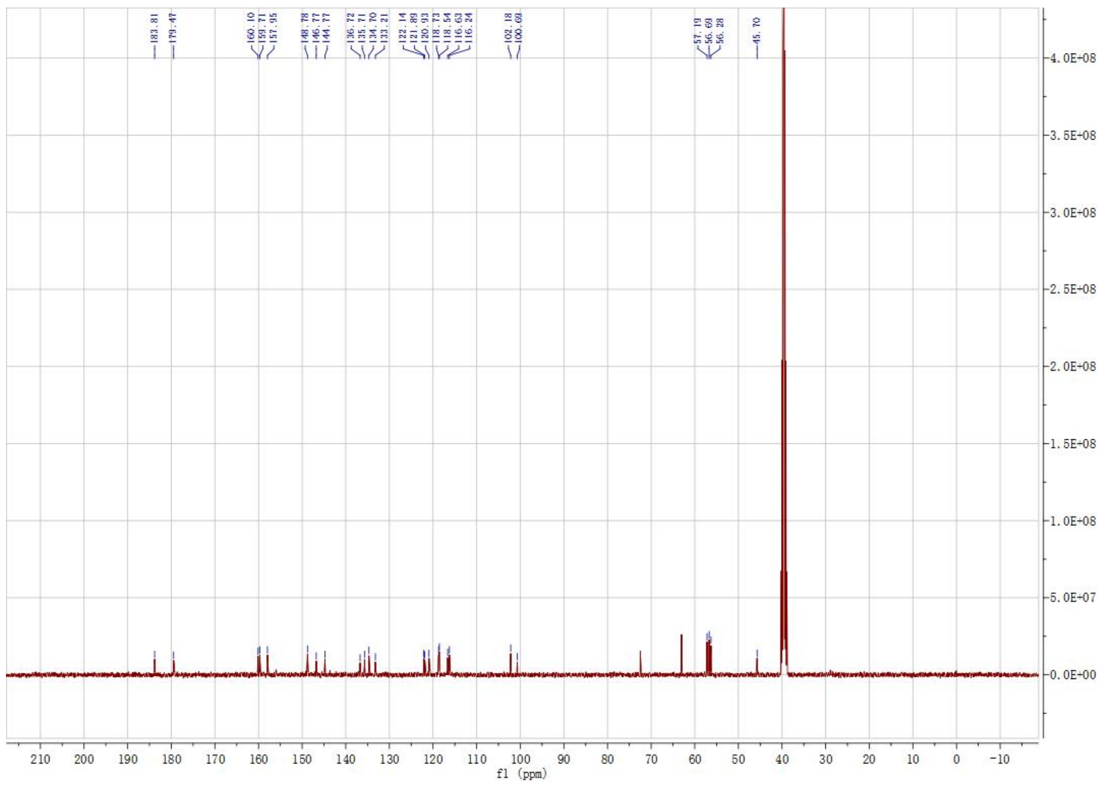

Figure S6 ^{13}C NMR spectrum of **L2**

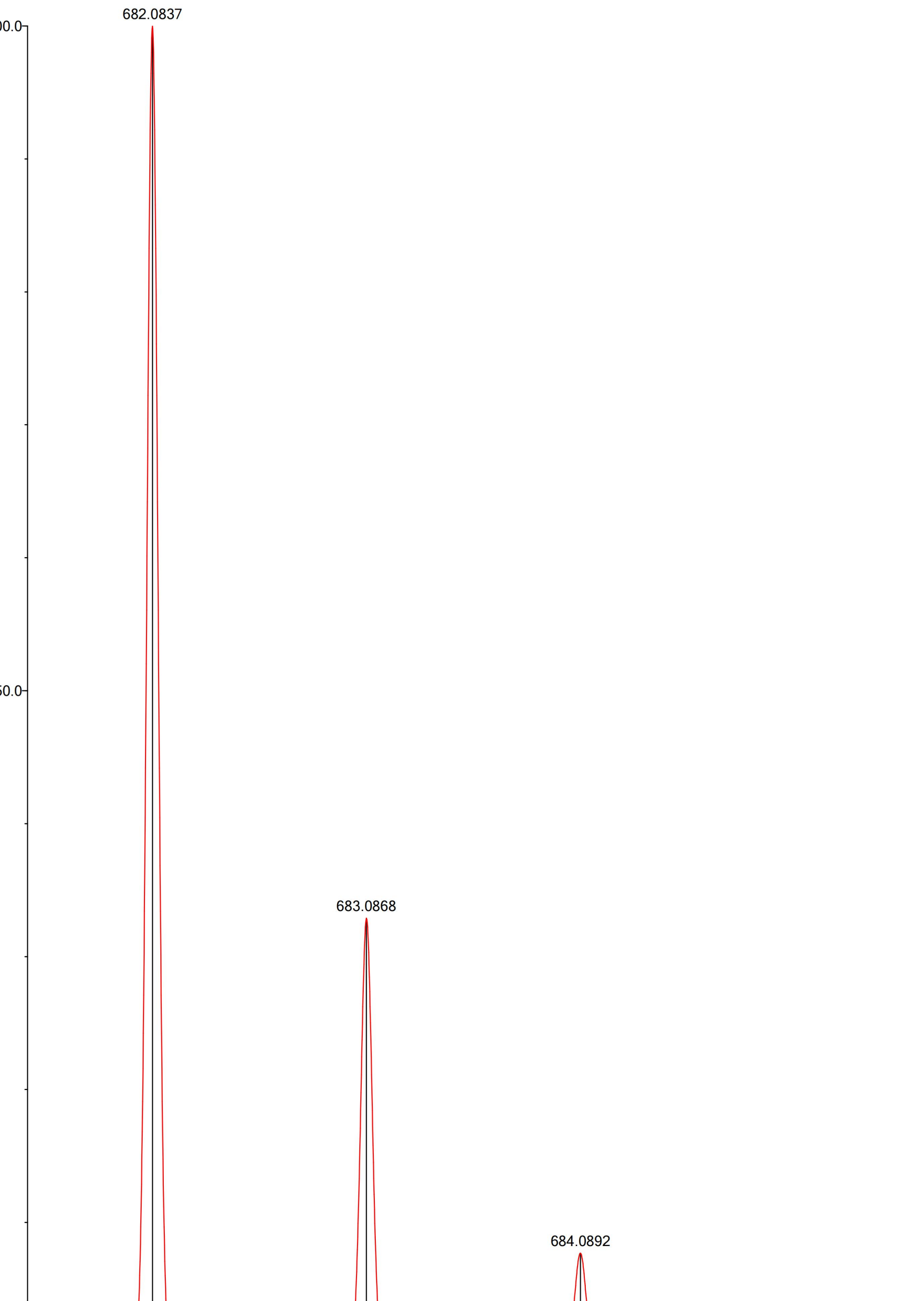

Figure S7 HRMS spectrum of **1**

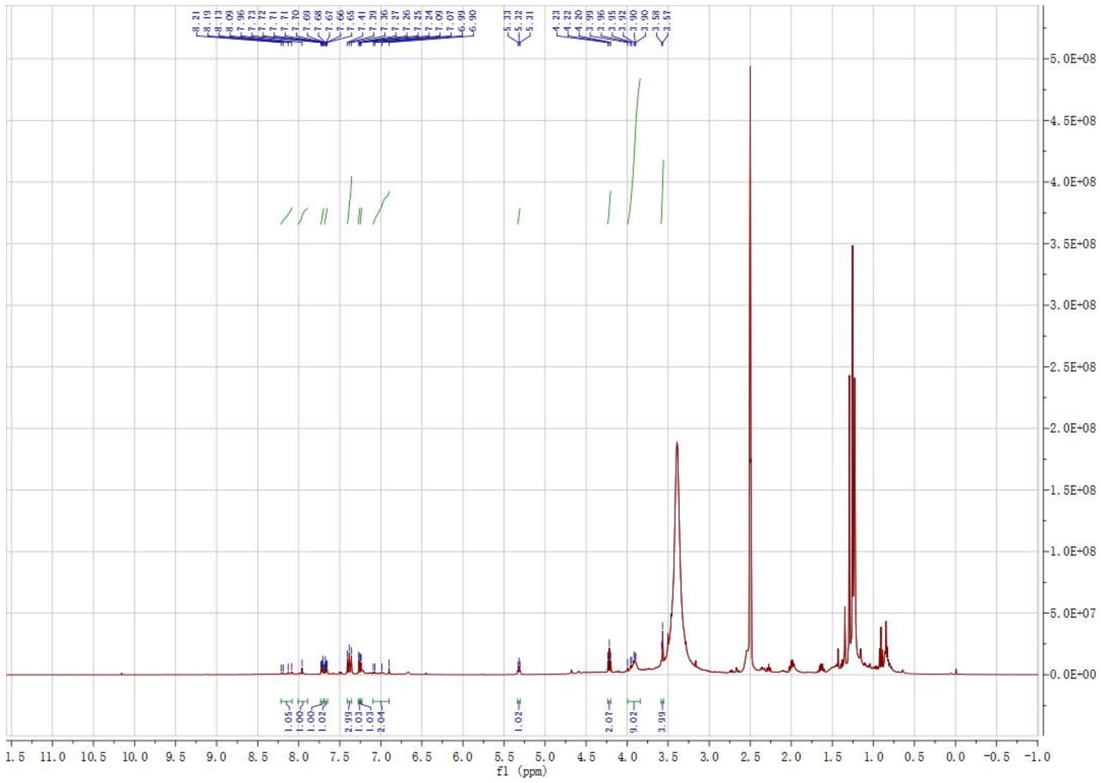

Figure S8 ^{1}H NMR spectrum of **1**

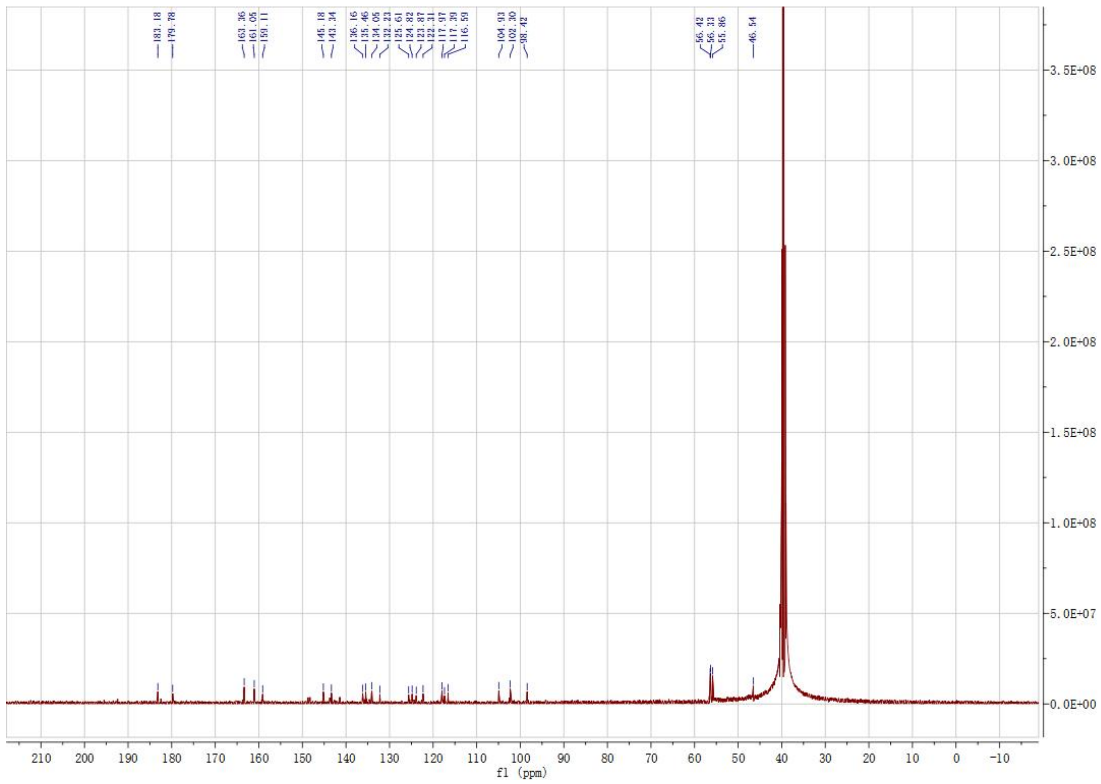

Figure S9 ^{13}C NMR spectrum of **1**

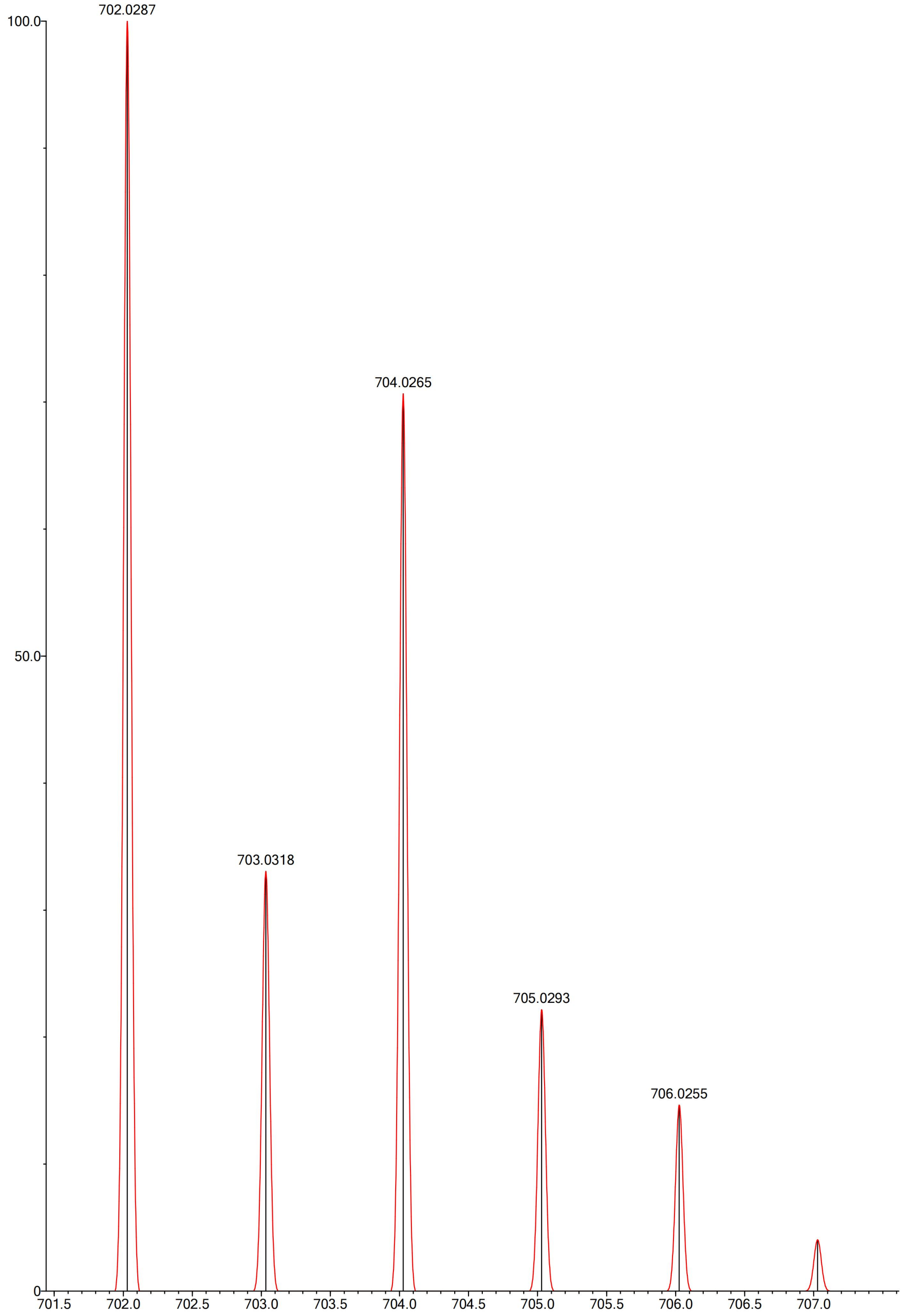

Figure S10 HRMS spectrum of **2**

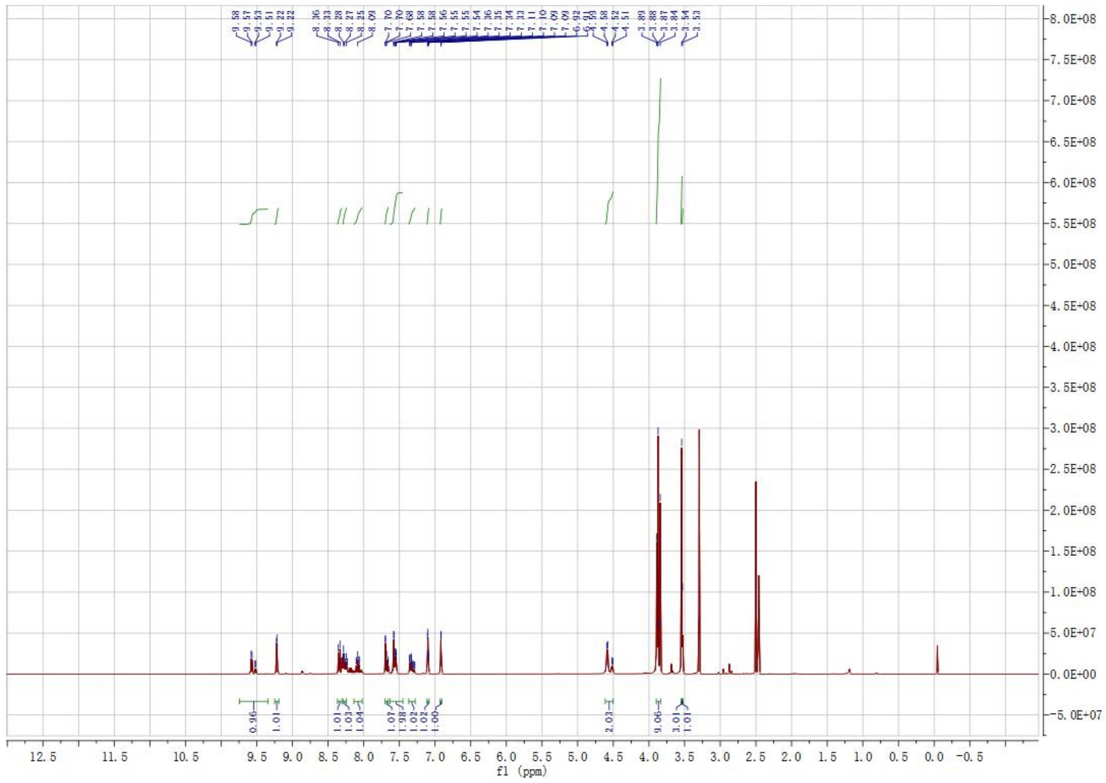

Figure S11 ^{1}H NMR spectrum of **2**

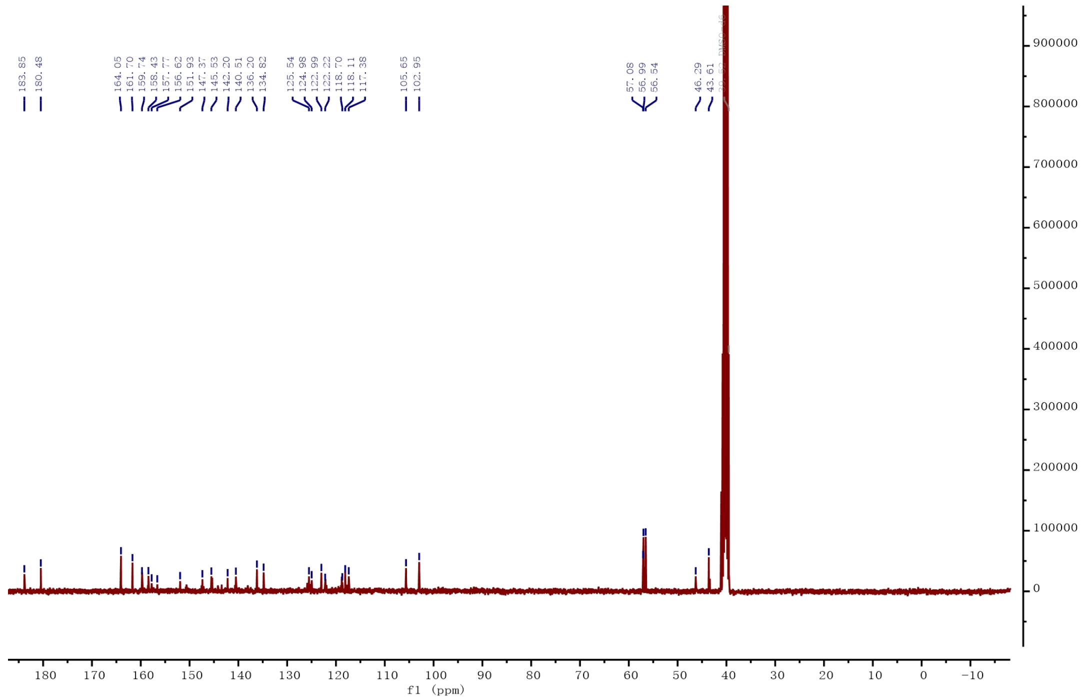

Figure S12 ^{13}C NMR spectrum of **2**

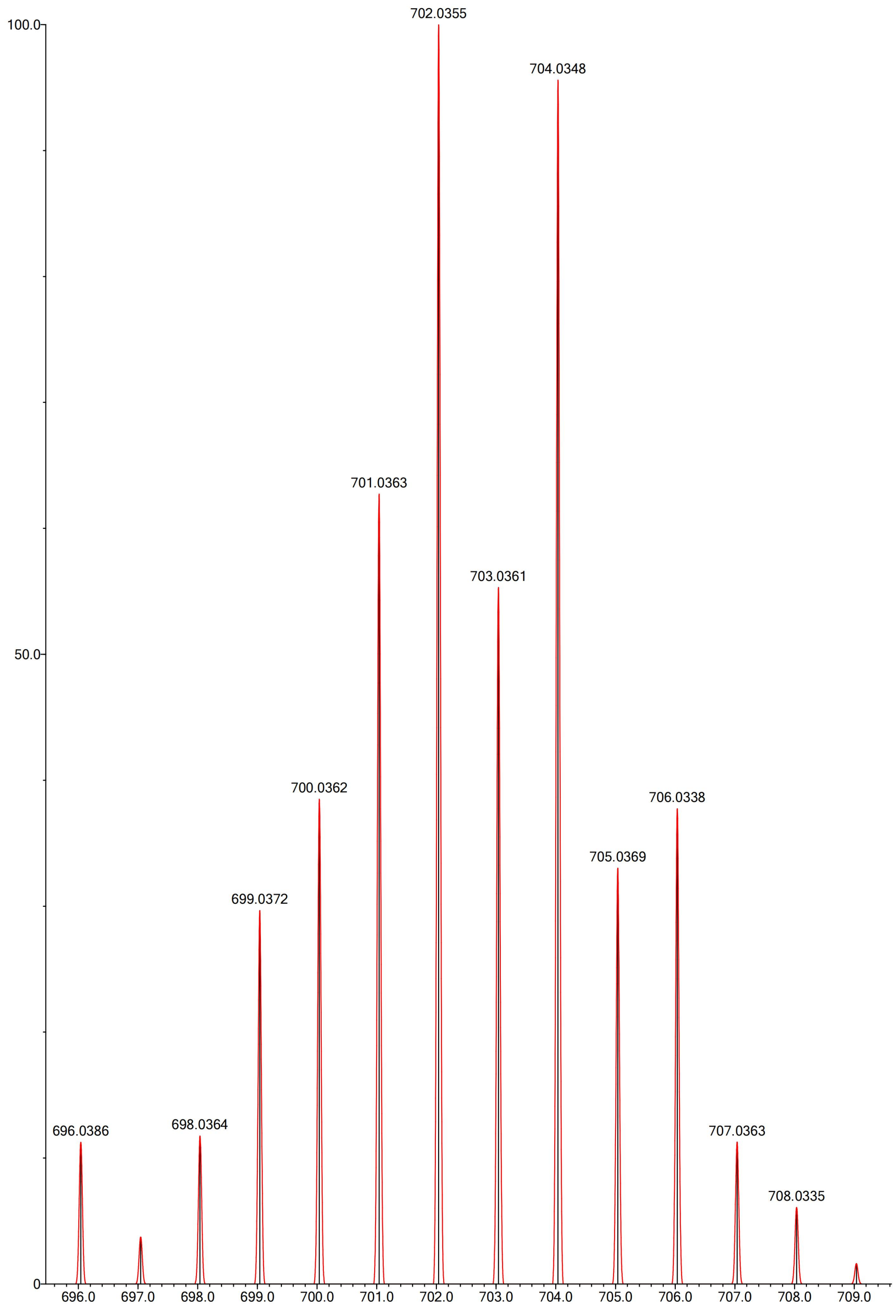

Figure S13 HRMS spectrum of **3**

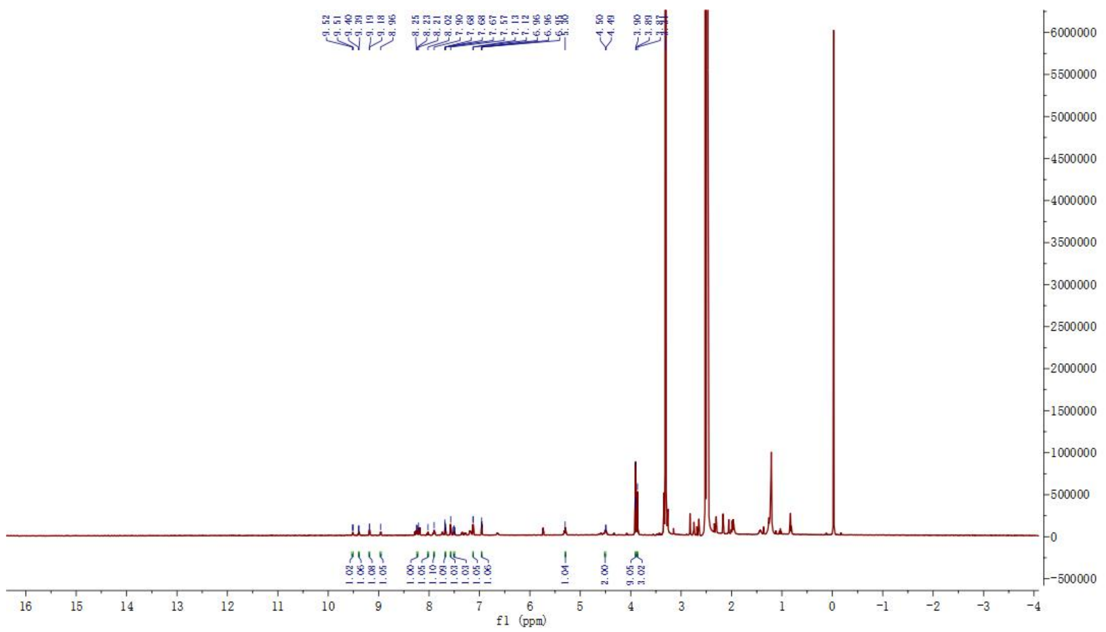

Figure S14 ^{1}H NMR spectrum of **3**

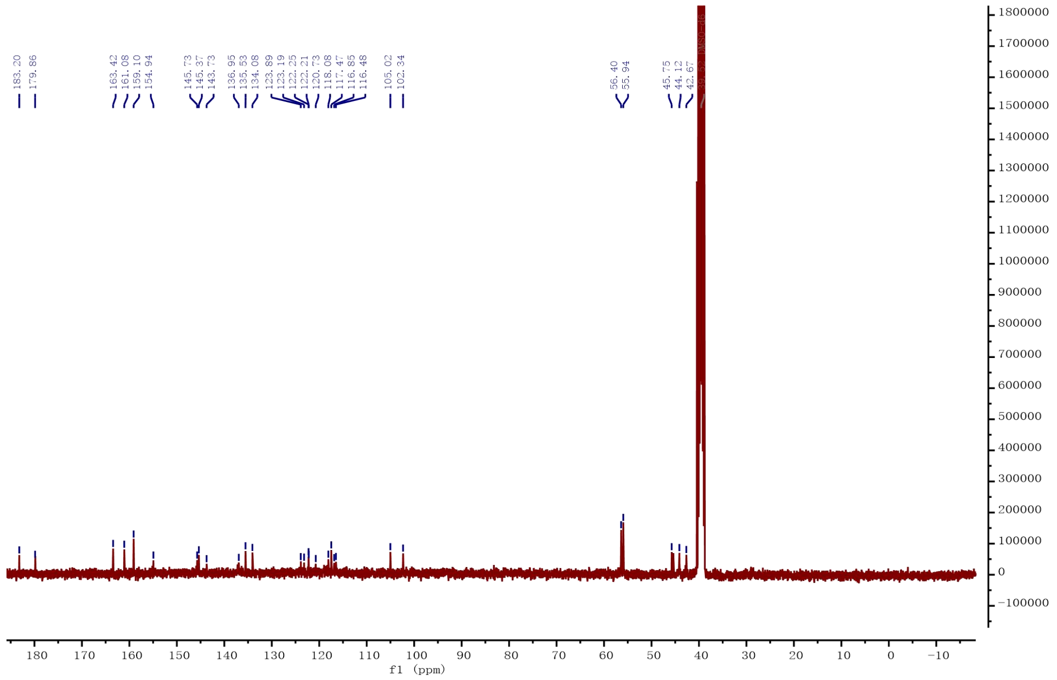

Figure S15 ^{13}C NMR spectrum of **3**

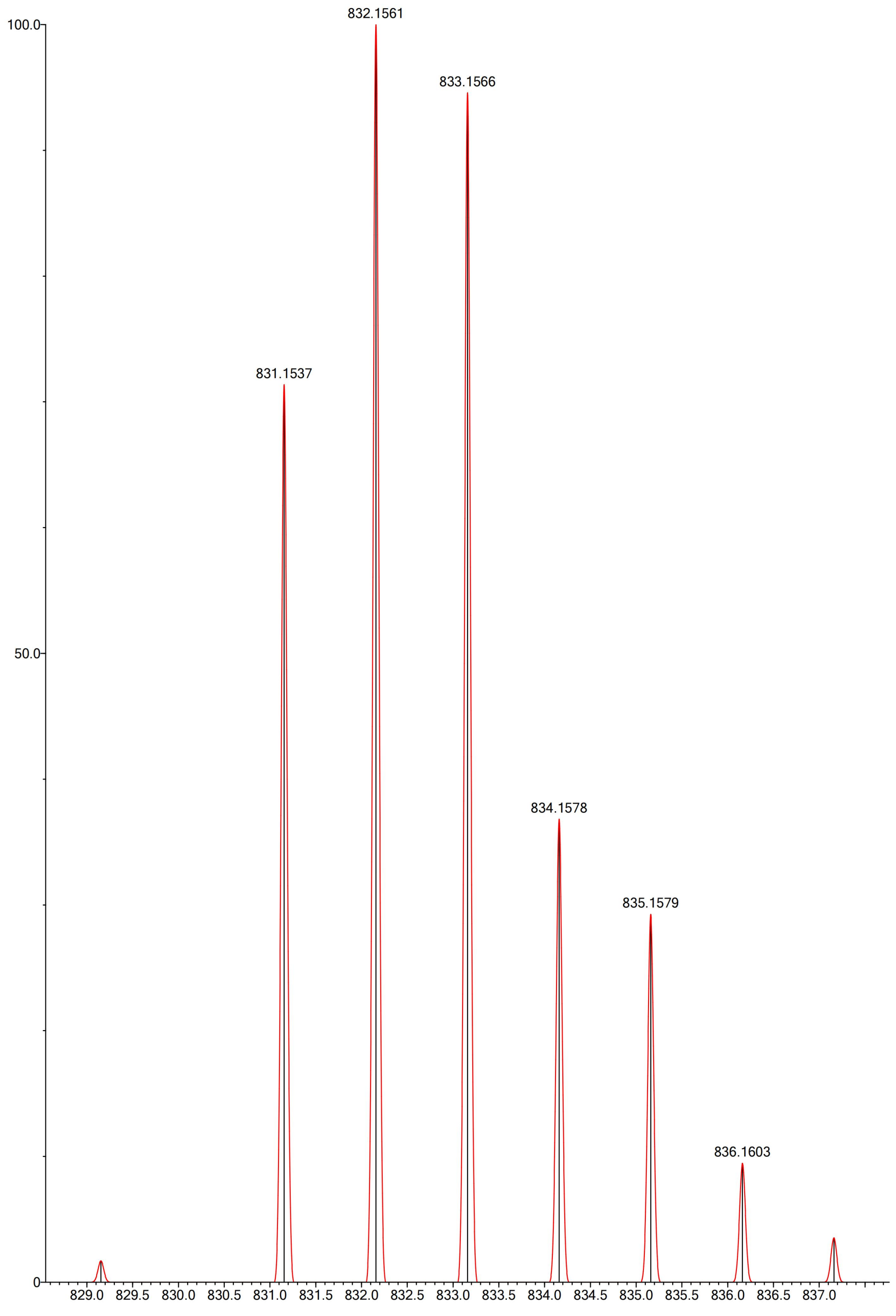

Figure S16 HRMS spectrum of **4**

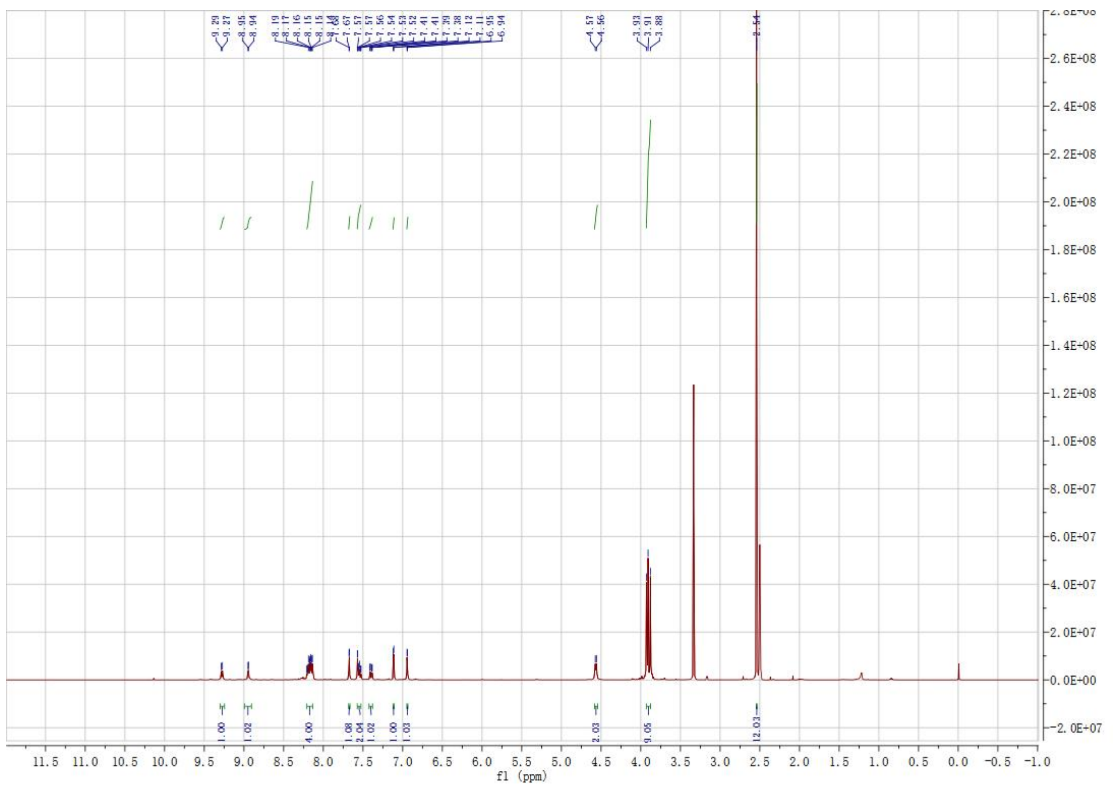

Figure S17 ^{1}H NMR spectrum of **4**

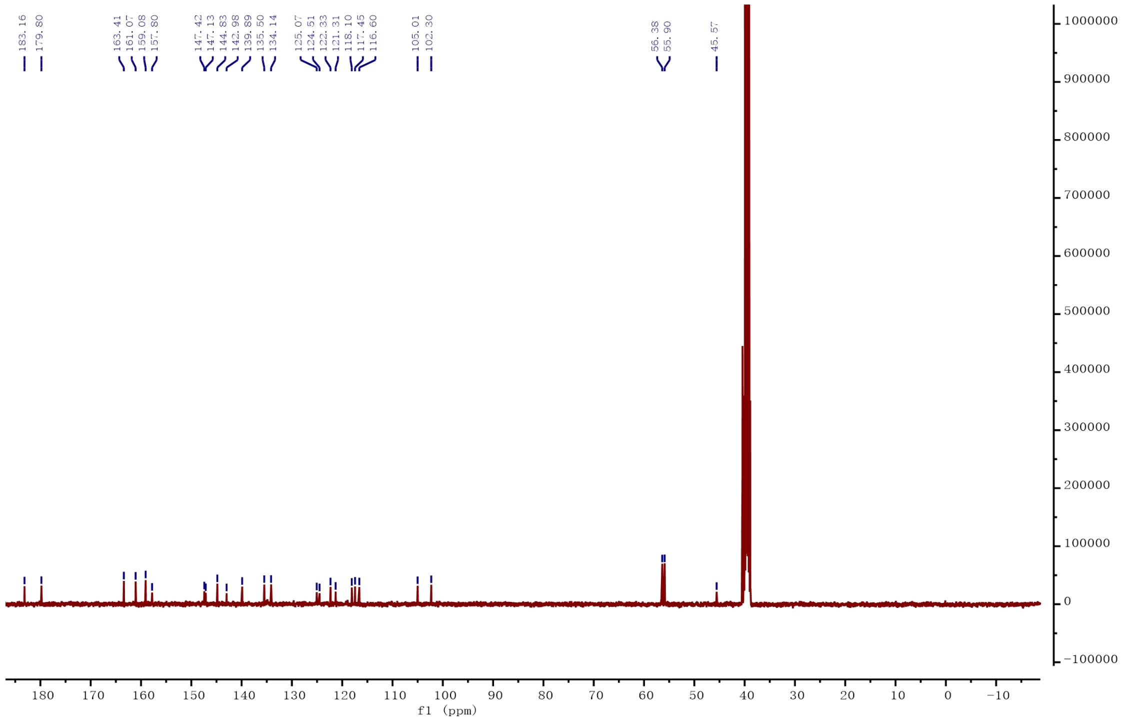

Figure S18 ^{13}C NMR spectrum of **4**

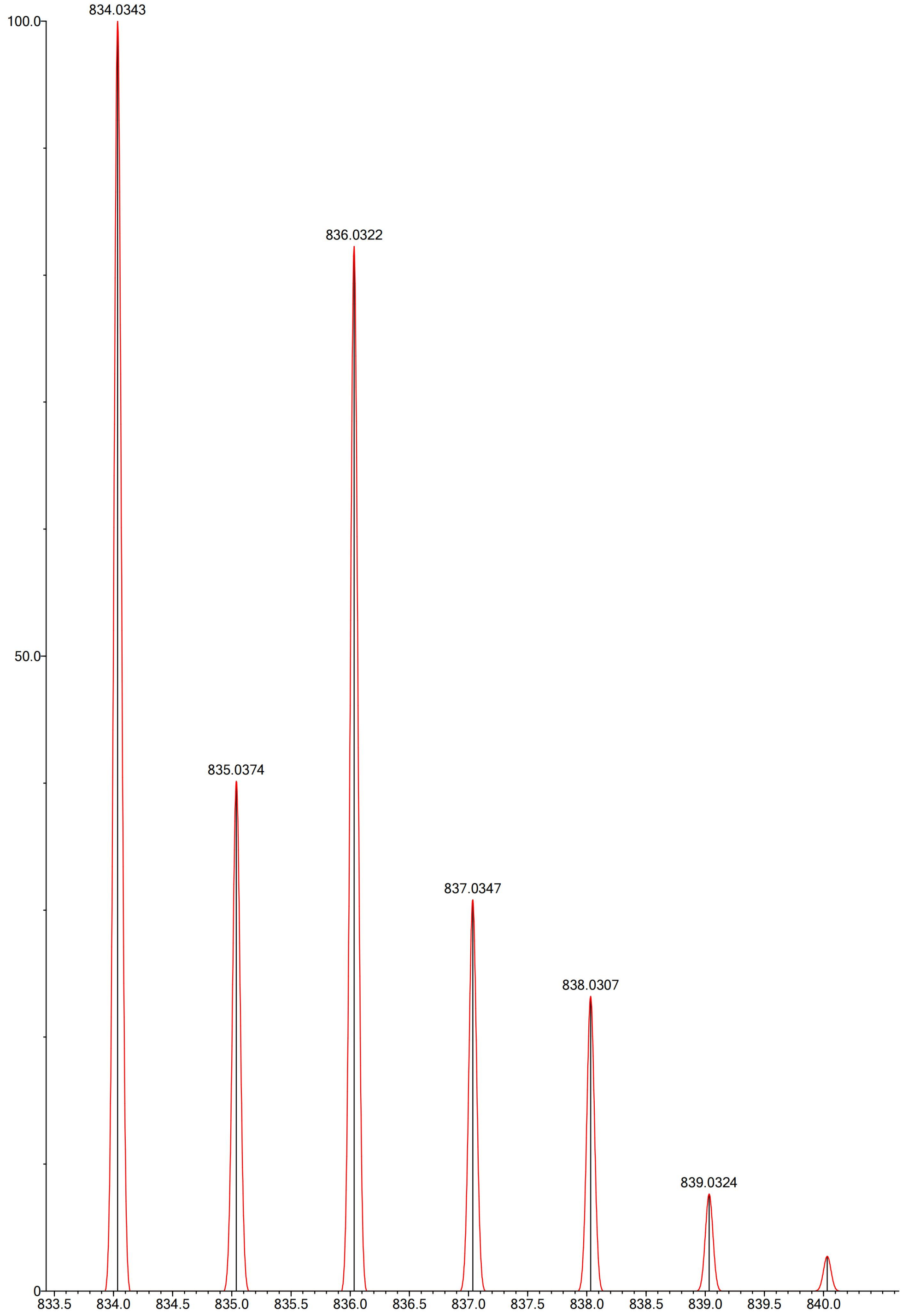

Figure S19 HRMS spectrum of **5**

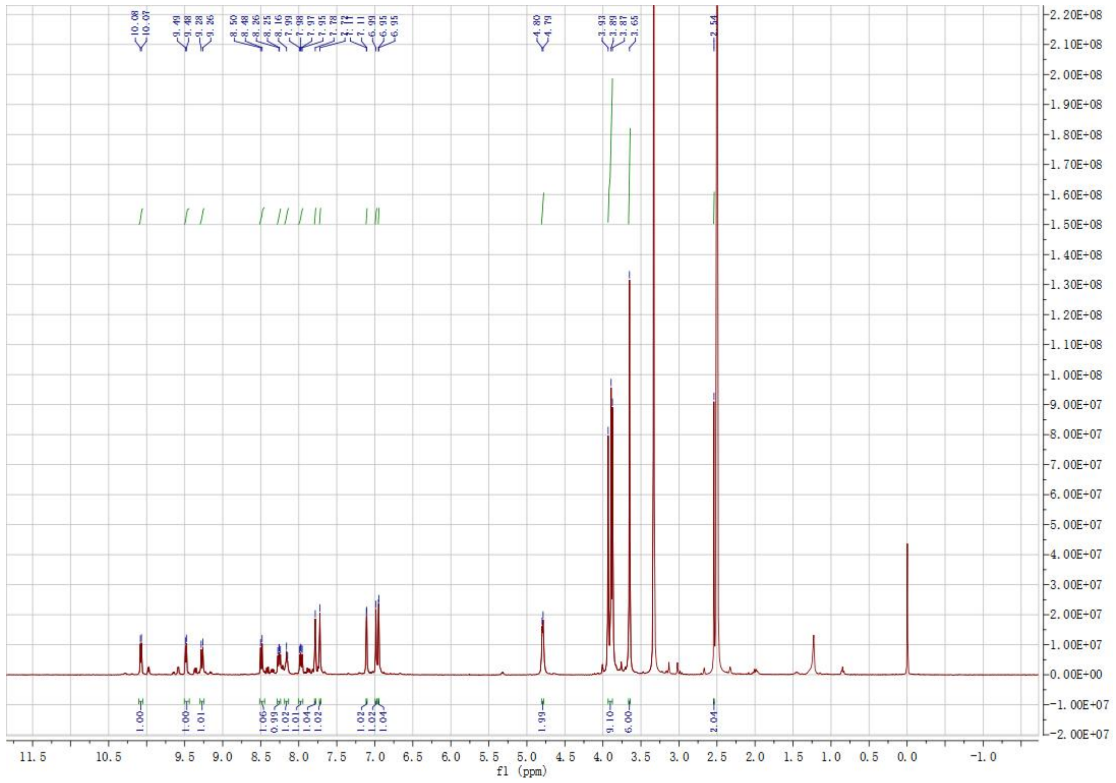

Figure S20 ^{1}H NMR spectrum of **5**

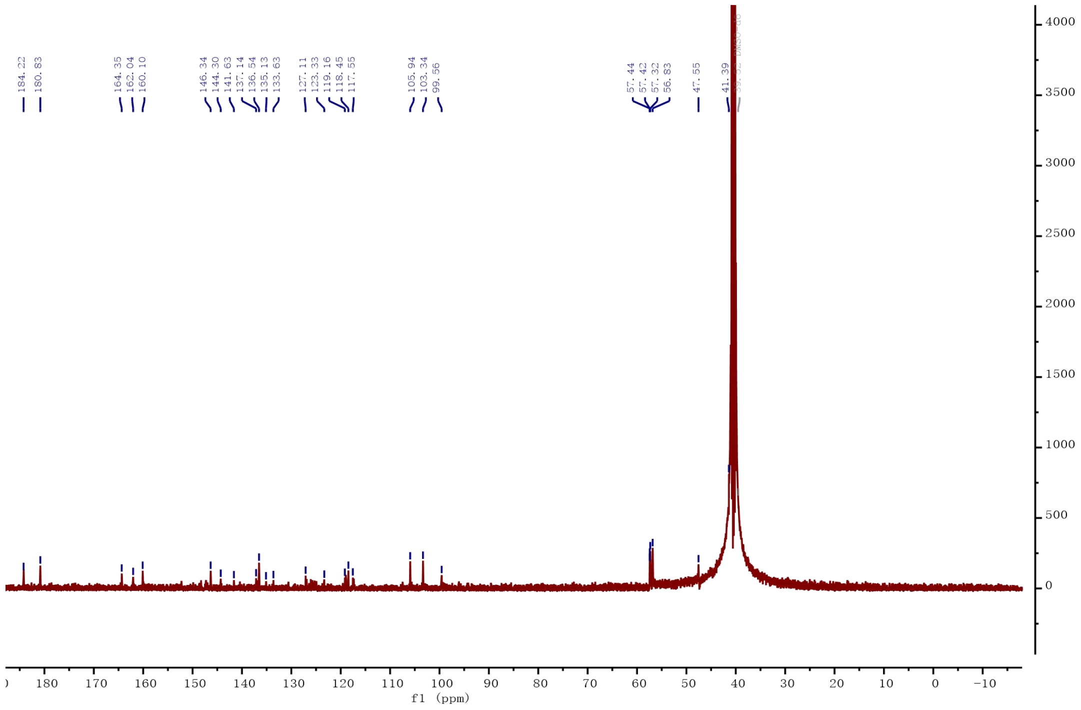

Figure S21 ^{13}C NMR spectrum of **5**

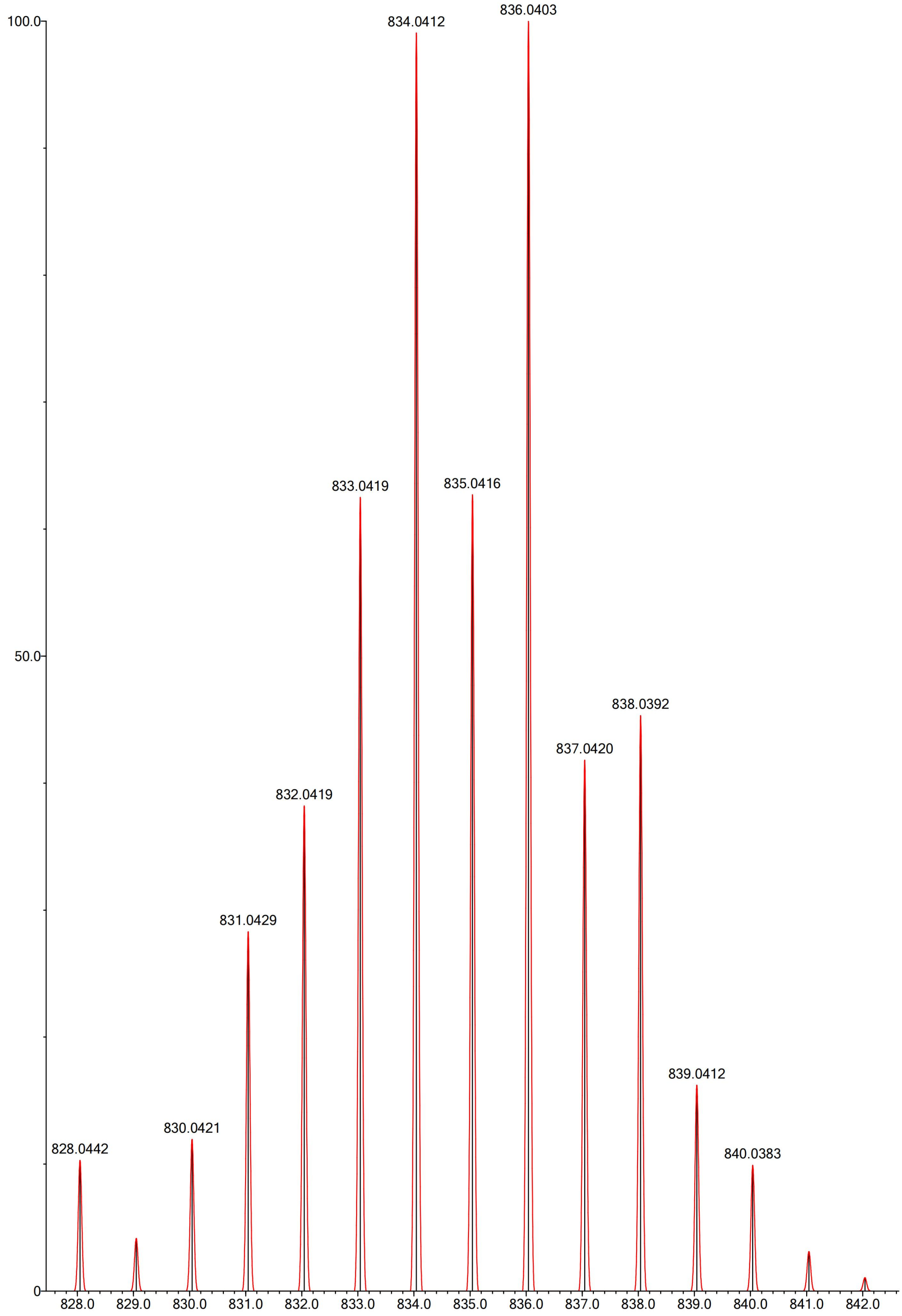

Figure S22 HRMS spectrum of **6**

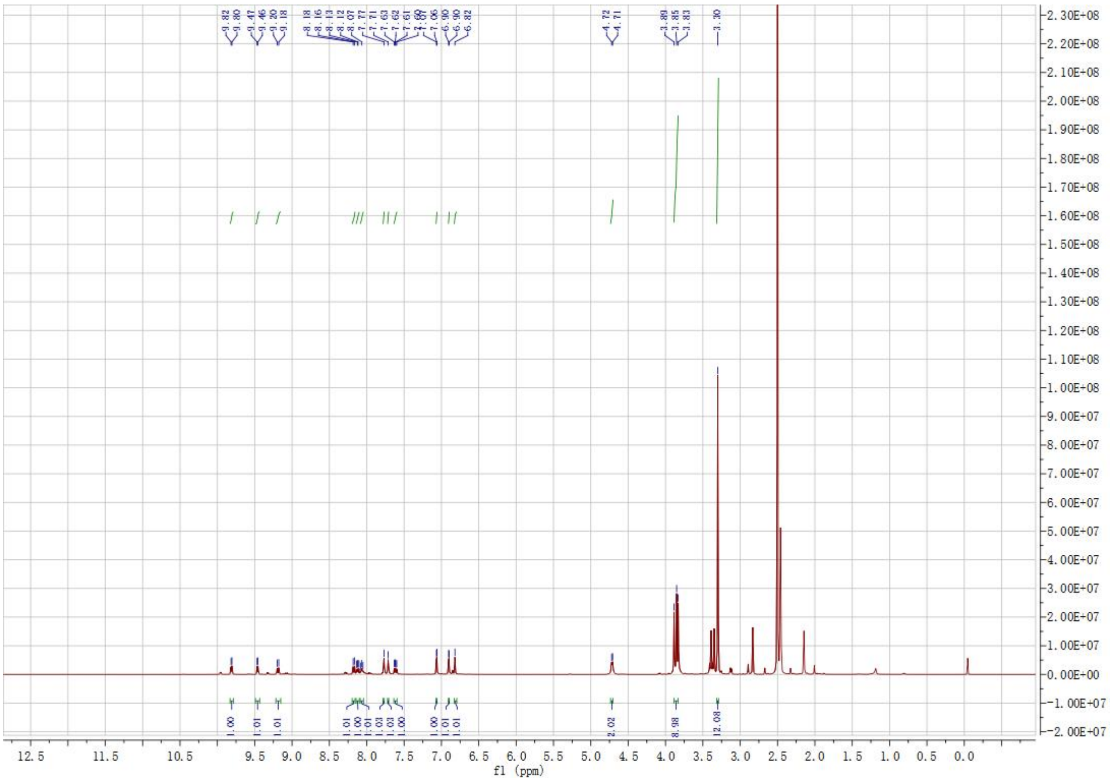

Figure S23 ^{1}H NMR spectrum of **6**

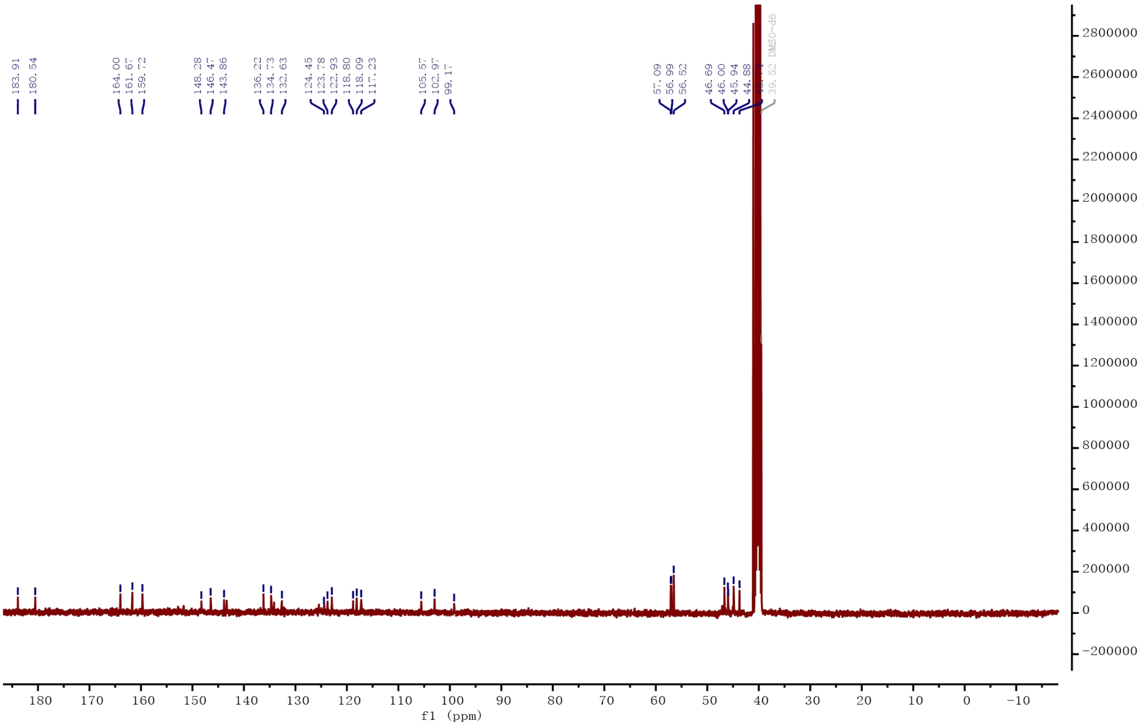

Figure S24 ^{13}C NMR spectrum of **6**

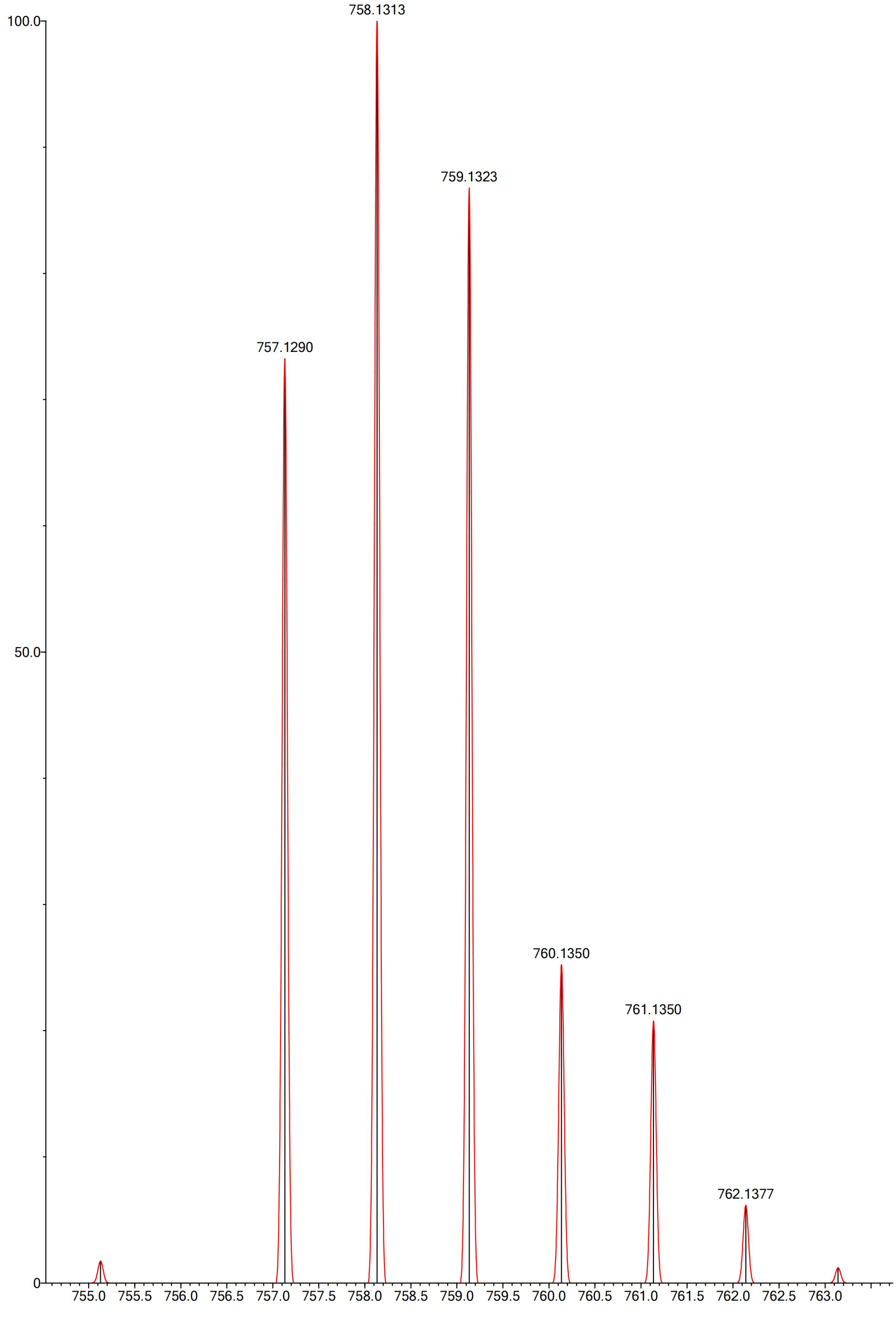

Figure S25 HRMS spectrum of **7**

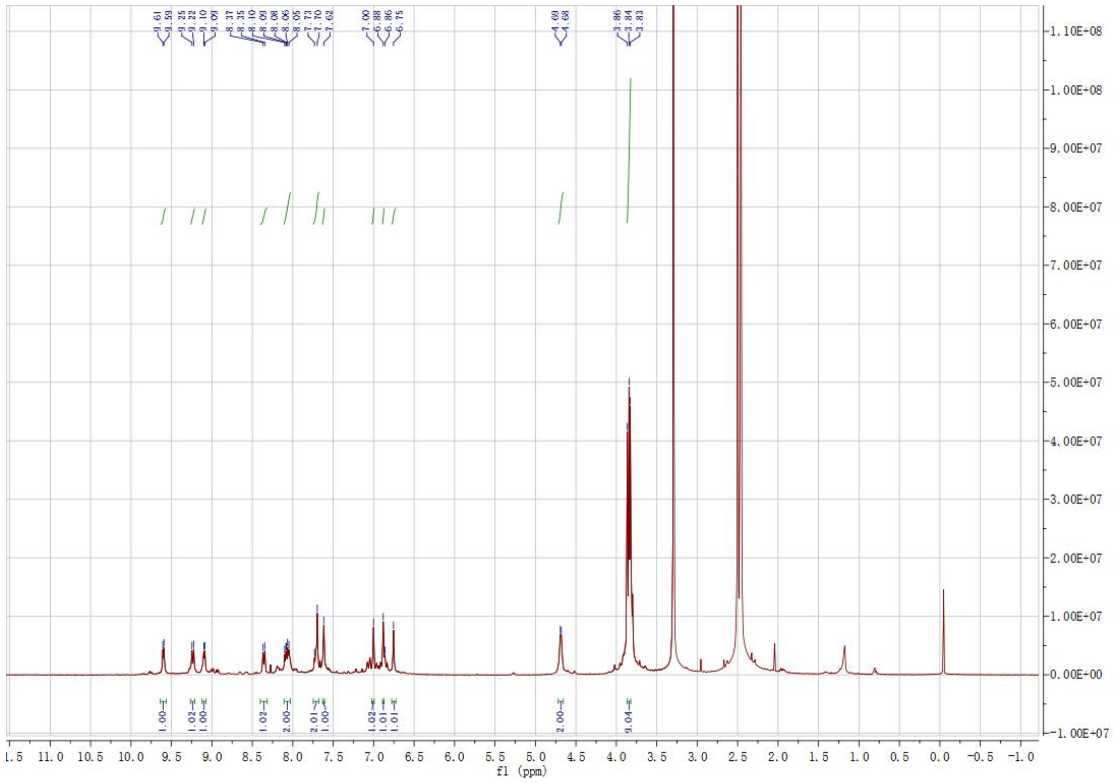

Figure S26 ^{1}H NMR spectrum of **7**

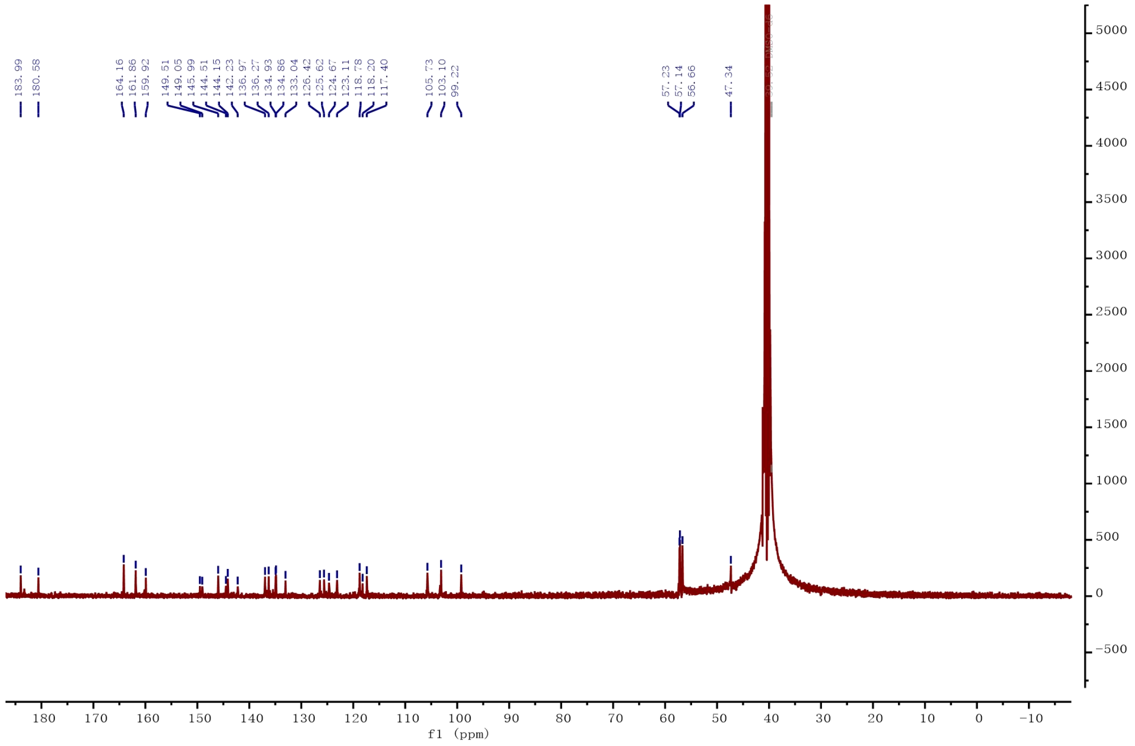

Figure S27 ^{13}C NMR spectrum of **7**

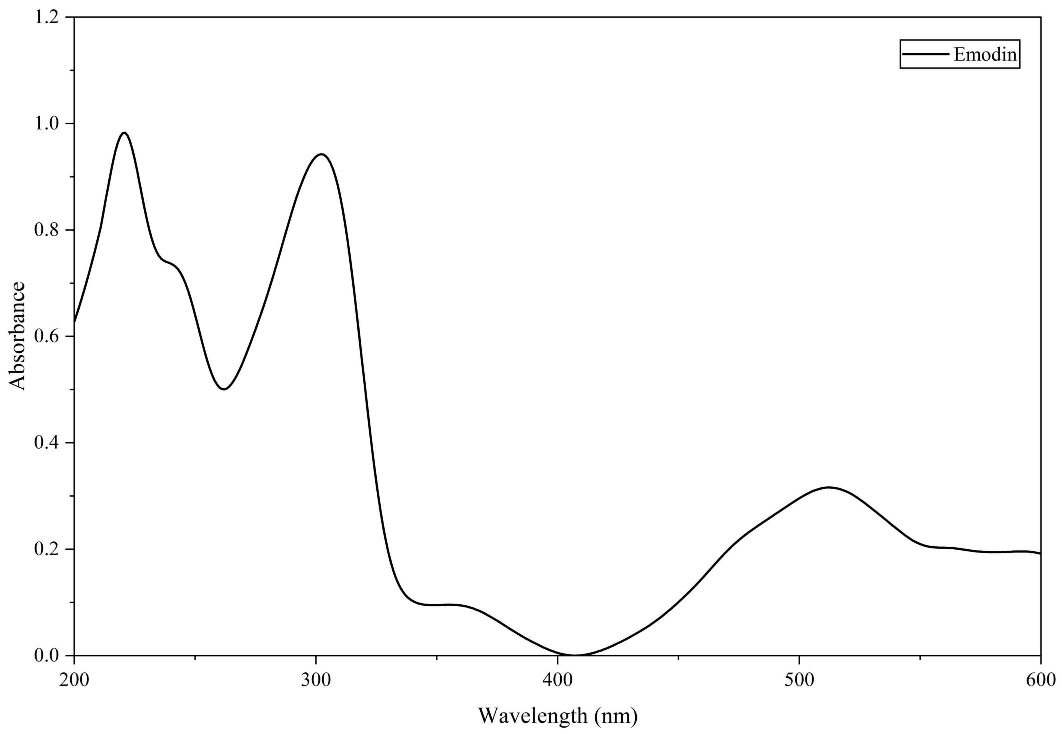

Figure S28 UV-Vis absorption spectrum of **Emodin**

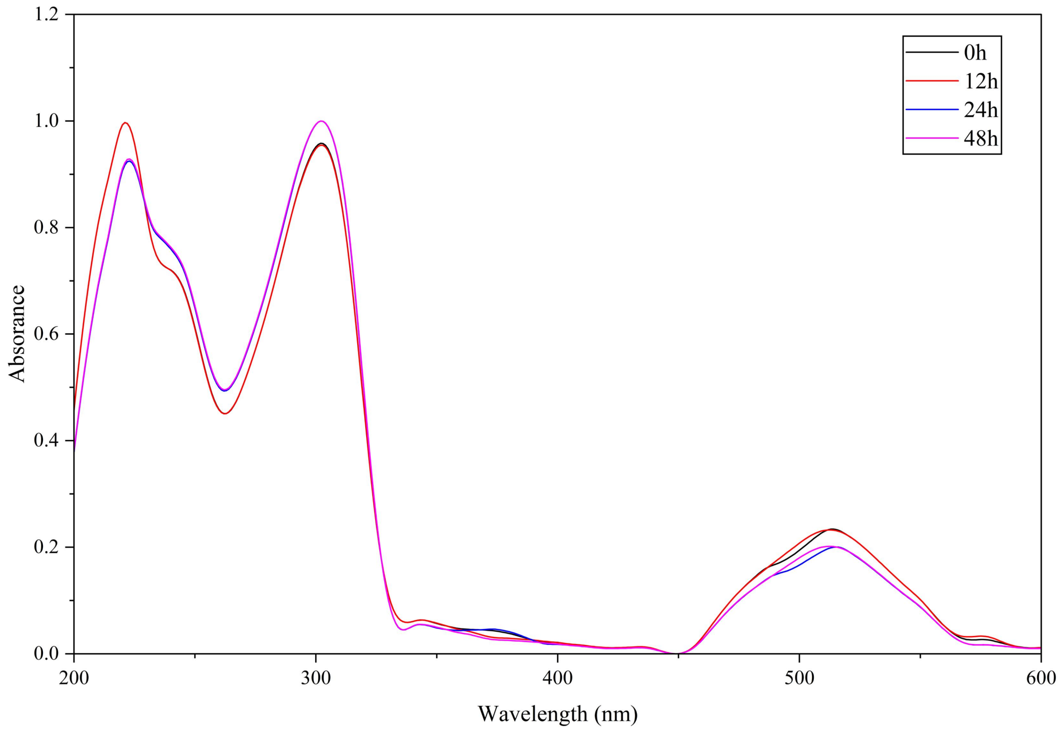

Figure S29 UV-Vis absorption spectra of **Emodin** recorded at different time intervals

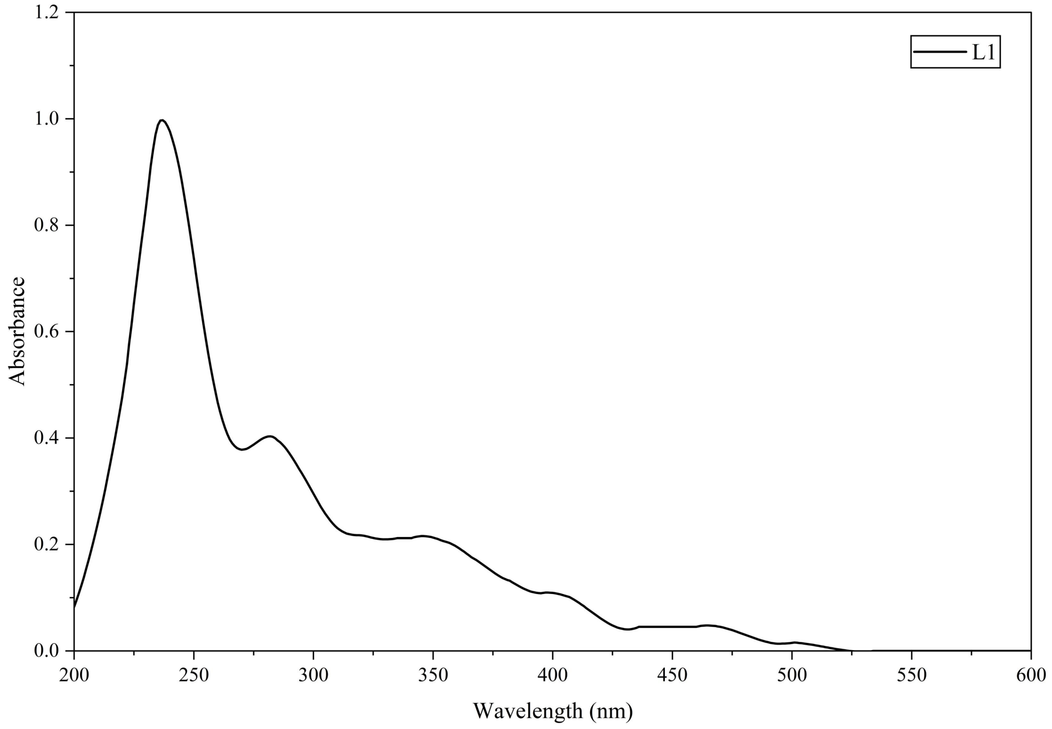

Figure S30 UV-Vis absorption spectrum of **L1**

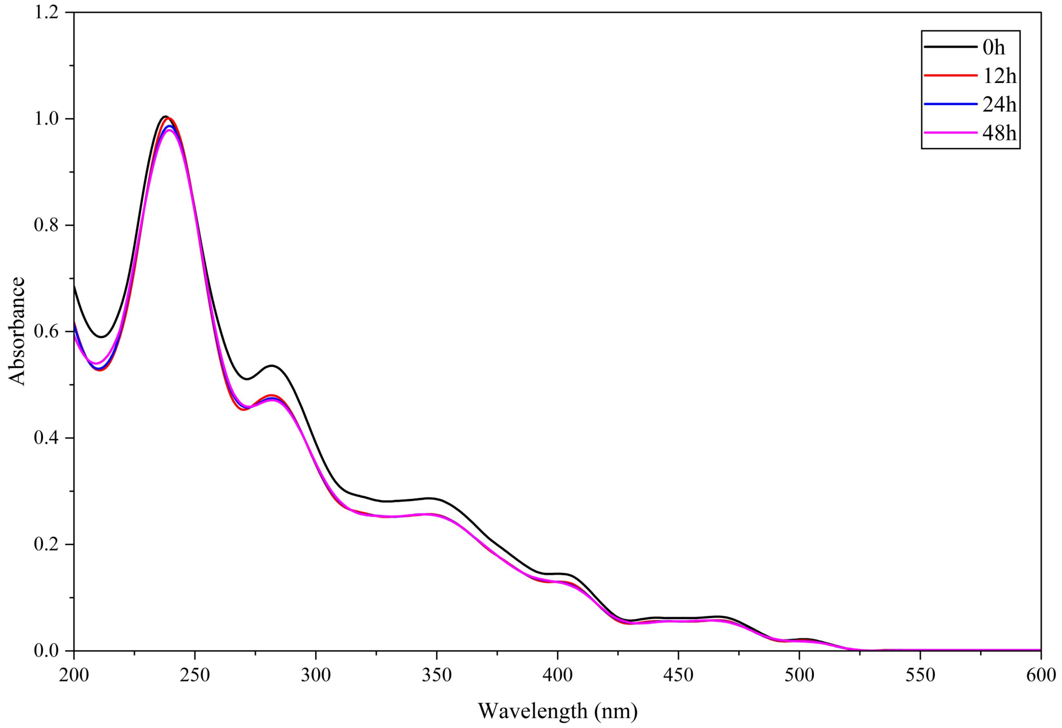

Figure S31 UV-Vis absorption spectra of **L1** recorded at different time intervals

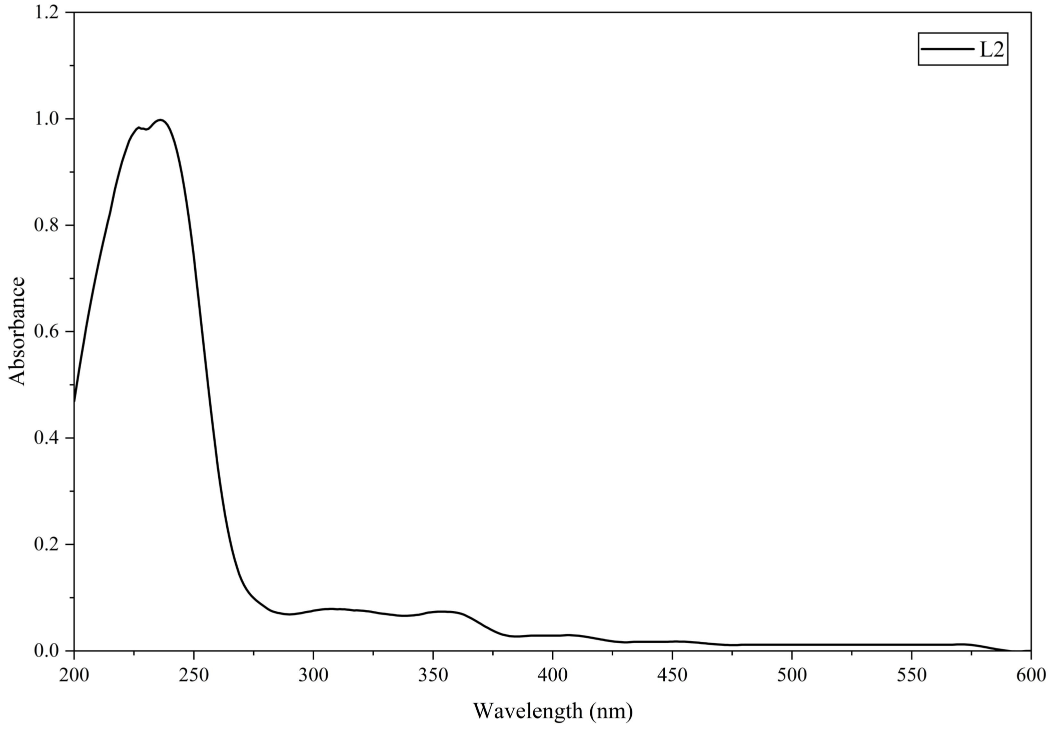

Figure S32 UV-Vis absorption spectrum of **L2**

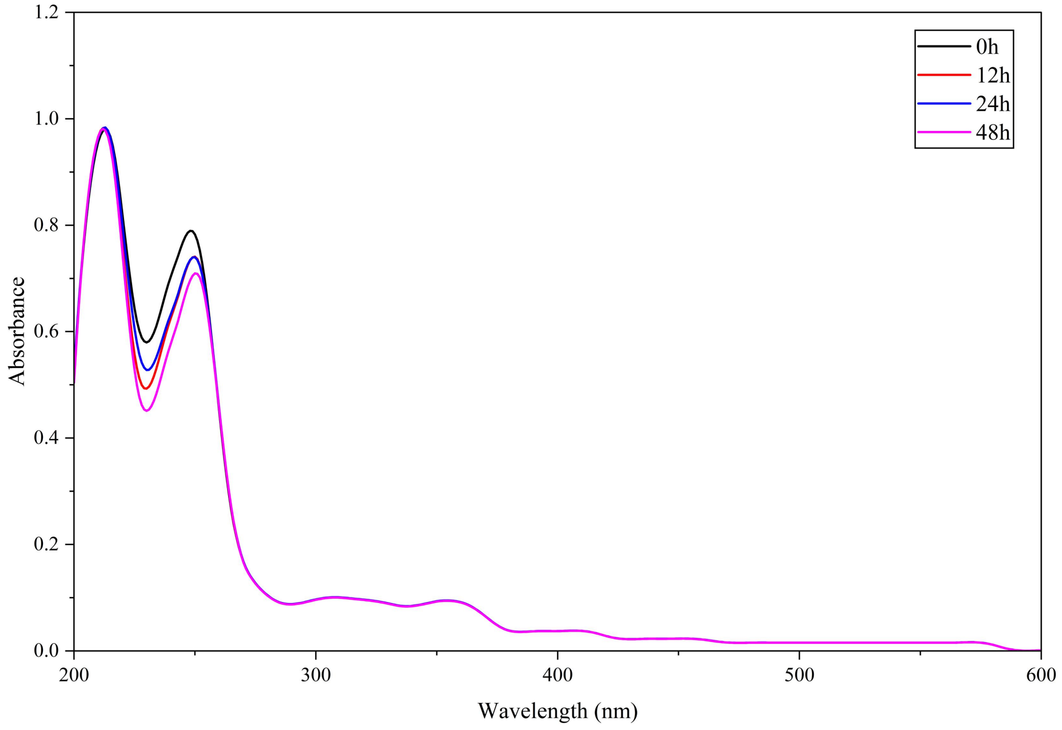

Figure S33 UV-Vis absorption spectra of **L2** recorded at different time intervals

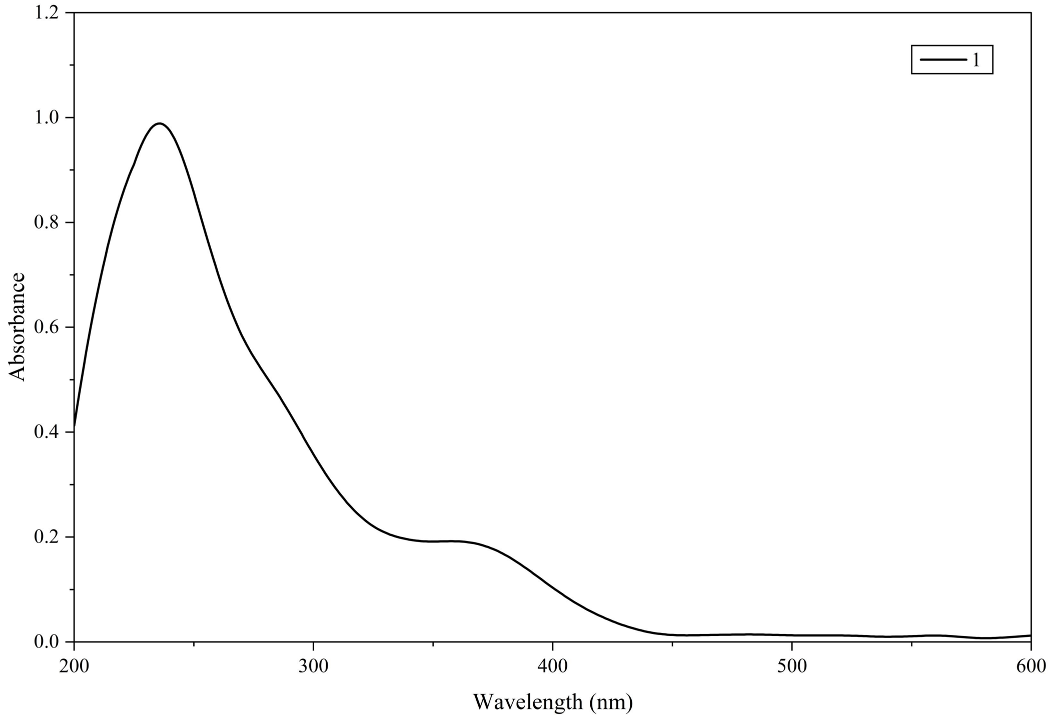

Figure S34 UV-Vis absorption spectrum of **1**

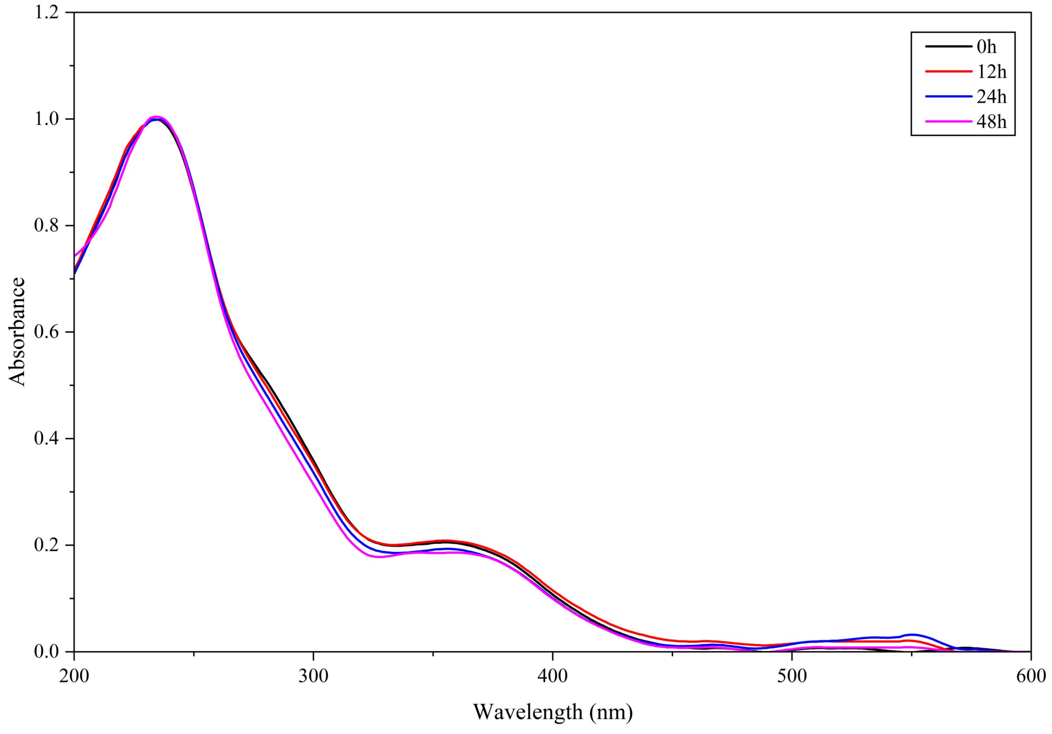

Figure S35 UV-Vis absorption spectra of **1** recorded at different time intervals

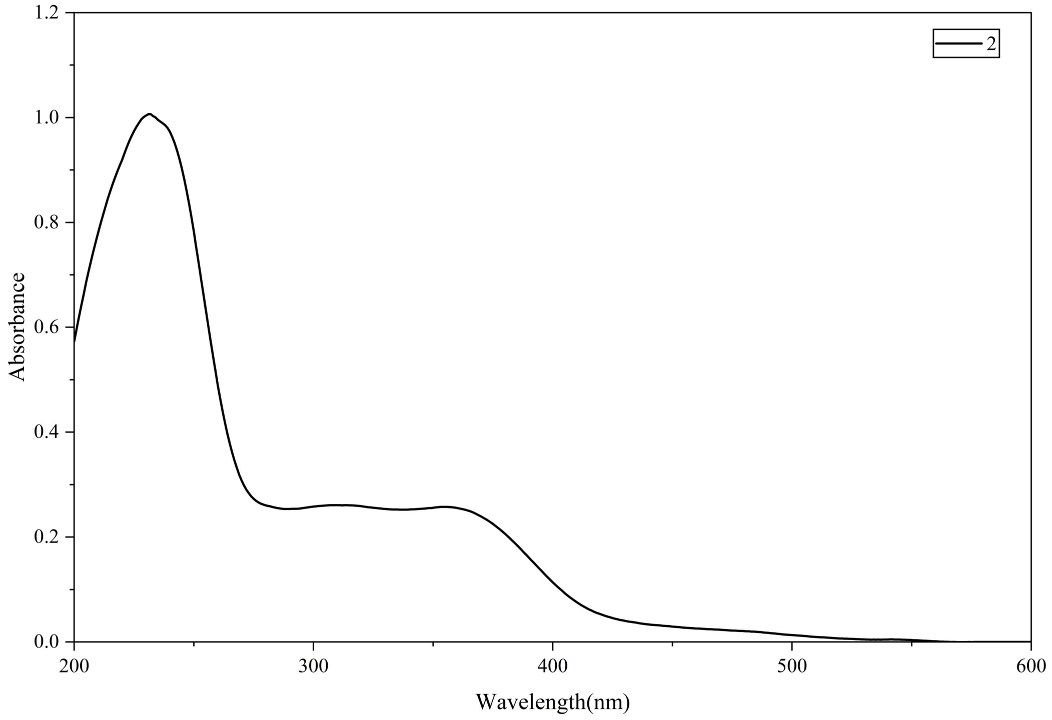

Figure S36 UV-Vis absorption spectrum of **2**

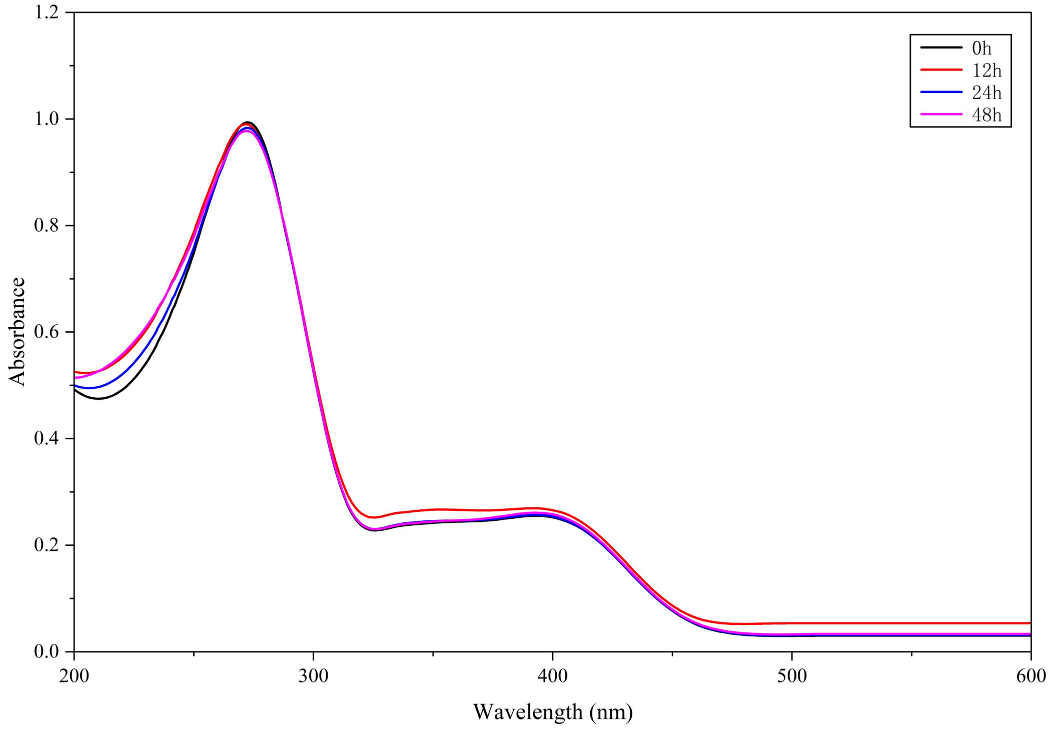

Figure S37 UV-Vis absorption spectra of **2** recorded at different time intervals

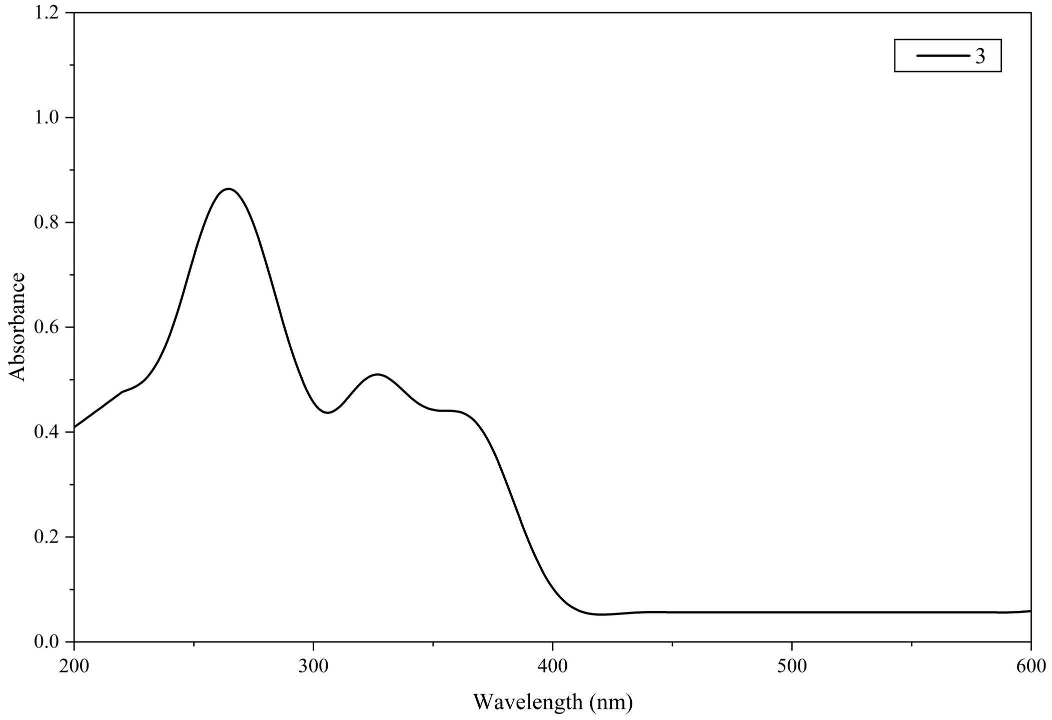

Figure S38 UV-Vis absorption spectrum of **3**

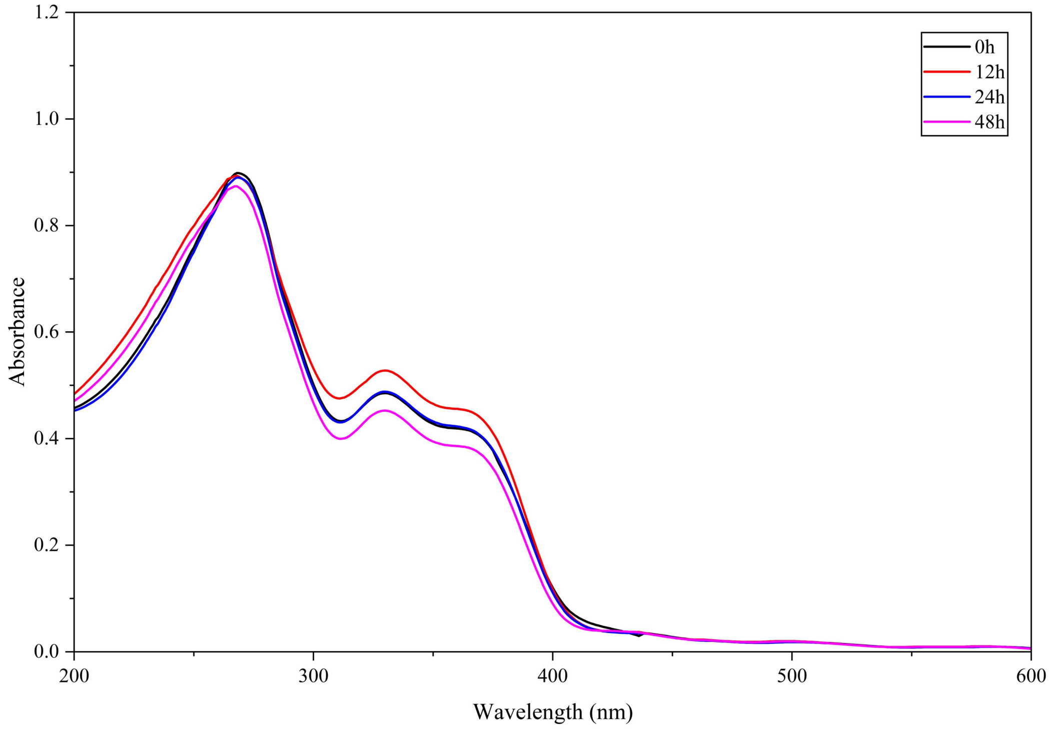

Figure S39 UV-Vis absorption spectra of **3** recorded at different time intervals

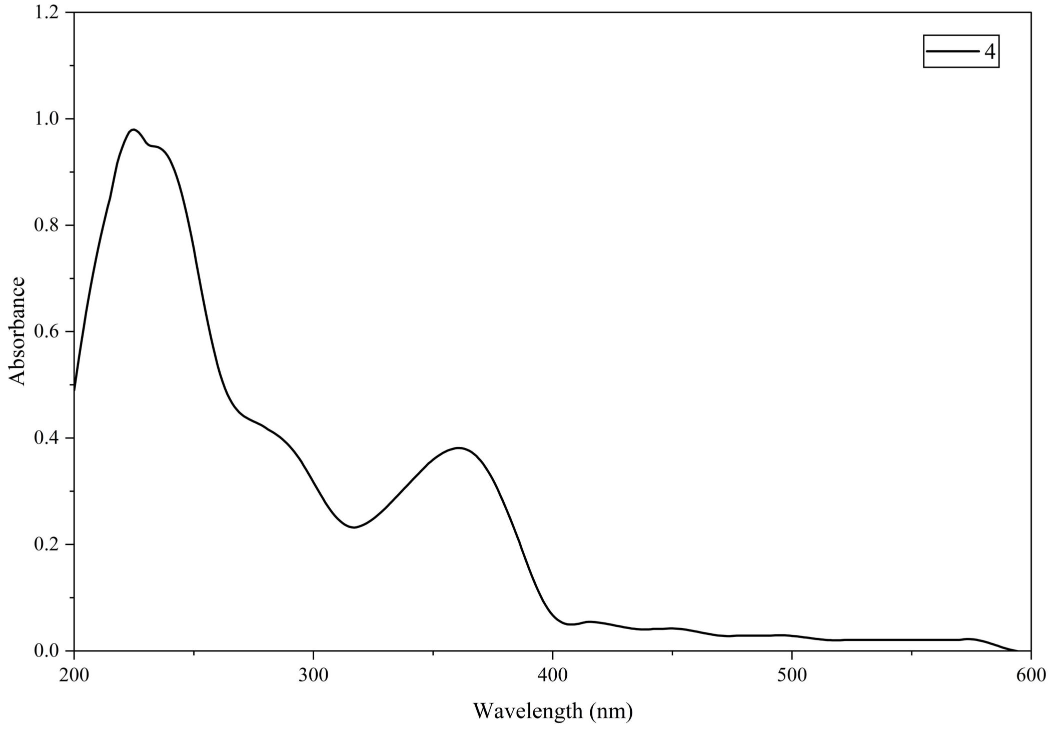

Figure S40 UV-Vis absorption spectrum of **4**

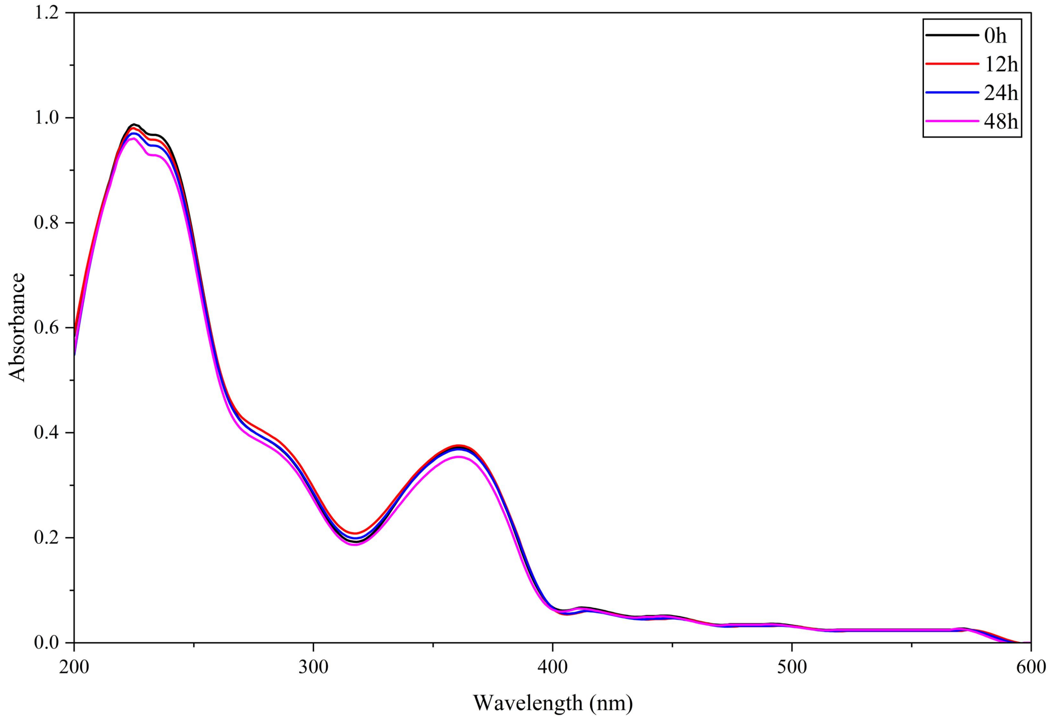

Figure S41 UV-Vis absorption spectra of **4** recorded at different time intervals

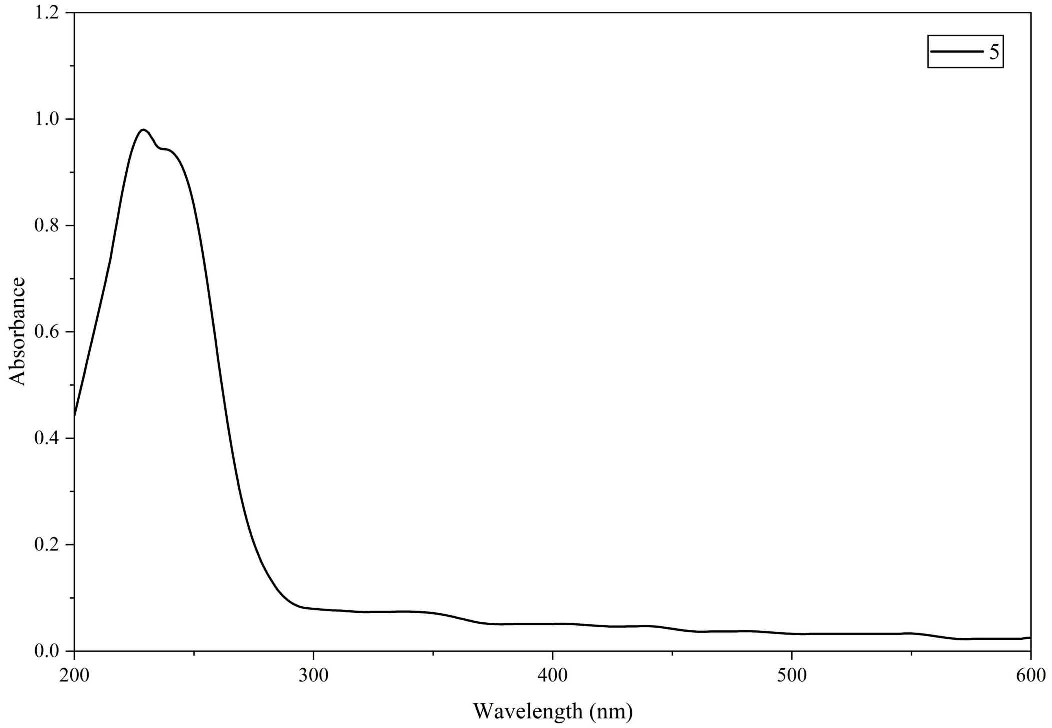

Figure S42 UV-Vis absorption spectrum of **5**

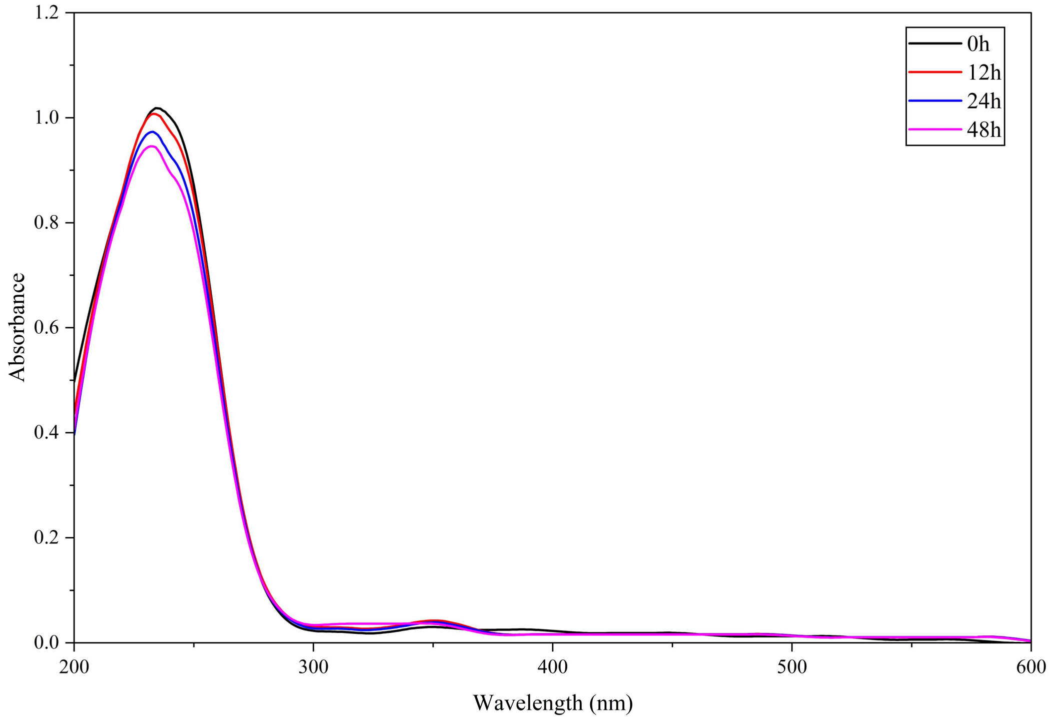

Figure S43 UV-Vis absorption spectra of **5** recorded at different time intervals

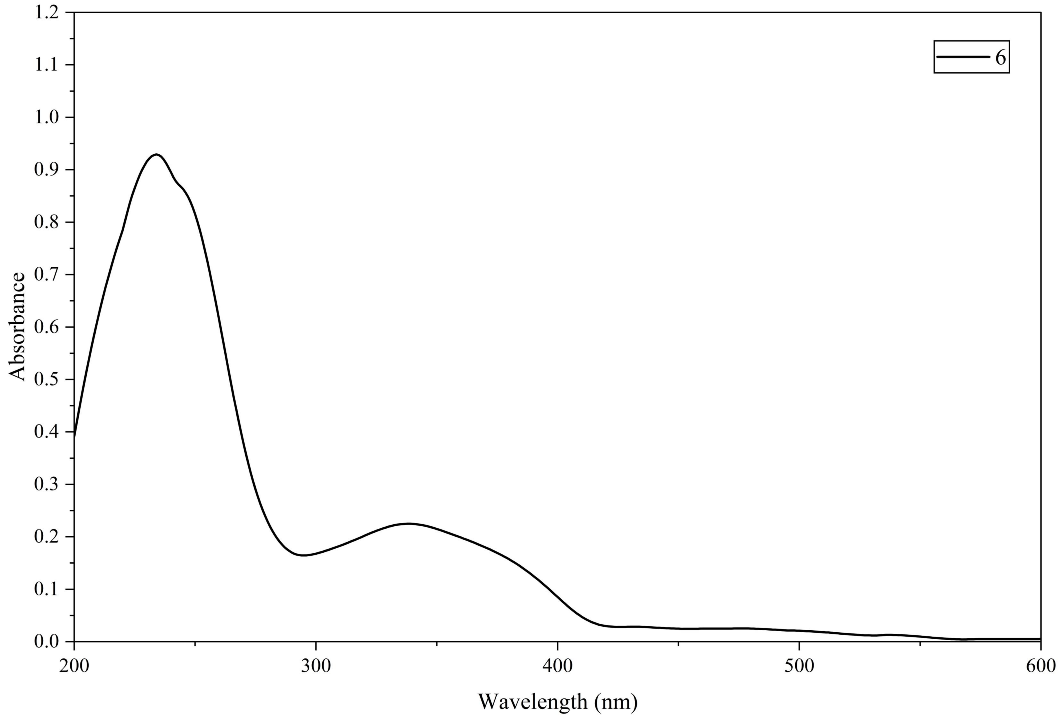

Figure S44 UV-Vis absorption spectrum of **6**

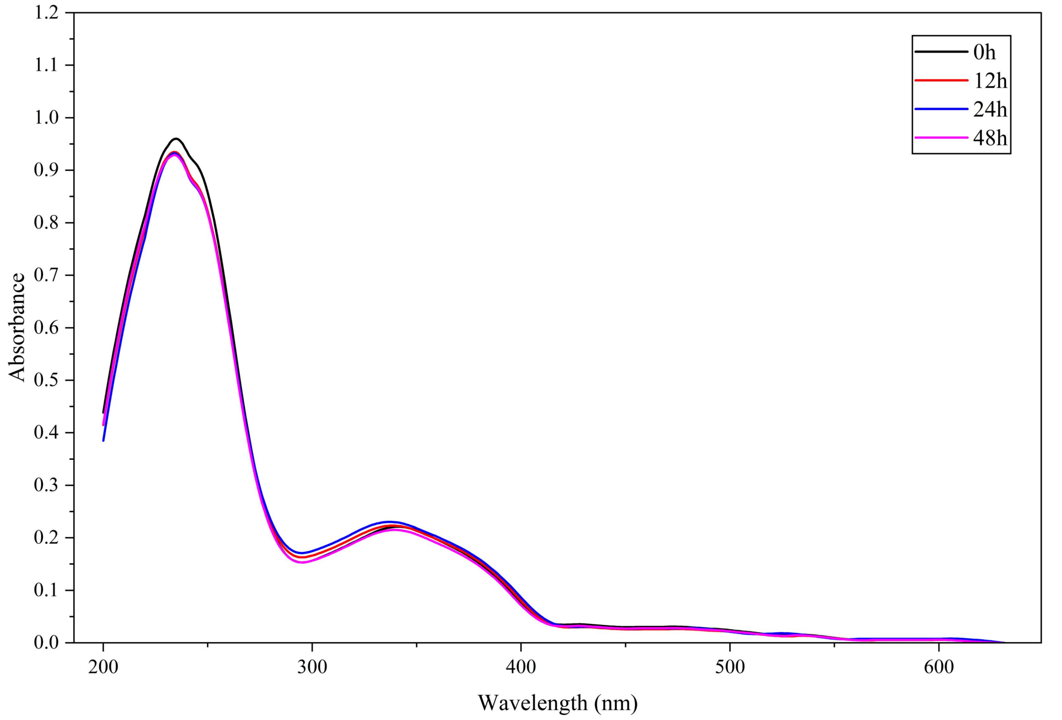

Figure S45 UV-Vis absorption spectra of **6** recorded at different time intervals

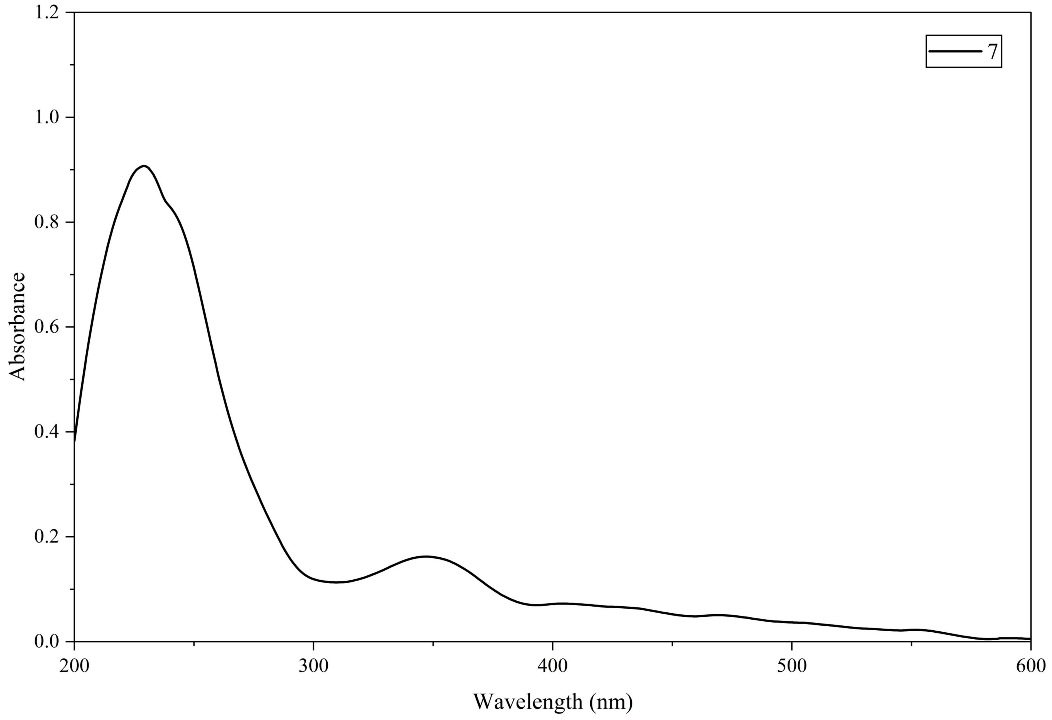

Figure S46 UV-Vis absorption spectrum of **7**

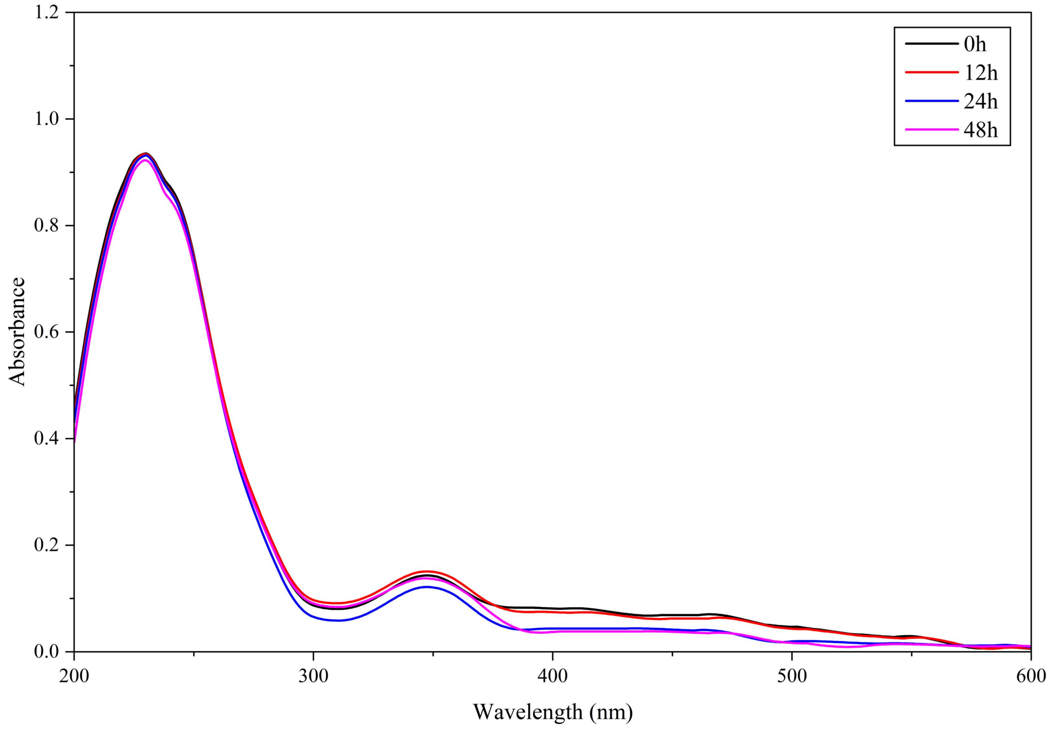

Figure S47 UV-Vis absorption spectra of **7** recorded at different time intervals

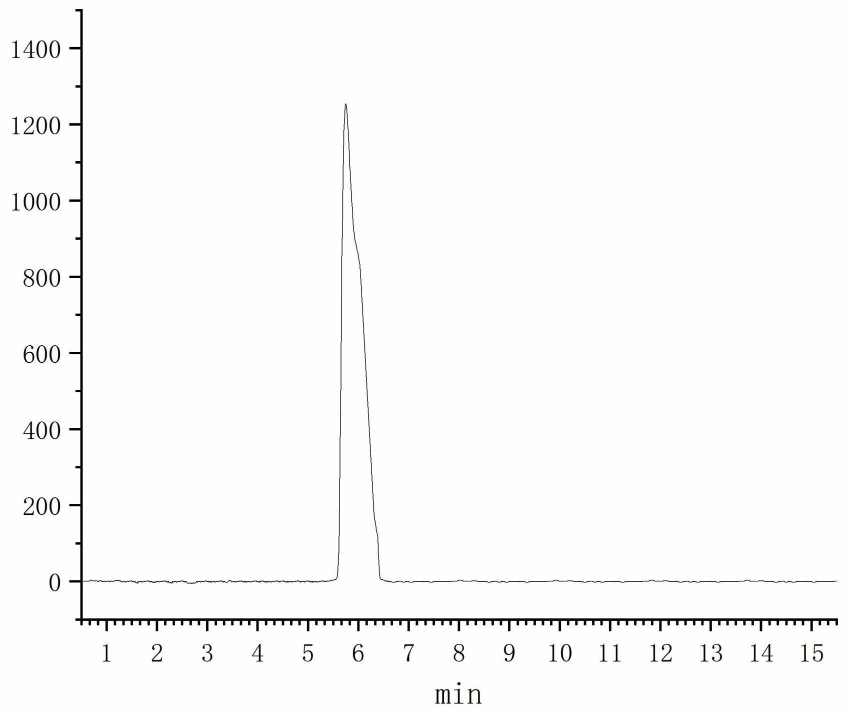

Figure S48 The HPLC chromatograms of **L1**

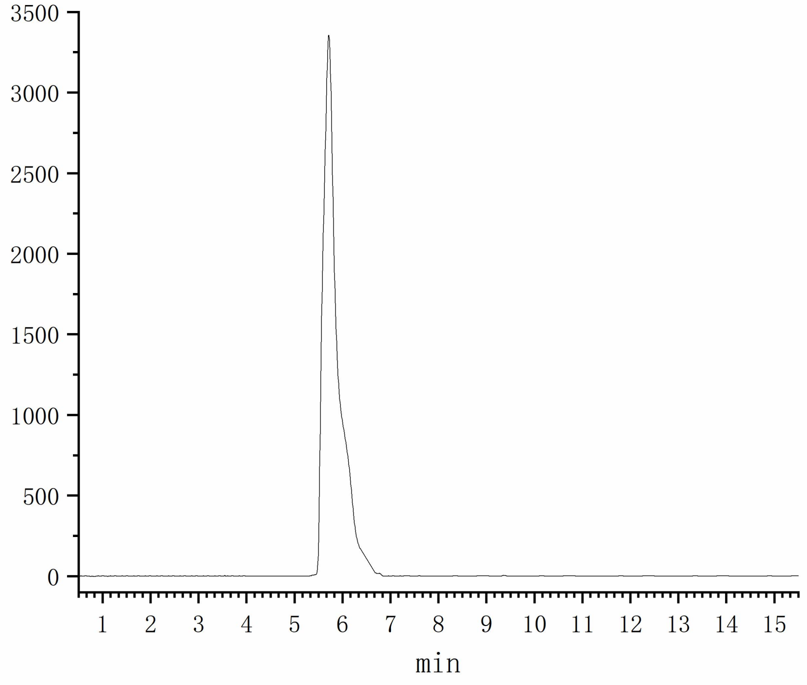

Figure S49 The HPLC chromatograms of **L2**

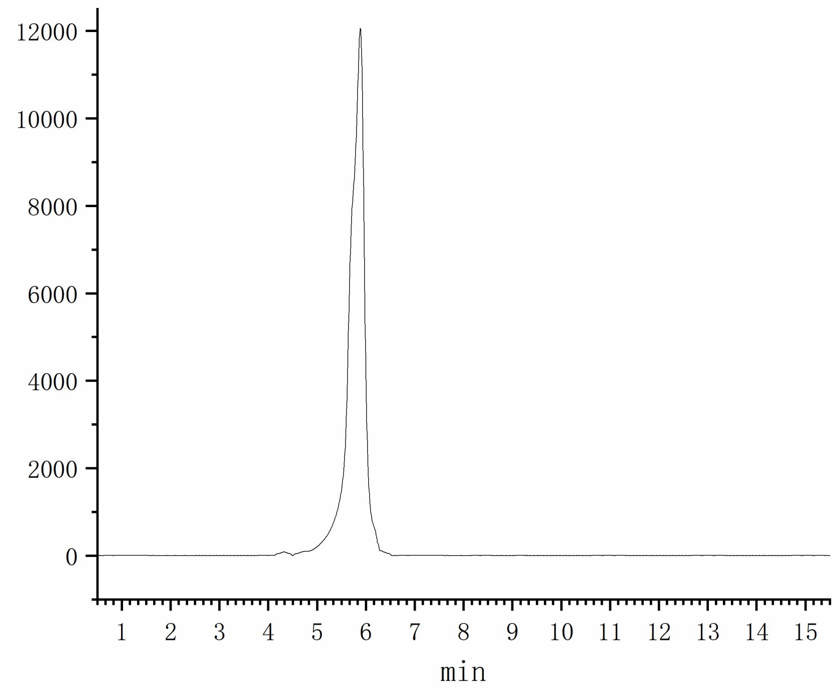

Figure S50 The HPLC chromatograms of **1**

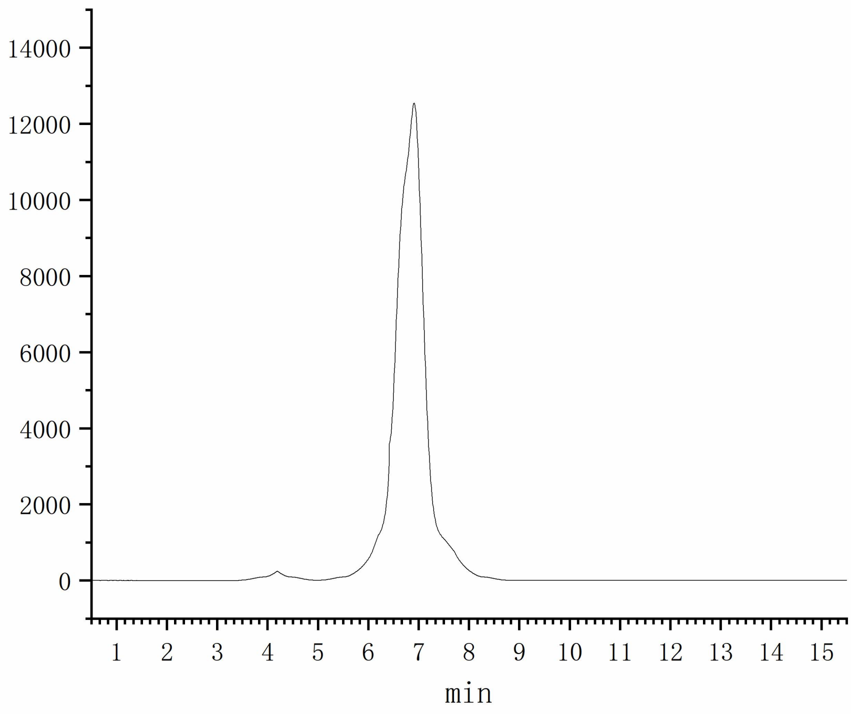

Figure S51 The HPLC chromatograms of **2**

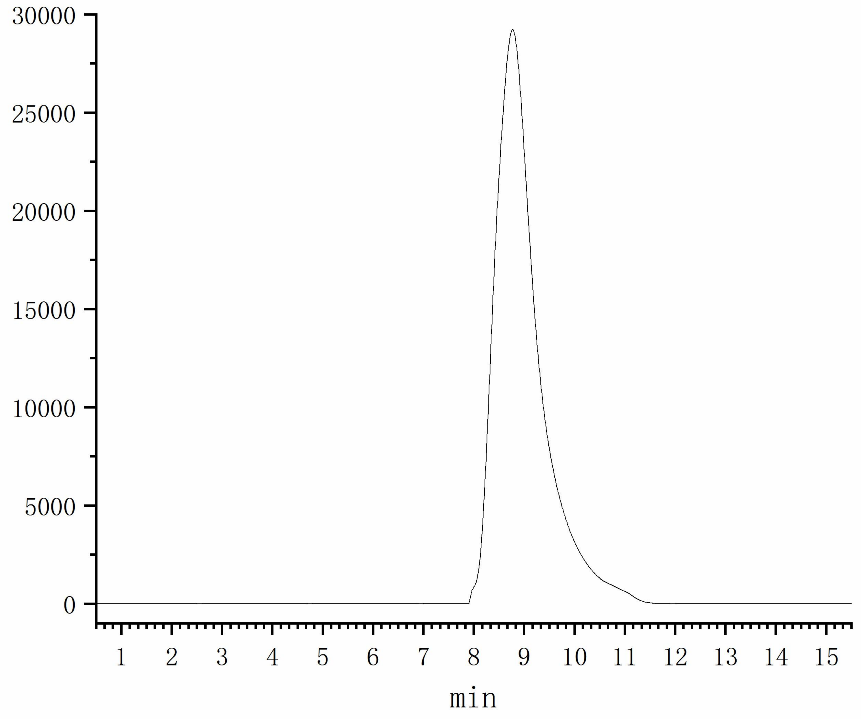

Figure S52 The HPLC chromatograms of **3**

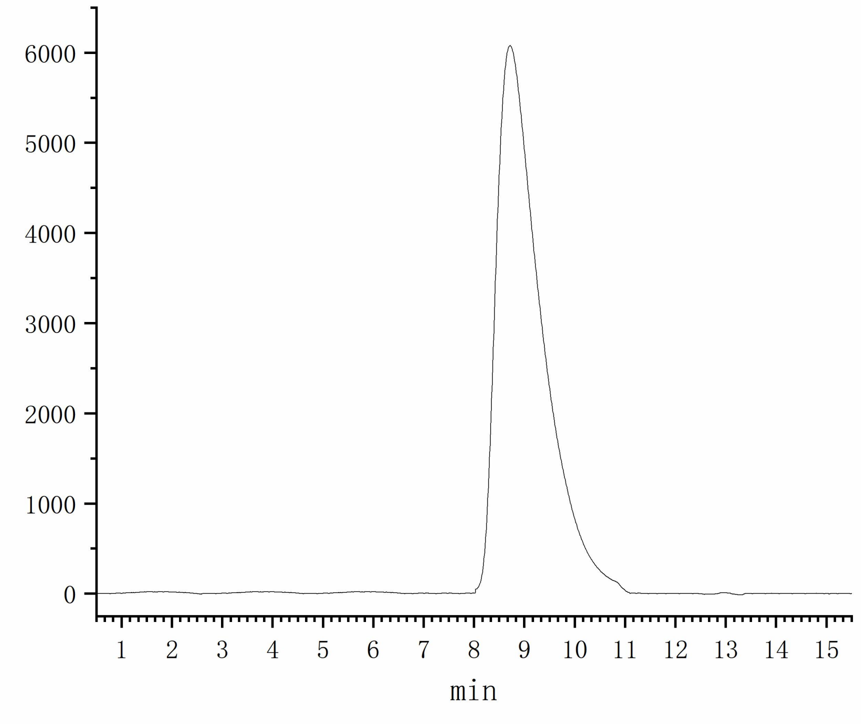

Figure S53 The HPLC chromatograms of **4**

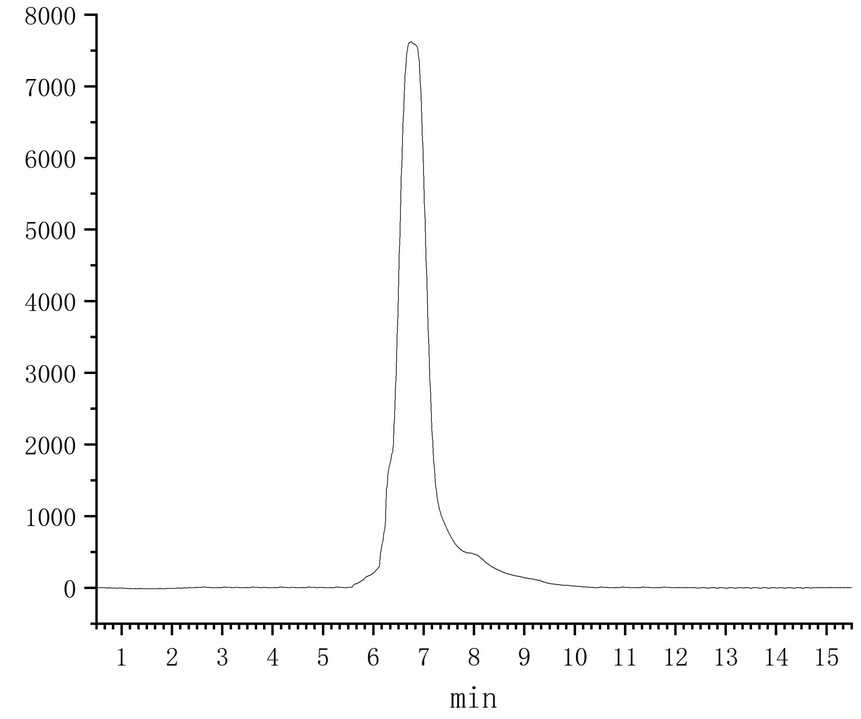

Figure S54 The HPLC chromatograms of **5**

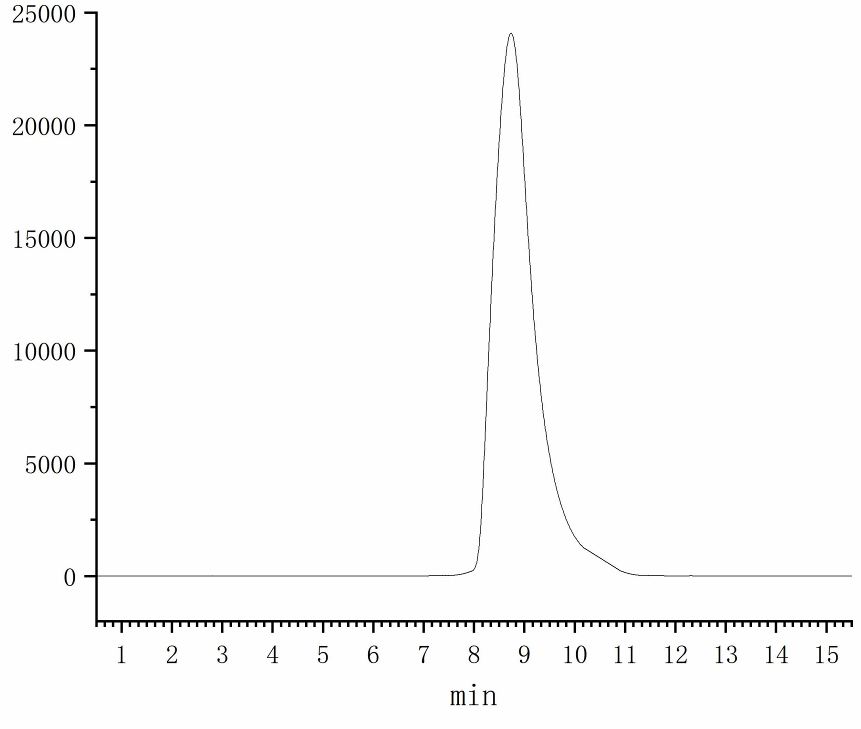

Figure S55 The HPLC chromatograms of **6**

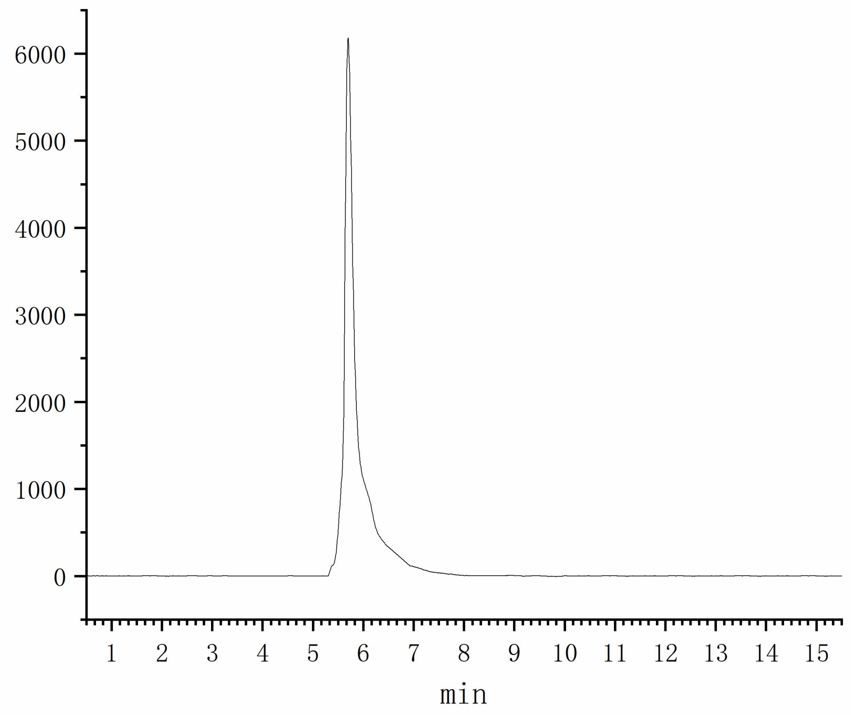

Figure S56 The HPLC chromatograms of **7**

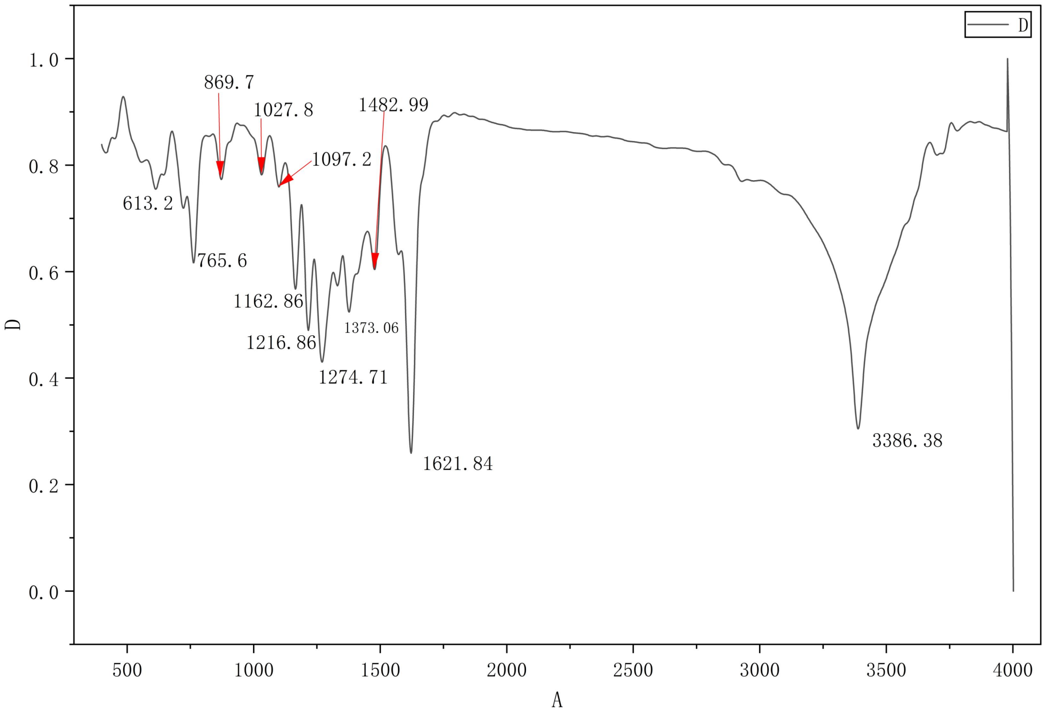

Figure S57 IR spectrum of **Emodin**

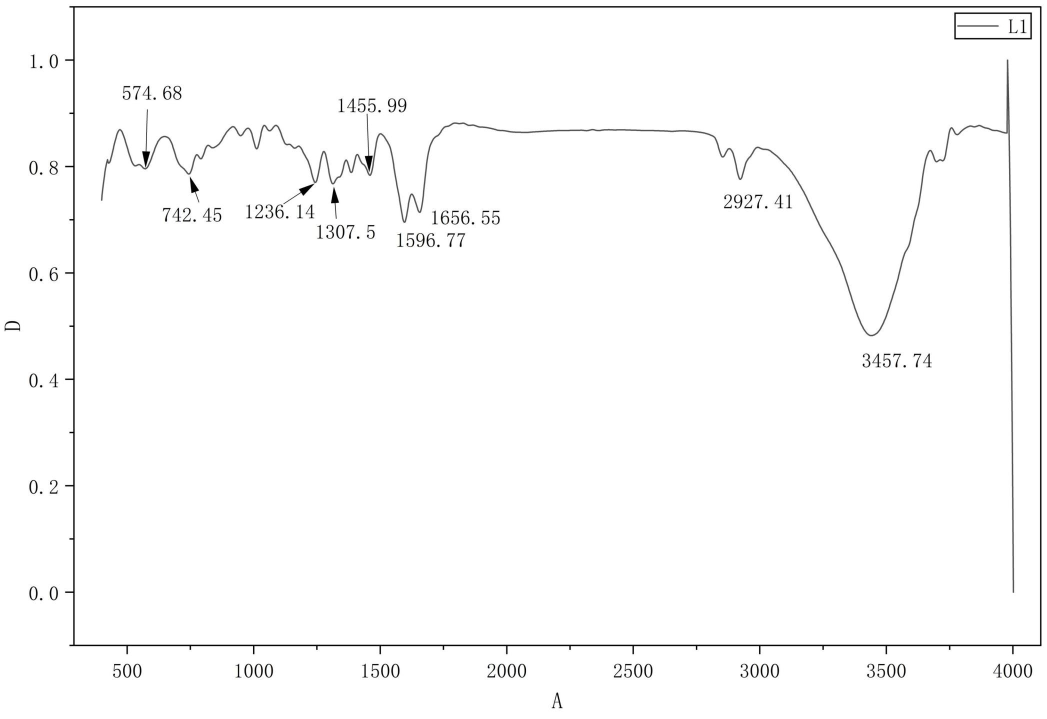

Figure S58 IR spectrum of **L1**

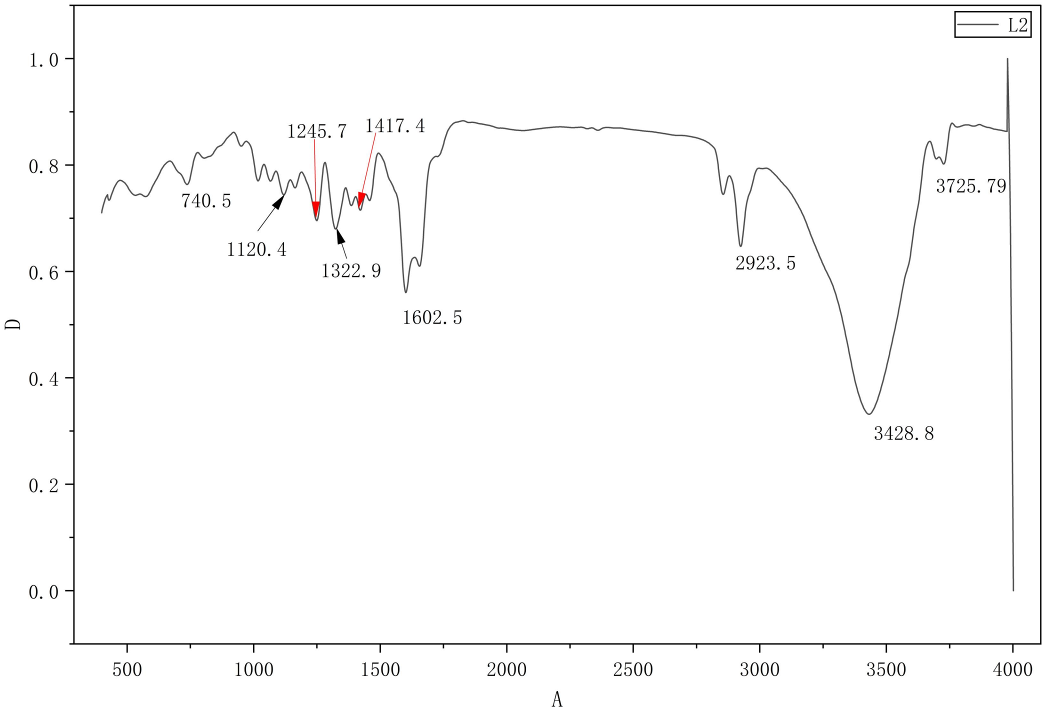

Figure S59 IR spectrum of **L2**

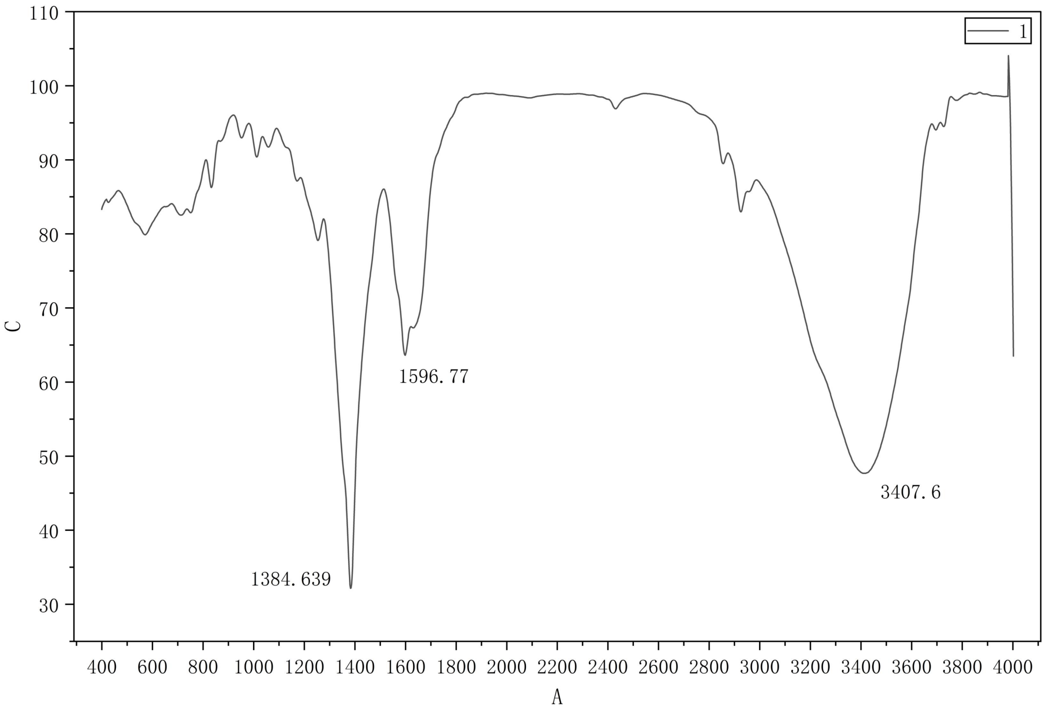

Figure S60 IR spectrum of **1**

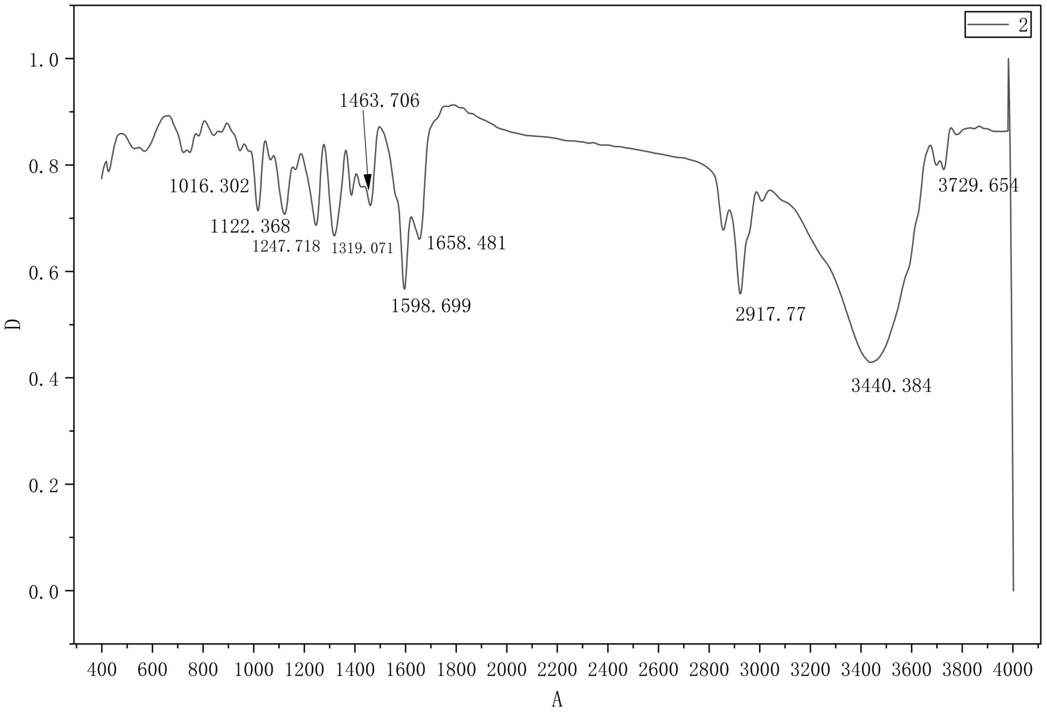

Figure S61 IR spectrum of **2**

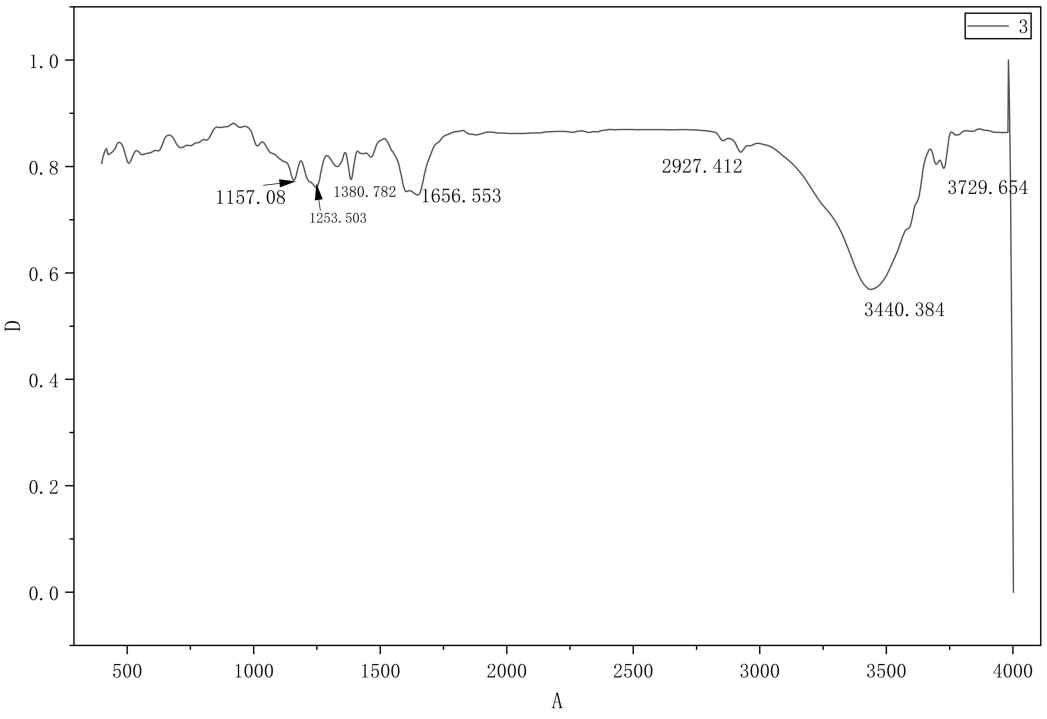

Figure S62 IR spectrum of **3**

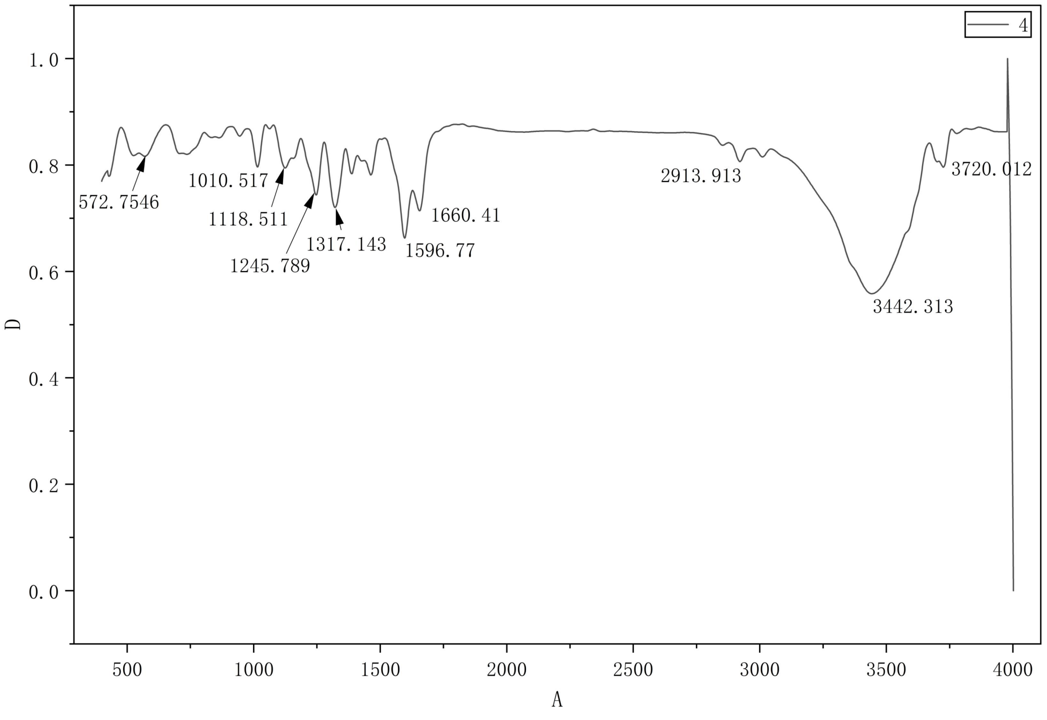

Figure S63 IR spectrum of **4**

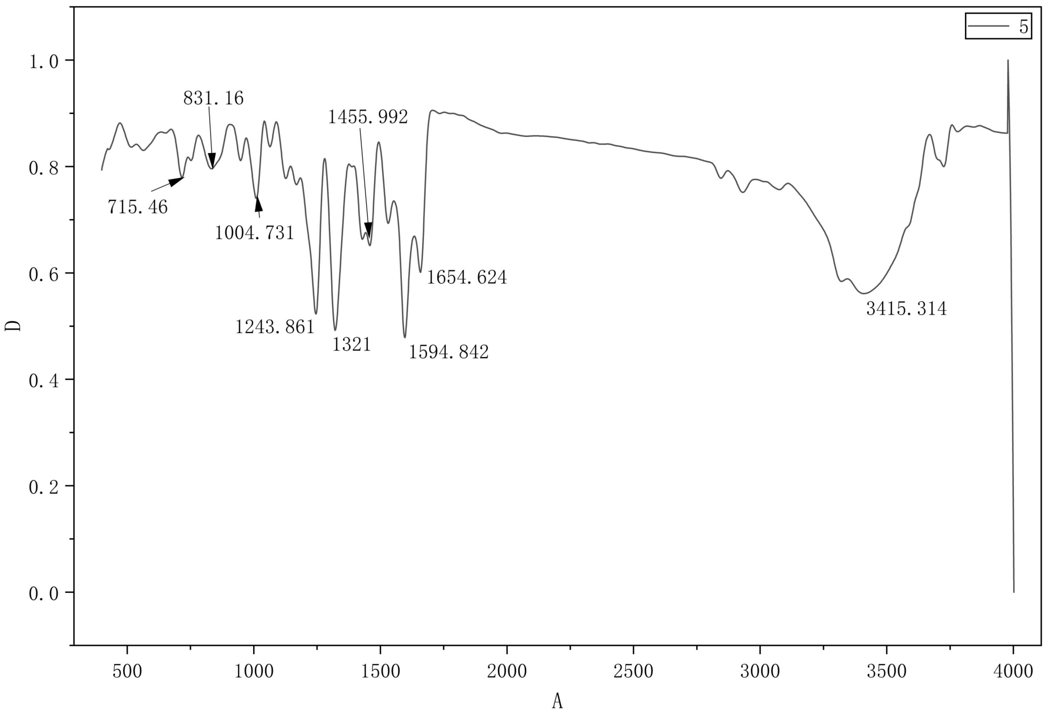

Figure S64 IR spectrum of **5**

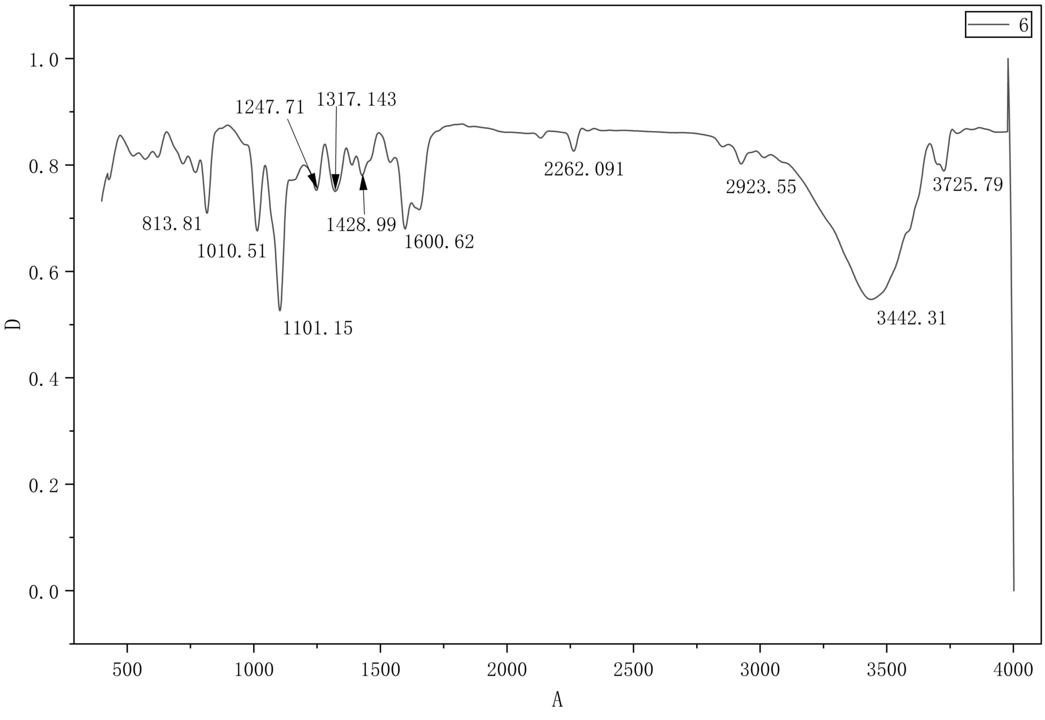

Figure S65 IR spectrum of **6**

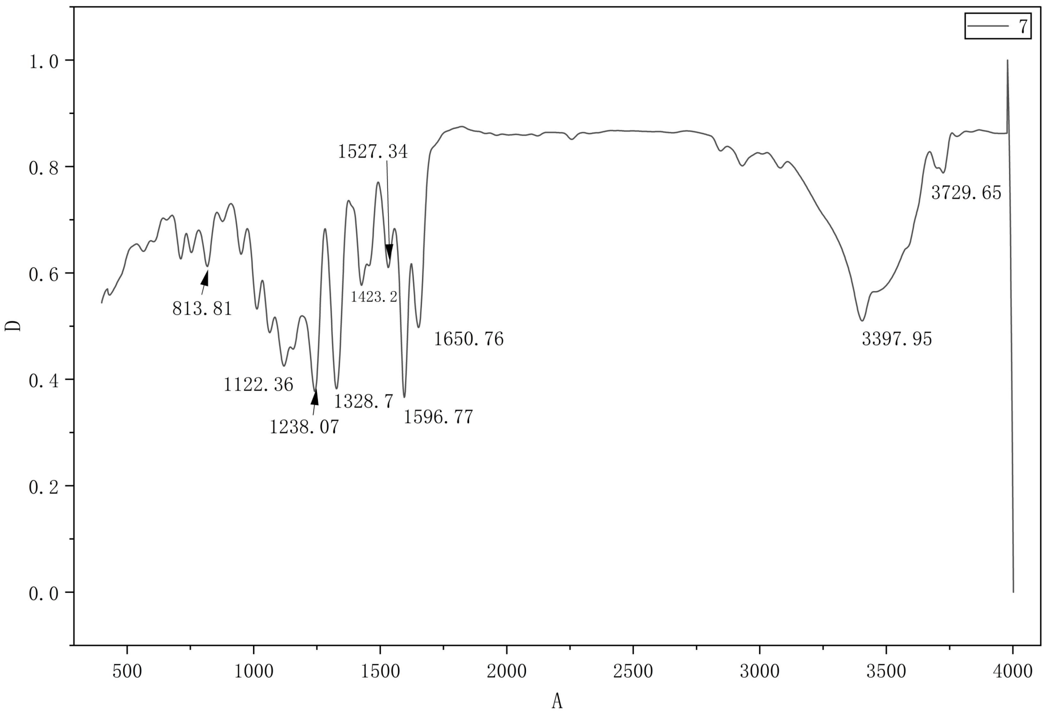

Figure S66 IR spectrum of **7**
